# Comparative genomics of the pathogenic ciliate *Ichthyophthirius multifiliis*, its free-living relatives and a host species provide insights into adoption of a parasitic lifestyle and prospects for disease control

**DOI:** 10.1186/gb-2011-12-10-r100

**Published:** 2011-10-17

**Authors:** Robert S Coyne, Linda Hannick, Dhanasekaran Shanmugam, Jessica B Hostetler, Daniel Brami, Vinita S Joardar, Justin Johnson, Diana Radune, Irtisha Singh, Jonathan H Badger, Ujjwal Kumar, Milton Saier, Yufeng Wang, Hong Cai, Jianying Gu, Michael W Mather, Akhil B Vaidya, David E Wilkes, Vidyalakshmi Rajagopalan, David J Asai, Chad G Pearson, Robert C Findly, Harry W Dickerson, Martin Wu, Cindy Martens, Yves Van de Peer, David S Roos, Donna M Cassidy-Hanley, Theodore G Clark

**Affiliations:** 1 Genomic Medicine, J Craig Venter Institute, 9704 Medical Center Dr., Rockville, MD 20850, USA; 2 Informatics, J Craig Venter Institute, 9704 Medical Center Dr., Rockville, MD 20850, USA; 3 Biology, University of Pennsylvania, 3451 Walnut St, Philadelphia, PA 19104, USA; 4 Joint Technology Center, J Craig Venter Institute, 9704 Medical Center Dr., Rockville, MD 20850, USA; 5 Informatics, J Craig Venter Institute, 10355 Science Center Drive, San Diego, CA 92121, USA; 6 Microbial and Environmental Genomics, J Craig Venter Institute, 9704 Medical Center Dr., Rockville, MD 20850, USA; 7 Microbial and Environmental Genomics, J Craig Venter Institute, 10355 Science Center Drive, San Diego, CA 92121, USA; 8 Biological Sciences, University of California - San Diego, 9500 Gilman Dr., La Jolla, CA 92093, USA; 9 Biology, University of Texas at San Antonio, 1 UTSA Circle, San Antonio, TX 78249, USA; 10 Microbiology and Immunology, Drexel University College of Medicine, 2900 Queen Lane, Philadelphia, PA 19129, USA; 11 Biological Sciences, Indiana University - South Bend, 1700 Mishawaka Avenue, South Bend, IN 46634, USA; 12 Undergraduate Science Education Program, Howard Hughes Medical Institute, 4000 Jones Bridge Road, Chevy Chase, MD 20815, USA; 13 Cell and Developmental Biology, University of Colorado - Denver, 13001 E. 17th Place, Aurora, CO 80045, USA; 14 Infectious Diseases, College of Veterinary Medicine, University of Georgia, 501 DW Brooks Dr, Athens, GA 30602, USA; 15 Department of Biology, University of Virginia, 485 McCormick Road, Charlottesville, VA 22903, USA; 16 Plant Systems Biology, Ghent University, Technologiepark 927, Ghent, B-9052, Belgium; 17 Biology and Penn Genome Frontiers Institute, University of Pennsylvania, 3451 Walnut St., Philadelphia, PA 19104, USA; 18 Microbiology and Immunology, College of Veterinary Medicine, Cornell University, C5 181 Veterinary Medical Center, Ithaca, NY 14853, USA

## Abstract

**Background:**

*Ichthyophthirius multifiliis*, commonly known as Ich, is a highly pathogenic ciliate responsible for 'white spot', a disease causing significant economic losses to the global aquaculture industry. Options for disease control are extremely limited, and Ich's obligate parasitic lifestyle makes experimental studies challenging. Unlike most well-studied protozoan parasites, Ich belongs to a phylum composed primarily of free-living members. Indeed, it is closely related to the model organism *Tetrahymena thermophila*. Genomic studies represent a promising strategy to reduce the impact of this disease and to understand the evolutionary transition to parasitism.

**Results:**

We report the sequencing, assembly and annotation of the Ich macronuclear genome. Compared with its free-living relative *T. thermophila*, the Ich genome is reduced approximately two-fold in length and gene density and three-fold in gene content. We analyzed in detail several gene classes with diverse functions in behavior, cellular function and host immunogenicity, including protein kinases, membrane transporters, proteases, surface antigens and cytoskeletal components and regulators. We also mapped by orthology Ich's metabolic pathways in comparison with other ciliates and a potential host organism, the zebrafish *Danio rerio*.

**Conclusions:**

Knowledge of the complete protein-coding and metabolic potential of Ich opens avenues for rational testing of therapeutic drugs that target functions essential to this parasite but not to its fish hosts. Also, a catalog of surface protein-encoding genes will facilitate development of more effective vaccines. The potential to use *T. thermophila *as a surrogate model offers promise toward controlling 'white spot' disease and understanding the adaptation to a parasitic lifestyle.

## Background

**Figure 1:**
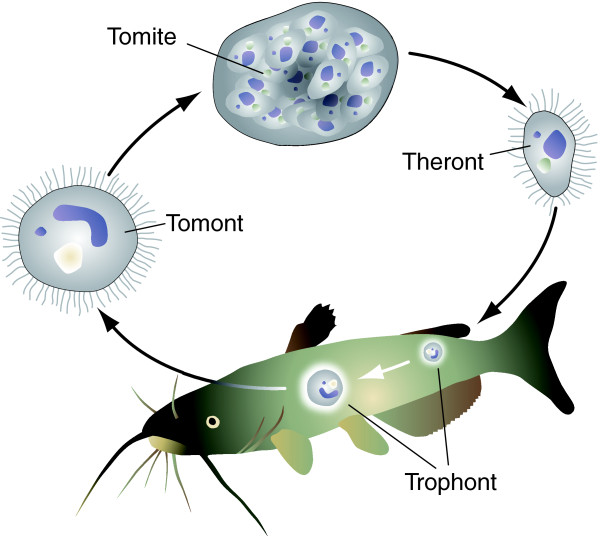
**Life cycle of Ich**. Infective theronts bore through the surface mucus and take up residence within the epithelium of susceptible fish. Theronts differentiate into feeding trophonts that grow and exit the host (as tomonts) within 4 to 7 days. Tomonts swim for a brief period and then adhere to an inert support where they secrete a gelatinous capsule. Tomonts divide within the capsule to form hundreds of tomites that differentiate into infective theronts within 18 to 24 hours at room temperature. Theronts that fail to infect fish die within 1 to 2 days.

The ciliates are an ancient and diverse phylogenetic group related to the largely parasitic apicomplexans, but consisting mostly of free-living heterotrophs. Some ciliates, however, have adopted a parasitic lifestyle. By far the most important of these is *Ichthyophthirius multifiliis *(which we will refer to by its common name of Ich), an endoparasite that causes white spot disease in freshwater fish [1,2]. With an extremely broad host-range, Ich is responsible for large-scale die-offs in natural populations and poses a significant threat to the growing worldwide aquaculture industry. Ich has a simple life cycle with no intermediate hosts (Figure 1). The free-swimming theront form invades the epidermis of susceptible fish, feeding on host tissue and growing up to 0.5 mm in diameter. Host-associated trophonts become visible as individual white spots for which this disease is named. A severe infection, particularly of the gills, results in asphyxiation and death. Although fish that survive infection are resistant to future challenge, prophylactic and therapeutic options remain extremely limited.

Experimental studies of Ich are limited by its obligate parasitic lifestyle and lack of genetics, and therefore genomic approaches have been pursued to identify targets for therapy and vaccines. EST projects [3,4] have provided partial sequences of many protein-coding genes, but to gain a complete understanding of Ich's metabolism and virulence, it is necessary to obtain and analyze its full genome sequence. Indeed, this approach has been extremely useful in uncovering potential targets for therapeutic intervention and/or immunologic protection for a number of protozoan parasites whose complete genome sequences have recently been deciphered [5-8]. Fortunately, Ich is fairly closely related to the model organisms *Tetrahymena thermophila *and *Paramecium tetraurelia*, whose macronuclear genomes have also been sequenced and annotated [9-11]. As shown here, comparative genomic analysis between these free-living species and the parasitic Ich reveals extensive genome reduction and modifications associated with the adoption of a parasitic lifestyle. There are relatively few cases of which we are aware in which the genome sequences of a parasite and a closely related free-living species are both available for such comparative analysis (for example, [12]). The ciliates may represent an excellent model system in which to explore the genomic consequences of this lifestyle switch, as it appears to have occurred in multiple independent cases within the genus *Tetrahymena *alone [13].

In addition, the genome of zebrafish, a model organism and representative host species, has been sequenced and thoroughly annotated [14]. Metabolic reconstruction of Ich and comparison with its host's metabolic pathways reveal potential targets for combating white spot disease.

## Results and discussion

### Genome sequencing

We selected for sequencing an Ich strain of the D serotype, the most prevalent in known infections. To minimize locus heterozygosity, the culture was initiated from a single parasite. Like most ciliate species, Ich is binucleate, having a presumably diploid germline micronucleus (MIC) and a polyploid somatic macronucleus (MAC). Because the MAC is the transcriptionally active nucleus, it was the focus of our sequencing efforts. By several independent methods (in particular, comparison of Southern blot hybridization intensities to known amounts of cloned and genomic DNA with a unique sequence probe), we estimated the Ich MAC genome size to be about 50 Mb (TG Clark, unpublished data), consistent with the 72 Mb and 104 Mb genome sizes of *P. tetraurelia *and *T. thermophila*, respectively.

In all ciliates studied to date, the MAC is derived from a copy of the MIC during sexual conjugation in a process that involves genome-wide DNA rearrangements, including chromosome fragmentation and the elimination of most or all repetitive, transposon-related sequences [15]. Therefore, we anticipated the MAC genome to consist of multiple chromosomes (*T. thermophila *has 181; E Orias and E Hamilton, personal communication) and to have a low level of repetitiveness. In the *Tetrahymena *genome project, MACs were physically separated from MICs, resulting in an assembly largely free of MIC-specific sequence contamination, but similar nuclear separation techniques have not been developed for Ich. Therefore, we relied on natural enrichment of the MAC genome; during the host-associated trophont stage of parasite development (Figure 1), endoduplication of the MAC genome occurs, leading to an estimated ploidy of up to 12,000 C, in the absence of MIC genome duplication [16].

**Figure 2:**
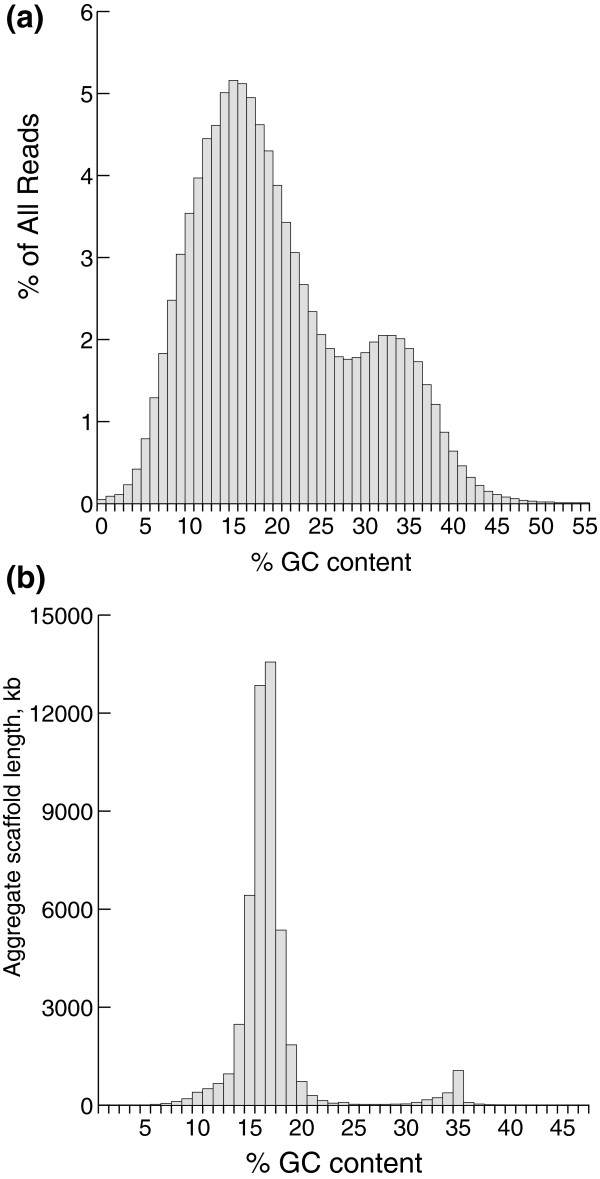
**GC content of reads and scaffolds**. **(a) **Percentage of GC content is plotted against percentage of combined quality Sanger and 454 reads of whole cell Ich DNA, showing a prominent shoulder of reads more GC-rich than expected for Ich. **(b) **Following assembly, mean GC content was plotted against the aggregate scaffold length within each percent GC bin, showing clean separation between scaffolds that make up the bacterial genomes and those that make up the much larger Ich genome.

Whole cell DNA was prepared from trophonts, taking care to minimize contamination from fish tissue or other associated microbes. Plasmid libraries were prepared with 2 to 4 kb and 4 to 6 kb insert size ranges for paired end sequencing. However, initial quality control of these libraries revealed a high proportion of reads with higher than expected GC content (Figure 2a) and sequence similarity to bacteria. Further analysis [17] made it clear that this Ich strain harbors multiple species of intracytoplasmic bacteria (which we will refer to as endosymbionts, although the nature of their relationship to their Ich host is unclear). Efforts to purify or selectively clone Ich DNA were unsuccessful, and therefore we decided to shotgun sequence and assemble the mixture and separate the genomes bioinformatically. This task was simplified by a dramatic difference in average GC content between Ich (approximately 15%) and the bacteria (approximately 34%). Presumably because of a bias against stable maintenance of AT-rich DNA in *Escherichia coli*, the plasmid libraries, especially the larger insert library, were heavily contaminated with bacterial sequence. We therefore focused most sequencing effort on pyrosequencing (454 FLX Titanium) supplemented by 2 to 4 kb paired end Sanger reads. The even distribution of read numbers on both sides of the approximately 15% GC Ich peak (Figure 2a) indicates that the total pool of reads is not significantly biased against GC-poor sequence content.

### Genome assembly and partitioning

All good quality Sanger and 454 reads were assembled using Celera Assembler Version 5.3 [18], generating 1,803 scaffolds of average length 27,320 bp. As shown by Figure 2b, these scaffolds can be almost completely partitioned on the basis of average GC content into two separate bins, one representing the very AT-rich ciliate genome and the other representing the genomes of endosymbiotic bacteria. As a first approximation, we drew the boundary between these bins at 26% GC and reran Celera v5.3 on the underlying reads, resulting in a slight improvement of the assemblies. To correct cases of inappropriate binning (especially near the 26% GC cutoff) and search for possible fish DNA contamination, we performed a MEGAN analysis [19] on all scaffolds to determine their phylogenetic affinities; several that showed similarity to known ciliate DNA sequences were moved from the symbiont bin to the Ich bin, but in general the partitioning was remarkably clean and little contamination was detected. Assembly and analysis of the endosymbiont reads will be described in a separate paper. We also searched for MIC contamination by BLAST-searching all contigs against known ciliate transposase sequences, but could detect no clear contamination. We cannot rule out the possibility of some MIC contamination, but available evidence suggests any such contamination would likely be less than that found in the initial *T. thermophila *assembly [11], which has been estimated at about 1% of the total length [10]. We can also not entirely rule out the presence of contamination from other sources, such as bacterial symbionts or fish host, in the current assembly; further efforts in genome closure would likely be the most effective means of eliminating any such contamination. The span of the final set of scaffolds was 49.0 Mb, in close agreement with our preliminary genome size estimate of 50 Mb.

Two Ich sequences not found in the initial assemblies were the ribosomal DNA (rDNA) locus and the mitochondrial DNA (mtDNA). Because these sequences were represented among the reads in much higher numbers than the average locus, the Celera Assembler excluded them as repetitive DNA, but they were assembled 'manually' as described in the Materials and methods section. The Ich rDNA locus encodes the large and small subunit rRNAs and, as in *Tetrahymena*, is located on its own small, highly amplified chromosome (accession ID GL985055). During *T. thermophila *MAC development, the single-copy MIC rDNA locus is excised and converted by an intramolecular recombination event into a 20 kb palindromic molecule with a short (28 bp) non-palindromic center [20]. Palindrome formation and gene amplification are characteristic of a number of developmental and disease-associated genomic events [21]. The Ich rDNA is also a palindrome, but lacks a non-palindromic center. It would be interesting to determine the Ich MIC sequence in the rDNA-terminal region and compare it with the corresponding *T. thermophila *region, which contains a pre-existing 42 bp inverted repeat, separated by a 28 bp non-palindromic center, that nucleates palindrome formation following chromosome fragmentation [22].

All sequenced ciliate mtDNA molecules are linear, and Ich's is no exception (accession ID JN227086). The non-telomeric portion of the molecule is 47,620 bp in length. Its structure and coding potential are described below. Linear mtDNAs found in ciliates and other species are capped by telomeres of varying lengths that consist of tandemly repeated units ranging up to 777 bp in length [23,24]. It is thought that these telomeres are maintained by unequal crossing over, which keeps their repeat sequences homogeneous but allows the rapid accumulation of interspecies differences. The mitochondrial telomeres of several *Tetrahymena *species have been sequenced [25,26]. Each species' characteristic repeat unit is between 31 and 53 bp and shares no identifiable sequence similarity with the others, except for the most closely related species. In some species, each end of the mtDNA is capped by a repeat unit unrelated to that found at the other end. The Ich mtDNA is terminated by identical repeat units at each end, in an inverted orientation. The repeat unit is 225 bp in length, substantially longer than those of known *Tetrahymena *species.

### Gap closure and optical mapping

Following the initial assembly and partitioning, standard autoclosure efforts resulted in closing 455 of the 540 Ich intra-scaffold gaps. Celera Assembler was rerun on the combined shotgun and finishing reads, resulting in a final draft assembly of 2,274 contigs in 2,017 scaffolds (this whole-genome shotgun project has been deposited at DDBJ/EMBL/GenBank under the accession AEDN00000000; the version described in this paper is the first version, AEDN01000000) with a contig N50 (the size that half the contigs are greater than) of 55,110 bp and average coverage depth of 19X. Additional assembly statistics are presented in Additional file 1. In *T. thermophila*, it appears that the MAC chromosomes, apart from the rDNA, are maintained at approximately equal copy number [11]. We plotted the mean depth of coverage for all Ich scaffolds against their sizes and found that they do not vary greatly, except for the expected stochastic variation found among the smallest scaffolds (Additional file 2). Thus, it appears that Ich chromosomes are also present in roughly equal copies, indicating that they are amplified to the same extent during trophont growth.

To gain a bigger picture of Ich MAC genome organization and lay the groundwork for future genome finishing efforts, we contracted with Opgen, Inc. to produce a whole genome ordered restriction map, or optical map [27]. This map revealed 69 complete linear chromosomes and four partial (single-ended) chromosomes; these four most likely represent the individual ends of two complete chromosomes that the mapping algorithm was unable to join. Thus, it appears that the Ich MAC genome consists of 71 chromosomes of between 1.5 Mb and 265 kb, plus an amplified 16.6 kb palindromic rDNA (which is too small to be optically mapped). The total length of the optical restriction map was 49.1 Mb, in close agreement with the complete span of our assembled scaffolds, 49.0 Mb, which argues that our genome assembly is largely complete.

We next attempted to map as many of our scaffolds as possible to the optical restriction map on the basis of their predicted restriction digest fragmentation patterns using two independent algorithms, OpGen's MapSolver and SOMA [28]. MapSolver placed 319 scaffolds and SOMA placed 555. Although the two algorithms generally agreed on the placement of larger scaffolds, there was disagreement in the placement of many smaller contigs (with fewer diagnostic restriction sites).

To evaluate scaffold placement further, we identified 121 scaffolds that ended in multiple copies of the telomeric repeat unit GGGGTT, close to the expected total of 142 (excepting the rDNA). Of these, 46 were found on scaffolds not placed on the optical map by either algorithm (Table S2b in Additional file 3). The remaining 75 mapped scaffolds ideally should only be found at the ends of chromosomes and in their proper orientation, but we found that almost one in four was either misplaced internal to the optical chromosome map or in improper orientation. By extension, we expect that many of the other placements, especially those with lower confidence (see Materials and methods) were also misplaced. We therefore decided to accept a scaffold placement on the optical map only if at least two lines of evidence (SOMA, MapSolver, and/or telomere) were in agreement. Using these stringent criteria, we were able to cover 53% (26.0 Mb) of the optical map by placement of 295 scaffolds (Table S2a in Additional file 3).

One scaffold (scaff_1120509250154, GL983437) that was placed at a unique optical map position (on partial chromosome 73) by both MapSolver and SOMA contains telomeric repeats but was not found in a chromosome-terminal position on the optical map. We examined the scaffold and found no indication of misassembly. Because the scaffold is large, contains a number of diagnostic restriction sites and maps uniquely by both algorithms, we suspect a misassembly of the optical map in this region resulted in its misplacement at a chromosome-internal position. This was a region of relatively lower fragment coverage in the map, which may be related to the failure to assemble a complete chromosome.

This optical mapping analysis provides substantial linkage information not discernible from the draft assembly and suggests multiple targets for future directed genome closure efforts by inter-scaffold PCR. This method also proved to be an efficient means of determining the total number and sizes of Ich MAC chromosomes. Optical map coverage appeared to be generally equal across all chromosomes, consistent with our conclusion from sequence coverage data that Ich MAC chromosome copy number does not vary widely.

### General features of protein-coding and non-coding RNA genes

#### Mitochondrial genome and ATP synthase

We annotated the Ich mitochondrial genome to identify 41 protein-coding genes, five tRNA genes, one split gene for small subunit rRNA and two inverted terminal copies of the split large subunit rRNA gene. Table 1 presents the full ordered list of predicted genes in the Ich mitochondrial genome in comparison with that of *T. thermophila*. While the nuclear genome of Ich has undergone significant contraction compared to its free-living relative (see below), the mitochondrial genome size, content and gene order are strikingly similar to those of *Tetrahymena *spp. [29-31]. Between 38 and 41 (depending on whether three poorly conserved gene pairs are indeed homologous) of *T. thermophila*'s 43 putative protein-coding ORFs are present in Ich and are found in the same order and orientation, except for a reversal of the first two (ymf66 and ymf57) and the relocation of the 'b' portion of the split *nad1 *gene. Ich also retains five of the eight predicted *Tetrahymena *tRNA genes, all in nearly the same locations and orientations, as well as the same configuration of rRNA genes, although the tRNA genes found between the split portions of the large subunit rRNA genes of *Tetrahymena *spp. and *P. tetraurelia *were unexpectedly absent. Thus, parasitic adaptation by Ich has resulted in no significant minimization of mitochondrial functions compared to its free-living relatives. This is in contrast to apicomplexan parasites, where extensive mtDNA gene losses and rearrangements have been common [32].

**Table 1:** Ordered list of Ich mitochondrial genes

Start	End	Accession number	Product name	Gene symbol	*T. thermophila* ^a^
823	742	IMG5_M206942	tRNA-Tyr-2	tRNA-Tyr-2	√
1217	941	IMG5_M206943	LSU rRNA	*rnl_a_1*	√
					tRNA-Leu
3687	1410	IMG5_M206944	LSU rRNA	*rnl_b_1*	√
5041	3695	IMG5_M206945	Ymf 66	*ymf66*^b^	*ymf57*^b^
5301	4999	IMG5_M206946	Ymf57	*ymf57*^b^	*ymf66*^b^
6607	5441	IMG5_M206947	Ymf76	*ymf76*	√
7446	6622	IMG5_M206948	Ribosomal protein S13	*rps13*	√
7903	7424	IMG5_M206949	Ribosomal protein S3	*rps3*	√
8202	7903	IMG5_M206950	Ribosomal protein S19	*rps19*	√
8996	8208	IMG5_M206951	Ribosomal protein L2	*rpl2*	√
9471	8977	IMG5_M206952	Hypothetical protein	Hypothetical^c^	*ymf74*
9961	9455	IMG5_M206953	NADH dehydrogenase subunit 10	*nad10*	√
10381	9977	IMG5_M206954	Ribosomal protein S12	*rps12*	√
10914	10393	IMG5_M206955	NADH dehydrogenase subunit 2	*nad2*	√
10922	12214	IMG5_M206956	NADH dehydrogenase subunit 7	*nad7*	√
12220	12522	IMG5_M206957	Ribosomal protein S14	*rps14*	√
12522	13082	IMG5_M206958	Ymf60	*ymf60*	√
14068	13079	IMG5_M206959	Ymf64	*ymf64*	√
14748	14170	IMG5_M206960	Ymf75	*ymf75*	√
14821	14750	IMG5_M206961	tRNA-Phe-1	tRNA-Phe	√
					*nad1_b*^b^
15055	14828	IMG5_M206962	ATP synthase F0 subunit 9	*atp9*	√
15863	15060	IMG5_M206963	Ymf63	*ymf63*	√
16966	15881	IMG5_M206964	Ymf65	*ymf65*	√
					*ymf69*
17653	17174	IMG5_M206965	Ymf59	*ymf59*	√
18096	17656	IMG5_M206966	Ribosomal protein L16	*rpl16*	√
19722	18163	IMG5_M206967	Heme maturase	*yejR*	√
20433	19726	IMG5_M206968	Ymf61	*ymf61*	√
					tRNA-His
20829	20464	IMG5_M206969	NADH dehydrogenase subunit 3	*nad3*	√
					*ymf72*
21193	20852	IMG5_M206970	Ymf58	*ymf58*	√
21795	21214	IMG5_M206971	NADH dehydrogenase subunit 9	*nad9*	√
25990	21839	IMG5_M206972	Ymf77	*ymf77*	√
28327	28148	IMG5_M206973	NADH dehydrogenase subunit 1	*nad1_b*^b^	
28510	29802	IMG5_M206974	Apocytochrome b	*cob*	√
29792	31885	IMG5_M206975	NADH dehydrogenase subunit 5	*nad5*	√
31902	33521	IMG5_M206976	Cytochrome c oxidase subunit 2	*cox2*	√
33532	33735	IMG5_M206977	SSU rRNA	*rns_a*	√
33783	35202	IMG5_M206978	SSU rRNA	*rns_b*	√
35555	36841	IMG5_M206980	Ymf67	*ymf67*	√
36862	36932	IMG5_M206981	tRNA-Trp-1	tRNA-Trp	√
36961	38583	IMG5_M206982	Ymf68	*ymf68*	√
38587	38865	IMG5_M206983	Hypothetical protein	Hypothetical^c^	*ymf71*
38942	41014	IMG5_M206984	Cytochrome c oxidase subunit 1	*cox1*	√
41015	41923	IMG5_M206985	NADH dehydrogenase subunit 1	*nad1_a*	√
41847	42605	IMG5_M206986	Ymf62	*ymf62*	√
42627	42986	IMG5_M206987	Ribosomal protein L14	*rpl14*	√
					tRNA-Glu^b^
43012	43278	IMG5_M206988	Ymf70	*ymf70*	√
43284	44807	IMG5_M206989	NADH dehydrogenase subunit 4	*nad4*	√
44811	45284	IMG5_M206990	Ymf73	*ymf73*	√
45323	45393	IMG5_M206991	tRNA-Glu-1	tRNA-Glu^b^	
45404	47681	IMG5_M206992	LSU rRNA	*rnl_b_2*	√
					tRNA-Leu
47874	48150	IMG5_M206993	LSU rRNA	*rnl_a_2*	√
48267	48348	IMG5_M206994	tRNA-Tyr-1	tRNA-Tyr-1	tRNA-Met

^a^A checkmark indicates that the *T. thermophila *mtDNA contains a homolog of the same gene in the same relative position and orientation. ^b^Divergent gene order. ^c^'Hypothetical' indicates insufficient evidence to assign gene name.

This close correspondence between the Ich and *Tetrahymena *mitochondrial genomes may extend also to the nuclear genes encoding complexes of the mitochondrial inner membrane. A recent study examined the structure and composition of the *T. thermophila *ATP synthase, finding a striking number of novel subunits, in addition to conservation of the core F_1 _subunits and the F_O _c subunit that forms the rotational ring in the inner membrane [33]. A comparative search of the Ich nuclear and mitochondrial genomes indicates that all 22 subunits identified in the *T. thermophila *study have an ortholog or, in the case of the alpha and beta subunits, two orthologs in Ich (Additional file 4). Because the ciliate ATP synthase is so dramatically divergent from the corresponding vertebrate enzyme complex and is undoubtedly essential for Ich survival, it presents a highly attractive drug target [34].

#### MAC genome

By a combination of automated and manual genome annotation, we modeled the protein-coding genes of Ich. Predictions were tested and refined by alignment to existing ESTs. In addition, we generated new transcriptome data by paired-end Illumina sequencing (RNA-seq) of a normalized polyA+ cDNA library prepared from pooled theront and trophont RNAs. Over 99% of the RNA-seq assemblies aligned to our genome assembly (see Materials and methods), arguing again that the assembly is largely complete. In total, we predicted 8,096 protein-coding genes, about one third as many as found in the most closely related sequenced ciliate, the free-living *T. thermophila *[11]. This result suggests extensive genome reduction has occurred in the course of Ich's adaptation to a parasitic lifestyle. General characteristics of the predicted genes in comparison to *T. thermophila *are presented in Table 2. Besides the reduction in gene number, the predicted genes of Ich are significantly shorter than those of *T. thermophila *in both coding and non-coding length. In summary, compared with the genome of its nearest sequenced free-living relative, the Ich genome is reduced approximately two-fold in size, three-fold in gene content and two-fold in gene density. The overall GC content of the Ich macronuclear genome (15.9%) is the lowest yet reported for any fully sequenced eukaryote [35] and significantly lower than that of *T. thermophila *(22.3%), but the discrepancy in exon GC content is not as great (24.1% versus 27.5%, respectively), suggesting the possibility that AT mutational bias acting against reduced selection in the gene-poor Ich genome may be driving GC content to extremely low levels.

**Table 2:** Important genome statistics

	Ich	*T. thermophila*
**Genome**		
Total predicted genes	8,096	24,725
Percentage coding	21.0	47.8
Total annotated sequence length^a^	47,869,613	103,002,206
Percentage GC	15.9	22.3
**Genes**		
Longest gene	21,958	47,333
Shortest gene	152	119
Total gene length	13,277,109	62,628,433
Average gene length^b^	1,639	2,533
Average gene coding sequence	1,243	1,989
Gene density (per 10,000 bp)	16.92	41.6
Percentage genes with introns	79.0	71.4
**Exons**		
Total exon length	10,666,748	49,184,519
Total number of exons	29,479	114,215
Longest exon	11,206	14,389
Shortest exon	2	2
Average exon length	361.8	430.6
Percentage GC	24.1	27.5
**Introns**		
Total number of introns	21,380	89,490
Longest intron	11,437	13,045
Shortest intron	16	18
Average intron length	152.8	162.7
Average introns per gene	2.6	3.6
Percentage GC	12.3	16.1
**Intergenic regions**		
Total intergenic regions	33,985,751	39,886,399
Shortest intergenic region	2	2
Longest intergenic region	42,323	46,152
Average intergenic length	3,650	1,562
Percentage GC	13.9	18.1

^a^Scaffolds < 2 kb in length were not annotated. ^b^Not including 5' and 3' untranslated regions.

Because of its close association with bacterial endosymbionts, we addressed the possibility of horizontal gene transfer (HGT) into the Ich genome using the APIS program [11], a pipeline for automatic construction and interpretation of phylogenetic trees. Each query predicted protein was compared to a database of proteins from complete genomes using BLASTP. Sequences of homologs with high BLAST similarity (e-value ≤ 10^-9^) were retrieved and a multiple sequence alignment constructed for the purpose of inferring a neighbor-joining phylogenetic tree. The trees were parsed to determine the phylogenetic placement of each query. Only trees for 10 Ich genes are contained within clades of genes of entirely bacterial or archaeal origin and only 17 Ich genes are outgroups of bacterial clades. Even when initial clading with *T. thermophila *and *P. tetraurelia *is excluded from the analysis (in order to look for bacterial genes that may have been acquired by the common ancestor of these organisms), only 160 genes are identified as candidates for cases of HGT. Because the principal Ich endosymbiont is a member of the Rickettsiales, we searched the trees of these 160 candidates for genes of rickettsial origin and found only seven. Examination of the tree topologies of these seven genes revealed no strong evidence suggesting lineage-specific HGT between Ich and its principal endosymbiont. Separate genomic analysis of the principal endosymbiont of this Ich strain (M. Lynch *et al*., in preparation) failed to reveal HGT from host to symbiont, suggesting this does not account for the genome reduction we observe in Ich.

There is extensive, clear-cut evidence for multiple whole genome duplications (WGDs) in the evolutionary history of *P. tetraurelia*, a more distant relative of *T. thermophila *and Ich [9]. Phylogenetic analysis suggested that the earliest detectable such WGD occurred prior to the split between the lineages leading to *Paramecium *and *Tetrahymena*. However, independent analysis failed to detect evidence of WGD in *T. thermophila *[11]. With the sequencing of another ciliate along the *Tetrahymena *lineage, we reconsidered the timing of WGD events, using algorithms designed to detect the remnants of such events in the form of short blocks of intra-genomic synteny [36]. As expected, there is strong evidence for such blocks in the *P. tetraurelia *genome, but their numbers in the *T. thermophila *and Ich genomes are not above those expected by chance. Thus, we have failed to detect evidence for WGD on the Ich/*Tetrahymena *branch, although it is possible that the genome reduction experienced by Ich may have obscured the evidence in this species.

#### Non-coding RNAs

The Ich genome contains the expected complement of non-coding RNA (ncRNA) genes (Additional file 5), but in reduced numbers compared with its free-living relative *T. thermophila*. There are 144 predicted tRNA genes in the MAC genome and 5 in the mitochondrial genome, compared with 710 and 8, respectively, in *T. thermophila*. As expected, several tRNAs (6 and 2, respectively) have anticodons for translation of the alternative glutamine codons UAA and UAG [37]. We also detected a tRNA predicted to translate UGA as selenocysteine, strongly suggesting that Ich, like *T. thermophila *[11], has the potential to translate all 64 codons into amino acids.

The Ich genome contains only 13 predicted 5S rRNA genes, compared with about 155 predicted functional genes in *T. thermophila*. There is also an approximately three-fold reduction in the number of various ncRNAs that function in mRNA processing and protein trafficking. Ich does not appear to contain a variant U2 small nuclear ribonucleic acid (snRNA) similar to that found in the *T. thermophila *genome [11].

#### Codon usage

It is common for organisms to display bias in the frequency with which synonymous codons are employed. In some organisms, including *T. thermophila *[11], a subset of highly expressed genes displays additional bias, thought to result from selection for high translational efficiency and/or accuracy [38]. Such selection is weak and its effect on codon usage bias can be overwhelmed by random genetic drift in a species with a low effective population size [39], as appears to have occurred in the apicomplexan parasite *Plasmodium falciparum *[11]. We analyzed codon usage patterns in Ich using principal component analysis and found that, as in *P. falciparum*, there does not appear to be a subset of genes that uses a preferred codon set substantially different than that used by the average gene (Figure S2a in Additional file 6). In general, codon usage follows the pattern predicted by variation in GC3 content (the fraction of codons that are synonymous at the third codon position that have either a guanine or a cytosine at that position) alone (Figure S2b in Additional file 6). These observations may reflect a low effective population size of Ich, as a result of its obligate parasitic lifestyle. Mating of Ich has not been observed, and its frequency in the wild is unknown.

#### Ich ortholog grouping

**Figure 3:**
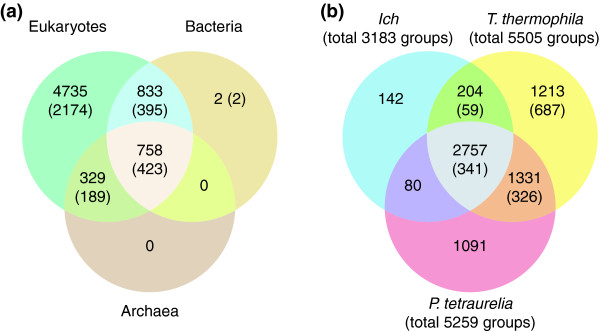
**Ortholog grouping**. **(a) **Phyletic grouping of Ich genes with previously grouped orthologs from other species. Numbers in parentheses indicate the total number of ortholog groups to which the respective genes belong. **(b) **Shared orthology between the three ciliate genomes. The numbers depict the total number of ortholog groups in each category. The numbers within the brackets indicate ortholog groups specific for *Tetrahymena *only and accordingly there are 341 ciliate specific ortholog groups shared with all 3 ciliates compared here. See Additional file 6 for more details regarding shared orthology between the three ciliates.

A useful approach for surveying the protein-coding gene landscape of a newly sequenced genome is to group genes by orthology, which can provide guidance for functional annotation and, in the case of parasites such as Ich, facilitate the identification of candidates for drug and/or vaccine development [40]. For this study, we grouped the Ich proteome with the 138 other species contained in the OrthoMCL database (OrthoMCL DB version 4) [41] using a one-way Blast search against all proteins contained therein; 7,382 Ich genes had orthologs in at least one other species and could be grouped into 3,183 ortholog groups, with an overwhelming majority sharing orthology with ciliates and other eukaryotic organisms (Figure 3a; Additional file 7). The remaining Ich genes did not satisfy the pairing cutoff criteria (e-value ≤ 10^-5 ^and matching at least 50% of the query protein). Nearly all the 3,183 groups include representatives from other eukaryotes (Figure 3a; Additional file 7), consistent with our failure to detect significant bacterial HGT. Additional file 7 gives a list of all Ich genes mapped to their ortholog hits.

Not surprisingly, most of the best matches to Ich predicted proteins (6,991 of 7,382) were *T. thermophila *predicted proteins, including 971 that mapped to 393 groups containing only *Tetrahymena *proteins (685 of these Ich proteins paired with previously ungrouped *Tetrahymena *proteins). A large fraction of the Ich genes that grouped with only *Tetrahymena *genes also grouped with *P. tetraurelia *genes and hence constitute ciliate-specific genes (Additional file 8; note that *P. tetraurelia *is not included in the current version of OrthoMCL DB and hence its ortholog grouping was carried out in similar fashion to Ich). Figure 3b displays shared orthology between the three sequenced ciliates. Of the 3,183 Ich-containing ortholog groups, 87% (2,758 groups mapped to 5,996 Ich genes) include both *Tetrahymena *and *Paramecium *genes and an additional 9% include genes from one or the other free-living ciliate. Only 142 ortholog groups (mapped to 204 Ich genes) excluded other ciliate genes while mapping to genes from other species. Among these, there are 30 ortholog groups (mapped to 35 Ich genes) specific to apicomplexan species, containing mostly proteins of unknown function. The remaining 112 ortholog groups have no obvious phylogenetic bias, and while a large fraction of these contain proteins of unknown function, a substantial number are enriched in proteins with enzymatic activity (Additional file 7).

Among the three ciliates, Ich has the fewest protein-coding genes, but ortholog grouping shows this is not entirely due to higher redundancy in *Tetrahymena *and *Paramecium *within a similar set of functional categories. Ich possesses the core ciliate proteome, mostly shared with *Tetrahymena *and *Paramecium*, but lacks orthologs for a significant number of genes shared by these ciliates and other forms of life. Figure 3b shows that 3,635 *Tetrahymena *and/or *Paramecium *ortholog groups exclude Ich (a number greater than the total number of Ich-containing ortholog groups). The genes contained in these Ich-excluded ortholog groups are significantly enriched in functional categories such as transcription factors, nucleic acid binding/metabolism and signaling pathways (including protein kinases; see below), suggesting that Ich may have limited redundancy in its ability to regulate cellular processes using intracellular signaling and transcriptional pathways compared with free-living ciliates. Ich has only 26 genes that group into 12 Ich-specific in-paralog groups (Additional file 8), dramatically fewer than *Tetrahymena *(2,805 genes in 687 groups; numbers after removing orthologs of Ich and *Paramecium*, which are not yet included in OrthoMCL DB) and *Paramecium *(3,758 genes in 1,163 groups; in comparison to *Tetrahymena *only), suggesting again that Ich has lost many of the ciliate-specific gene families and expansions seen in free-living ciliates. Clearly, Ich contains a streamlined ciliate genome suited to a parasitic life style.

Based on orthology, the largest group of functionally related proteins in the Ich genome are the kinases with 145 ortholog groups containing 602 potential kinases. An additional 69 potential kinases (see below) have ortholog best hits with previously ungrouped genes. Other large, functionally related gene families include the proteases and ion channels (see below). A significantly large portion of the Ich genome is devoted to enzymes (1,854 genes with four digit EC numbers in 763 ortholog groups; this set also includes kinases and other non-metabolic pathway enzymes having four digit EC numbers; Additional file 7). Enzyme assignments were used to reconstruct Ich metabolism and suggest potential candidates for drug development (see below).

### Analysis of selected gene families

#### Protein kinases

Throughout the tree of life, numerous sensory and regulatory functions are carried out by diverse protein kinases. Ich's closest sequenced relative, *T. thermophila*, devotes an unusually large portion of its proteome (3.8%) to kinases, including notable gene expansions of kinases associated with mitotic and cytoskeletal functions, as well as sensory histidine protein kinases [11]. By a combination of two methods (see Materials and methods), we identified 671 putative Ich kinase genes. Thus, remarkably, Ich devotes over 8% of its proteome to kinases (Table 3). Phylogenetic profiling of these 671 genes shows that 536 have only eukaryotic orthologs, 54 have shared orthology with bacteria and eukaryotes, 5 with archaea and eukaryotes and 7 with all three kingdoms. None shared orthology exclusively with bacteria or archaea or both. There are 103 Ich kinase genes that grouped only with *T. thermophila *or with *T. thermophila *plus *P. tetraurelia *and therefore may represent ciliate-specific kinases (Additional file 9).

**Table 3:** Major kinase groups of Ich compared with other species

Kinase group	*Ich*	*T. the*	*P. tet*	P. fal*	T. gon*	*S. cer*	*C. ele*	*D. rer*	*H. sap*
AGC	78 (8)	51 (8)	219 (8)	6 (4)	11 (4)	17 (6)	29 (16)	75 (13)	63 (15)
Atypical	37 (5)	103 (6)	270 (6)	5 (3)	5 (2)	14 (6)	18 (7)	47 (12)	38 (12)
CAMK	68 (5)	62 (6)	442 (6)	13 (4)	23 (3)	22 (4)	40 (14)	95 (17)	74 (18)
CK1	24 (1)	19 (1)	125 (1)	3 (1)	3 (1)	4 (1)	83 (12)	14 (3)	12 (3)
CMGC	68 (9)	61 (10)	199 (9)	16 (8)	21 (6)	23 (8)	48 (10)	65 (9)	63 (9)
Other	330 (24)	747 (45)	1,449 (40)	28 (6)	71 (11)	37 (20)	67 (27)	80 (30)	81 (34)
RSK	2 (1)	1 (1)	5 (1)	0 (0)	0 (0)	0 (0)	0 (0)	0 (0)	0 (0)
RGC	0 (0)	0 (0)	0 (0)	0 (0)	0 (0)	0 (0)	27 (1)	8 (1)	5 (1)
STE	13 (4)	19 (4)	39 (4)	1 (1)	1 (1)	14 (3)	24 (3)	51 (4)	47 (4)
TK	0 (0)	0 (0)	0 (0)	0 (0)	0 (0)	0 (0)	84 (18)	107 (28)	90 (29)
TKL	13 (3)	5 (3)	15 (3)	4 (1)	7 (1)	0 (0)	15 (5)	15 (7)	45 (8)
Unclassified (PF00069)	38 (1)	0 (0)	0 (0)	36 (1)	23 (1)	0 (0)	0 (0)	0 (0)	0 (0)
Total	671 (61)	1,068 (84)	2,763 (78)	112 (29)	165 (30)	131 (48)	435 (113)	557 (124)	518 (133)
Percentage of proteome	8.29	3.89	8.47	2.04	1.83	1.96	2.15	2.31	2.19

Total number of genes mapped to each kinase group; in parentheses, number of different families. An expanded list of all families under each kinase group is shown in Additional file 7. *T. the*, *T. thermophila*; *P. tet*, *P. tetraurelia*; *P. fal*, *Plasmodium falciparum*; *T. gon*, *Toxoplasma gondii*; *S. cer*, *Saccharomyces cerevisiae*; *C. ele*, *Caenorhabditis elegans*; *D. rer*, *Danio rerio*; *H. sap*, *Homo sapiens*. *Data for these two species was obtained from published reports [117,118] rather than from kinbase [116] or by orthology to the kinbase data, as for the other species.

Table 3 provides a summary of the Ich kinome showing the number of genes that can be grouped into various kinase families in comparison to free-living ciliates and other organisms. It is clear that members of phylum Ciliata devote a larger fraction of their proteome to kinases than most other species; however, ciliate kinases tend to map to fewer unique families (see numbers within parentheses in Table 3). While a large proportion of the ciliate kinases map into ciliate-specific familes (Additional file 9), others more or less follow a similar distribution to other unicellular eukaryotes and differ from metazoan kinomes in lacking membership in various tyrosine kinase families. In addition, Ich possesses 38 genes that contain the protein kinase Pfam domain but cannot be reliably grouped with previously known kinase families despite having orthologs in other species. These are likely to be pseudokinases with partial and/or inactive kinase domains (see Additional file 9 for a detailed list of all kinase families mapped to Ich and comparison to other species and Additional file 7 for a complete list of all Ich kinases and their phyletic associations). Overall, the Ich kinome is similar to those of free-living ciliates, except somewhat reduced in both genes and kinase families.

Ciliates, including Ich, display dramatic expansion of certain kinase families as well as containing many that are ciliate specific. The most prominent expansions are: Akt, AktR and nuclear Dbf2-related (NDR) families from the AGC group; the atypical histidine kinase family; the Ca2+/calmodulin-dependent protein kinase (CAMK)1, CAMKL and calcium-dependent protein kinase (CDPK) families of the CAMK group; the casein kinase 1 (CK1) family of the CK1 group; and Aur, NEK, polo-like kinase 1 (PLK1) and Unc-51-like kinase (ULK) families from the Other group of kinases (Additional file 9). These kinases affect a wide variety of cellular functions ranging from mitotic cell division (Aur/PLK), to cytoskeletal dynamics (ULK, NEK), two-component signaling (histidine kinases) and calcium and calmodulin regulated processes (the CAMK group). It is interesting to note that the CDPK kinases (also expanded in apicomplexan parasites) and histidine kinases are completely absent in higher vertebrates and thus obvious potential anti-parasitic drug targets [42]. Certainly, understanding the function and regulation of the Ich and other ciliate kinomes will play a large part in furthering our understanding of the biology of the Ich parasite as a whole.

#### Immobilization antigens

Immobilization antigens are abundant glycosylphosphatidylinositol (GPI)-anchored proteins that coat the surfaces of holotrichous ciliates [43]. While their precise function is unknown, i-antigens are the principal targets of the host immune response to infection and therefore attractive candidates for vaccine development against Ich. Despite this promise, the existence of serotype variation resulting from the expression of different i-antigens in natural parasite populations represents a potential bottleneck to their development as vaccines [43,44]. The underlying basis of serotype variation in Ich is poorly understood but could arise through differential expression of large numbers of i-antigen genes that are shared among isolates (antigenic shift), or the stable expression of a limited number of paralogous genes that undergo antigenic drift, or perhaps both. The free-living ciliates *T. thermophila *and *P. tetraurelia *contain families of related i-antigen alleles that are expressed in a mutually exclusive fashion in response to environmental stimuli. By contrast, only three i-antigen genes have been characterized in Ich to date [43,45]. One of these, *IAG52A[G5] *(AF324424) has been identified in multiple serotypes but is only weakly expressed [45] (DM Cassidy-Hanley, TG Clark, *et al*., unpublished). The other two are highly expressed and encode the serotype A and D antigens, respectively. The serotype A gene (designated *IAG48*; AF40273) was identified in parasite isolate G1, while the serotype D gene (designated *IAG52B*; AF405431) was identified several years ago in the G5 isolate described here [40]. Since the total number of i-antigen genes was unknown, sequencing of the MAC genome offered an unparalleled opportunity to analyze the potential for antigenic variation within any given strain.

**Figure 4:**
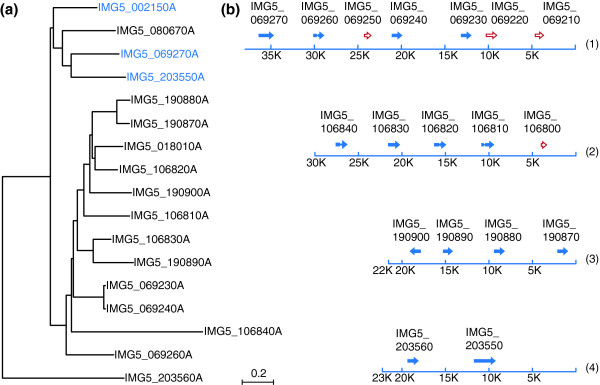
**Immobilization antigens**. **(a) **Unrooted neighbor-joining phylogenetic tree [123] of the 17 Ich immobilization antigen genes. The three described in the text as being related to previously identified genes are in blue. **(b) **Scaffolds containing arrays or tandem duplications of putative immobilization antigen genes (blue arrows) and pseudogenes (red outlined arrows). Locus accessions identify the individual genes. GenBank accession numbers for scaffolds 1 to 4 are as follows: 1, GL983567; 2, GL983846; 3, GL984331; 4, GL984394.

At the primary amino acid sequence level, the previously characterized Ich i-antigens are 40 to 57% identical, and share the same overall structure, consisting of conserved hydrophobic stretches at their amino and carboxyl termini (acting as signal peptides for membrane translocation and GPI-anchor addition, respectively) and 5 to 6 tandem repeats (60 to 100 amino acids in length) containing periodic cysteines. A search of the Ich MAC genome based on these features (see Materials and methods for details) yielded 17 candidate i-antigen genes (Figure 4; Additional file 10), and four (IMG5_069210, IMG5_069220, IMG5_069250, IMG5_106800) apparent pseudogenes. This is roughly proportional to the number of i-antigen genes in *T. thermophila *(34) when compared with the total numbers of genes in each species (approximately 1:3). At the nucleotide sequence level, two genes, IMG5_069270 and IMG5_002150, closely matched the previously characterized *IAG52A *and *IAG52B *genes, respectively. However, several differences were apparent, including six nonsynonymous base pair changes in the IMG5_069270 gene, and nine nonsynonymous base pair changes along with a 6 bp deletion in the IMG5_002150 gene. Because the G5 isolate was propagated from a single cell and was maintained in continuous culture since the genes were first sequenced in 2002, these variations are due either to cloning artifacts associated with the originally published sequences or rapid genetic drift over a period of about 7 years.

The newly identified gene most closely related to the previously characterized *IAG48 *serotype A gene is IMG5_203550 (63% identity, 85% similarity). It will be interesting to determine whether IMG5_203550 in fact encodes a serotype A antigen. If so, then the G5 isolate (which became senescent in 2009) had the potential to undergo antigenic shift to serotype A. By analogy it will be interesting to determine whether any of the other i-antigen genes described here are expressed in geographically distinct Ich isolates and whether they determine variant serotypes in these strains. In this regard, Ich i-antigens can be readily expressed as recombinant proteins in *T. thermophila *[46], which can act as a surrogate platform for immobilization assays with reference antisera to determine the serotype specificity of the genes in question.

Figure 4a displays a phylogenetic tree of all 17 predicted i-antigens, showing that most of the newly identified genes cluster separately from the three discussed above. Indeed, some were nearly identical to one another and likely arose through gene duplication (for example, IMG5_190870 and IMG5_190880, IMG5_069230 and IMG5_069240). Most of the genes were shown to be adjacent to at least one other i-antigen gene (Figure 4b), usually in tandem arrays, and indeed, because they were found on relatively short scaffolds that were not placed on the optical map, it is possible that most or all are arranged in even larger clusters or perhaps only a single one. A group of 12 genes encodes predicted proteins of similar size (303 to 340 amino acids) that share common sequence motifs throughout their length. They also lack stretches of amino acids that were present within the second and third repeats of the previously characterized serotype A and D i-antigens (Additional file 10). This pattern of conserved stretches of amino acids within a framework of higher order repeats, along with the genomic arrangement of genes, suggests that the i-antigen genes have evolved through a series of tandem duplications, with intermittent recombination and point mutation giving rise to new variants. Finally, while the predicted products of all 17 genes share common sequence elements, available evidence would suggest that the antigenic determinants associated with protective immunity are conformational and synonymous with those that define serotype [43]. Once these determinants are characterized, it may be possible to design polyvalent or universal vaccines that react across serotypes.

#### Membrane transporters

The free-living ciliates *Tetrahymena *and *Paramecium *depend heavily on membrane transport systems to regulate their complex behaviors and exchange materials with the environment. Indeed, their genomes contain more predicted membrane transporter genes than those of most other sequenced eukaryotes, including animals and plants [9,11]. Recent studies on some of the expanded families of ciliate transporters have begun to shed light on their functional diversification [47,48]. We were interested to see how Ich's adaptation to a parasitic lifestyle has affected its complement of transporter genes.

We detected a total of 483 predicted transporter genes in the Ich genome, 56% fewer than the 1,086 found in *T. thermophila *(a substantially less significant reduction than the 67% in overall predicted proteome size). Transport protein analyses are summarized in Table 4 and fully presented in Additional files 11 and 12, according to the transporter classification (TC) schema of the Transporter Classification Database (TCDB) [49,50], a functional/phylogenetic system of classification. Table 4 shows the breakdown of transport proteins according to TCDB functional class. In *Tetrahymena*, the greatest number fall into class 1, channels, which facilitate transport of molecules down a gradient (in a gated or non-gated manner). In contrast, the largest category of Ich transporters are secondary carriers (class 2), the mechanism of which involves coupling to chemiosmotic energy. Class 3, primary active transporters, which use a primary source of energy such as diphosphate bond hydrolysis to drive active transport, constitute approximately a quarter of both Ich and *Tetrahymena *transport proteins.

**Table 4:** Comparison of Ich and *Tetrahymena *membrane transporters according to TC class, superfamily and substrate type

	Number of transporters in Ich (percentage of total transporters)	Number of transporters in *Tetrahymena *(percentage of total transporters)
TC class		
1. Channels	155 (32)	418 (38)
2. Secondary carriers	191 (40)	386 (36)
3. Primary active transporters	131 (27)	269 (25)
9. Poorly characterized transporters	6 (1)	13 (1)
Total	483	1086
TC superfamily		
VIC	116 (24)	396 (36)
APC	7 (1)	64 (6)
MFS	75 (16)	146 (13)
MC	38 (8)	43 (4)
CPA	16 (3)	20 (2)
ABC	40 (8)	159 (15)
P-ATPase	65 (13)	91 (8)
Substrate type		
Inorganic molecules	249 (51)	500 (53)
Carbon sources	54 (11)	77 (8)
Drugs, toxins, macromolecules	77 (16)	155 (17)
Vitamins, co-factors	15 (3)	23 (2)
Nucleotide bases and derivatives	21 (4)	26 (3)
Amino acids and derivatives	34 (7)	49 (5)
Unknown	38 (8)	110 (12)

MFS, major facilitator superfamily; VIC, voltage-gated ion channel; MC, mitochondrial carrier; CPA, cation:proton antiporter.

Table 4 also summarizes selected phylogenetic superfamily representation. Among the channels, the dominant superfamily is the voltage-gated ion channels (VICs), which exhibit specificity for potassium, sodium or calcium or are cation non-specific. Representatives of these channels in ciliates are involved in ciliary beating, mechanotaxis and other functions [51,52]. The *T. thermophila *genome contains 396 predicted VIC superfamily genes, but Ich has only 116, perhaps reflective of a reduction in its behavioral complexity in adapting to a parasitic life style. However, when examined in greater detail, we found that the extent of this difference between species varied sharply by cation substrate. Ich contains only 22% as many VICs family K^+ ^channels as *Tetrahymena *and 71% as many Na^+ ^channels. Predicted Ca^2+ ^channel genes (1.A.1.11) of both Ich and *Tetrahymena *required manual re-inspection (see Materials and methods), but we conclude that the Ich genome contains between 13 and 19 whereas *Tetrahymena *has no more than 7. Thus, Ca^2+ ^regulation is likely to be of great importance in Ich, although the need for K^+ ^channels is minimal compared to *Tetrahymena*.

Several other genomic lines of evidence suggest a critical role for Ca^2+ ^in Ich. More P-type Ca^2+^-ATPases (TC number 3.A.3.2) were identified in Ich than in *Tetrahymena *(13 versus 11), but fewer K^+^-transporting ATPases. In addition, calcium channels of the ryanodine-inositol 1,4,5-triphosphate receptor Ca^2+ ^channel (RIR-CaC) family (1.A.3) were increased in number in Ich compared to *Tetrahymena *(32 versus 25). Of these, eight and three, respectively, appeared to be incomplete with less than six peaks of hydrophobicity. Thus, maximally 24 and 22 potentially full-length sequences were identified for these two organisms, respectively, but by this calculation, Ich still has more members of family 1.A.3 than does *Tetrahymena*. As described above, Ich and other ciliates also contain a large number of calcium- and calmodulin-regulated protein kinases, including members of the CDPK family, which is absent in vertebrates. Calcium-regulated pathways have come under study as promising therapeutic targets against apicomplexan parasites [42,53] and we propose they also be considered as targets against Ich.

Ich apparently uses primarily secondary carriers (TC class 2) for uptake of nutrients (twice as many as primary active transporters; 114 versus 58) but uses approximately equal numbers of primary and secondary transport systems for drug export. The Ich genome contains only one-quarter as many ABC transporters as that of *Tetrahymena*, but 71% as many P-type ATPases. Of the former, MDR pumps (ABC families 201, 204, and 208) are preferentially reduced in Ich compared to *Tetrahymena *(only 14 to 23% as many; Additional file 12), compared with an equal number (two) of peroxisomal long chain fatty acid transporters (family 203) and 40% as many cholesterol/phospholipid flippases. We also note that P-type ATPase phospholipid flippases are increased in numbers compared to *Tetrahymena *(35 compared to 32; family 3.A.3.8) and that this family is the largest of these enzyme transporters in Ich. Seventy-five transporters of the major facilitator superfamily (MFS) [54] were identified. Compared with *Tetrahymena*, MFS transporters specific for organic cations and sugars were better represented than those specific for organic anions.

Mitochondrial carriers transport all types of small molecules concerned with aerobic metabolism and permit communication between the cytosol and the mitochondrial matrix. There are 88% as many mitochondrial carriers in Ich as in *Tetrahymena *(compared with 44% as many transporters of all types). Such a high representation of these carriers suggests a strong dependence of Ich's energy generation on mitochondrial aerobic respiration. This is potentially significant because, as noted above, Ich mitochondrial ATP synthase is highly divergent from its vertebrate form and thus an attractive drug target.

Most families in the APC superfamily were lacking in both ciliates. Only two of these families, AAAP (2.A.18) and NSS (2.A.22) were represented in Ich. Members of the SSS family (2.A.21; 27 in *Tetrahymena*) were completely absent in Ich. Ich representation was largely restricted to the AAAP (APC) and the OCT (MFS) families, a most unusual representation compared to other characterized organisms.

By percentage of total transport proteins specific to a general substrate type (Table 4), there are no dramatic differences between Ich and the free-living *Tetrahymena*. By far the largest percentage (51%) are devoted to inorganic molecules, particularly small ions. The majority of these proteins are channels and secondary active transporters, but they also include 30 P-type ATPases [55]. Predicted cation transporters greatly outnumber predicted anion transporters (236 to 13), an imbalance observed to an even greater extent in *T. thermophila *(485 to 15) [11].

Transporters specific for lipids comprise nearly 9% of the total. Of these, 35 identified in Ich belong to the inwardly flipping lipid translocating P-type ATPase (TC number 3.A.3.8) family; only 32 of these flippases were identified in *Tetrahymena*. However, only 4 transporters similar to the ABC-porter, 3.A.1.211.5, involved in lipid secretion, were identified in Ich, while 20 were identified in *Tetrahymena*. The transporters involved in protein secretion in Ich are found in the 3.A.5 (general secretory pathway) and 3.A.8 (mitochondrial protein import system) families. The proteins we identified were the integral membrane transporters that form the transmembrane pores. These observations suggest that both of the systems are intact in Ich. The general secretory pathway may be involved in pathogenesis by secreting proteins required for parasite attachment, host tissue digestion and/or immune evasion and thus represent a potential therapeutic target.

A fairly high number (34; 7% of total) of transporters appear to be specific for amino acids and their derivatives, suggesting that these substrates are also of importance for the physiology of Ich; indeed, metabolic reconstruction (see below) shows that Ich is auxotrophic for many amino acids. We were unable to predict a substrate for 8% of Ich transporters.

Proteins in the various families of the TCDB system have been found to have characteristic topological features [56]. Additional file 13 illustrates the distribution of Ich transporter topological types based on numbers of transmembrane segments (TMSs), suggesting that Ich has an unusual distribution of topological types relative to other types of eukaryotes and prokaryotes. The significance of this finding is unclear.

#### Proteases

Proteases in parasitic protozoa have long been considered potential drug targets due to their crucial roles in parasite development and infection, and the feasibility of designing specific inhibitors [57-60]. For example, carp infected with Ich produce elevated levels of a2M3, an isoform of A2M, a non-specific protease inhibitor of endogenous and exogenous proteases [61]. This naturally occurring strategy strongly suggests that anti-proteases could be viable anti-infectives. However, our knowledge of the protease complement in Ich is very limited. To date, only two cathepsin L cysteine proteases (Icp1 and Icp2) belonging to the C1 papain peptidase family have been characterized [62]. Here, comparative genomic analysis reveals that the Ich proteolytic repertoire (degradome) consists of 254 protease homologs, approximately 3.1% of the proteome (Table 5; Additional file 14). This significantly expands the range of protease targets. The fraction of proteases in the Ich genome is close to the average observed in the 1,569 organisms with completed genomes (2.6%) but higher than in the annotated protozoan genomes of *T. thermophila*, *P. tetraurelia*, and *P. falciparum*.

**Table 5:** Protease complements in Ich and other model organisms

	Catalytic class						Percentage of the
			
Organism	Aspartic	Cysteine	Metallo	Serine	Threonine	Total	proteome^a^
*Ichthyophthirius multifiliis*	14 (5.5%)	81 (31.9%)	119 (46.9)	25 (9.9%)	15 (5.9%)	254	3.1
*Paramecium tetraurelia*	48 (8.3%)	225 (38.9%)	168 (29.1%)	95 (16.4%)	42 (7.3%)	578	1.5
*Tetrahymena thermophila^b^*	43 (9.0%)	211 (44.0%)	139 (28.9%)	73 (15.2%)	14 (2.9%)	480	1.7
*Plasmodium falciparum*	17 (10.5%)	33 (34.7%)	21 (22.1%)	16 (16.9%)	15 (15.8%)	95	1.8
*Neurospora crassa*	19 (8.1%)	41 (17.4%)	81 (34.5%)	75 (31.9%)	19 (8.1%)	235	2.4
*Saccharomyces cerevisiae*	19 (11.1%)	41 (24.0%)	57 (33.3%)	38 (22.2%)	16 (9.4%)	171	2.4
*Caenorhabditis elegans*	27 (5.6%)	125 (25.9%)	190 (39.4%)	115 (23.9%)	25 (5.2%)	482	2.4
*Drosophila melanogaster*	46 (6.2%)	86 (11.5%)	207 (27.7%)	373 (49.9%)	35 (4.7%)	747	5.4
*Homo sapiens*	320 (29.3%)	190 (17.4%)	252 (23.0%)	291 (26.6%)	41 (3.7%)	1,094	4.5
*Arabidopsis thaliana*	233 (27.6%)	162 (19.2%)	112 (13.3%)	306 (36.2%)	31(3.7%)	849	3.1

Values in parentheses are the percentage of the individual catalytic class in the protease complement. ^a^The percentage of the whole genome that encodes putative proteases. ^b^The distributions of *T. thermophila *and *P. falciparum *are based on Eisen *et al. *[11] and Wu *et al. *[60], respectively. The distributions of the other model organisms are based on the results published in Merops database.

Using the Merops protease nomenclature, which is based on intrinsic evolutionary and structural relationships [63], the Ich proteases were divided into five catalytic classes and 37 families. These are: 14 aspartic proteases belonging to two families, 81 cysteine proteases belonging to 10 families, 119 metalloproteases belonging to 14 families, 25 serine proteases belonging to 10 families, and 15 threonine proteases belonging to the T1 family (Table 5; Additional file 14).

Comparison with *T. thermophila *(see Table S11 in [11]), and *P. tetraurelia *(data not shown) reveals that Ich possesses a core degradome structure similar to these ciliates (Additional file 15). Thirty-five out of 37 protease families found in Ich are present in all three genomes. Only one protease family, the Xaa-Pro dipeptidyl-peptidase family (S15), is unique to Ich. The S15 homolog is also present in other protozoan parasites, including *Leishmania major *and *Trypanosoma cruzi*, but is not found in *P. falciparum*. A homolog of D-alanyl-glycyl peptidase (C51) is found in Ich and *P. tetraurelia*, but is missing in *T. thermophila*, *P. falciparum *and other completed protozoan genomes. This family of peptidases was found in a bacteriophage that is capable of degrading bacterial cell-wall cross-linking peptides to release phage particles from the bacterial cytoplasm [64]. Its role in protozoa has not been characterized. Seven families of proteases (C15, C48, C50, C56, M15, S9, S33) that are present in the two free-living ciliate genomes are not found in Ich.

Ich possesses a number of protease families that may play important roles in the parasitic life cycle. For example, 14 members of the calpain family (C2) are present in Ich, constituting 5.5% of the degradome, implying a strong calcium-dependent regulatory mechanism that may be involved in signal processing, cell cycle progression or ion channel activities [65]; Signal peptidase I family (S26) may play a role in the secretion system by removing the hydrophobic signal peptides when the precursors are moving across the membrane.

The two largest protease families in Ich are the leishmanolysin (M8) and the ubiquitin carboxyl-terminal hydrolase (C19) families, which contain 54 and 39 members, respectively, representing substantial percentages of the degradome (21.3% and 15.4%; Additional file 15). As discussed in Eisen *et al. *[11], leishmanolysin (M8) was originally identified in the kinetoplastid parasite *L. major *and thought to be involved in processing surface proteins [66,67], but to date the functions of leishmanolysin in nonkinetoplastid eukaryotes remain unclear. The 39 members of the C19 family and 15 members of the threonine proteases (T1) likely arose from large-scale gene duplication events. Such a massive retention of duplicates reflects the crucial role of the ATP-dependent ubiquitin-proteasome system, which has been implicated in cell-cycle control and stress responses [68].

#### Cytoskeletal proteins

Ciliates are characterized by complex cytoskeletal architectures. Microtubule-based structures are highly diverse, with at least 18 types of microtubular organelles having been described in *T. thermophila *[69]. This diversity is reflected at the genome level; in comparison to humans, *T. thermophila *encodes a greater number and/or variety of several classes of cytoskeletal protein, including tubulins, microtubule motors and microtubule regulatory enzymes [11].

Although Ich has a reduced genome size compared to *T. thermophila*, it also has an elaborate cytoskeleton and undergoes dramatic changes in cell morphology during its life cycle. We found that although the number and/or diversity of certain cytoskeletal protein genes was reduced relative to *T. thermophila*, others, such as kinesins and tubulin tyrosine ligases, remained expanded, even in comparison to humans (Table 6).

**Table 6:** Cytoskeletal proteins in Ich, *T.thermophila *and *H. sapiens*

Protein type	Ich	*T. thermophila*	*H. sapiens *
**Tubulins and modifying enzymes**			
α-Tubulin	1	1	9
α-Tubulin-like	0	3	0
β-Tubulin	3	2	9
β-Tubulin-like	0	6	0
γ-Tubulin	1	1	2
ε-Tubulin	1	1	1
δ-Tubulin	0	1	1
η-Tubulin	1	1	0
ι-Tubulin	0	3	0
Tubulin tyrosine ligase-like	31	50	14
**Motor proteins**			
Kinesin motor domain	41	78	48
Dynein heavy chain	19	25	16
Dynein intermediate chain	6	6	7
Dynein light intermediate chain	1	2	3
Dynein light chain	16	14	9
Myosin motor domain	3	13	22
**Centrins**			
Centrin 1	1	1	1
Centrin 2	2	1	1
Centrin 3	1	1	1
Centrin 4	1	1	0
**Core basal body proteins (also includes centrins 2, 3; δ-, ε-tubulins)**			
Bld10/Cep135	1	1	1
Centriolin	0	1	1
Cep76	0	0	1
Cep164	0	1	1
Dip13	0	0	2
Poc1	1	1	2
Poc5	1	1	1
Sas4/CPAP	2	1	2
Sas6	1	2	1
VFL1a/CLERC	1	2	1
WDR16	1	1	1
**Ciliopathy associated proteins**			
MKS1	1	1	1
MKS3	2	2	1
MKS4/Cep290	0	0	1
MKS5/RPGRIP1L	1	1	1
MKS6/CCD2A	1	1	1
AHI1	1	1	1
NPHP1	1	1	1
NPHP3	0	0	1
NPHP4	1	1	1
BBS1	1	1	2
BBS2, 5, 7, 8, or 9	1	1	1
BBS3/ARL6	0	1	1
BBS4	0	1	1

Several tubulin isoforms found in *T. thermophila *and *P. tetraurelia *were absent from Ich. *T. thermophila *encodes three alpha tubulin-like and six beta tubulin-like proteins. The functions of these isoforms, which lack motifs for post-translational modifications that are essential to the function of their canonical counterparts, are not clear, but none of them is detectable in the Ich genome. In addition, although Ich encodes the variant gamma, epsilon and eta tubulins, the functions of which are thought to include basal body duplication [70], it lacks delta and iota. Delta tubulin is involved in assembly of the triplet microtubule structure found in most centrioles and basal bodies [70,71], suggesting that the molecular mechanisms of centriole assembly may be divergent between Ich and *Tetrahymena*.

A highly conserved class of microtubule organizing center-associated proteins are the centrins [72], composed of four EF-hand motifs that are regulated by calcium. The centrin families of Ich and *Tetrahymena *are generally comparable (Table 6), with the exception that two *Cen2 *genes are present in Ich compared to one in *Tetrahymena*. A collection of 14 (grouping Cen2 and Cen3; Table 6) highly conserved core proteins involved in centriole and basal body biogenesis and function was recently described [73,74]. Of these, the *Tetrahymena *genome contains twelve but Ich only nine. Three of the proteins contained in both Ich and *Tetrahymena *(Cep135/Bld10, SAS6, and SAS4/CPAP) are members of an ancestral module (UNIMOD) correlated with the presence of basal bodies and centrioles [73,74]. However, the Ich basal body appears to be simplified compared to *Tetrahymena*, with no centriolin, Cep164, Dip13 or δ-tubulin, and single Vfl1a/CLERC, WDR16 and SAS6 genes compared to two each in the *Tetrahymena *genome.

Ciliopathies are a class of human disease associated with defects in basal bodies and cilia. Many of the proteins defective in ciliopathies are broadly conserved [74]. We found many of the ciliopathy genes in the genomes of both *Tetrahymena *and Ich (Table 6). MKS3, associated with Meckel-Gruber syndrome, is expanded in both genomes with two versions of this gene. In contrast, MKS4/Cep290 and NPHP3 are not present in either ciliate. Finally, BBS3/ARL6 and BBS4 are found in *Tetrahymena *but not Ich. Because BBS3/ARL6 is a member of the large Ras GTPase family, it may have escaped detection. Alternatively, a different Ras family member may function in its place. Unlike BBS3, BBS4 is a member of the BBSome, a conserved complex involved in ciliary membrane transport. Because BBSome members tend to evolve together as a module [74], we were surprised that BBS4 was not identified in the Ich genome. BBS4 interacts with the centrosome component, PCM1, and is implicated in both centrosome organization and transport of the BBSome to cilia [75,76]. Perhaps these functions are not necessary in Ich, or the gene may be found in an unassembled region of the genome.

Dyneins are microtubule-based motors that perform a variety of essential functions in eukaryotic cells [77]. Multiple dyneins are present in cells with cilia or flagella, each specialized in its location and function [78]. There are seven classes of dyneins: (i) conventional cytoplasmic dynein-1, important for karyokinesis and intracellular membrane organization and trafficking; (ii) cytoplasmic dynein-2, which participates in retrograde intraflagellar transport; (iii) axonemal inner arm dynein I1 (IAD-I1), which generates shear between the ciliary outer doublet microtubules; (iv) axonemal outer arm dynein (OAD), which accelerates outer doublet sliding; and (v to vii) axonemal single-headed inner arm dynein (sh-IAD) groups 3, 4, and 5, which contribute to outer doublet sliding [79]. Each dynein is composed of one or more heavy chains and a set of intermediate, light intermediate, and light chains. The heavy chains contain the motor activity [80-82]. The smaller components are important for the regulation and location of dynein activity [83].

In the Ich genome, we identified genes encoding 19 heavy chains, six intermediate chains, one light intermediate chain, and 16 light chains (Table 6). The dynein genes of Ich are most similar to those of *T. thermophila *[11,79,84]. Neither *T. thermophila *nor Ich has genes encoding light chains LC3, LC5, or LC6, or intermediate chain IC1, which are found in other organisms. The Ich dynein genes differ from those found in *Tetrahymena *in several respects. Firstly, we did not find a dynein-2 light intermediate chain (D2LIC), suggesting that the retrograde intraflagellar transport motor dynein-2 may be inefficient. A pseudogene of D2LIC is present in the Ich genome, suggesting that expression of this gene has been lost. In *Tetrahymena*, deletion of D2LIC affects regulation of ciliary length [85]. Secondly, the Ich ciliary outer arm dynein complex may be different from the OADs found in other protozoa. Metazoans have a two-headed OAD composed of the heavy chains α and β [86]. In addition, all protozoa examined express a third heavy chain related to the β gene; we refer to these two related genes as β/γ. However, Ich appears to lack a second β/γ gene. Additionally, we did not find the highly conserved OAD light chain LC10. Loss of LC10 in *Chlamydomonas *results in only a subtle reduction in flagellar beat frequency, but lack of both LC10 and LC2 has a more severe effect on beat frequency than the lack of either individually [87]. Finally, the Ich single-headed inner arm dyneins are relatively less diverse than in *Tetrahymena*. For example, while nearly every sh-IAD of Ich has a clear *Tetrahymena *ortholog, there are several additional *Tetrahymena *sh-IADs not paired with Ich genes (for example, *Tetrahymena *DYH10, DYH19, DYH20, and DYH25), suggesting expansion of the *Tetrahymena *sh-IAD gene family or loss of Ich genes after the two species diverged.

#### Analysis of metabolic pathways

Many antibiotics target metabolic pathways present in infectious agents but not their hosts [88]. To identify candidate drug targets in Ich, we mapped Ich enzymes onto Kyoto Encyclopedia of Genes and Genomes (KEGG) metabolic pathways [89] and compared them with a well-annotated fish genome, that of the zebrafish *Danio rerio*, as well as those of the free-living ciliates *T. thermophila *and *P. tetraurelia *(Additional file 16). The overall metabolism of Ich is very similar to that of the free-living ciliates, but with some minor interesting differences. In contrast, significant differences were found between Ich and fish.

All pathways constituting central carbon metabolism, such as glycolysis, the citric acid cycle and the pentose phosphate pathway, are present in all three ciliates. However, all three appear to be missing the first two enzymes of the pentose phosphate pathway - glucose-6-phosphate dehydrogenase and 6-phosphogluconolactonase - which convert glucose-6-P to 6-P-gluconate and in the process generate NADPH, H^+^. These enzymes are important contributors to the maintainance of cytosolic NADP+/NADPH, H+ balance. It is unclear what other mechanism is in place to maintain this balance in ciliates.

It appears from metabolic reconstruction that another major difference between fish and ciliates is that, while fish store carbohydrates as glycogen, ciliates cannot make glycogen but instead make starch or amylopectin. However, in light of reports on glycogen metabolism in *Tetrahymena *in the biochemical literature [90], the basis of carbon storage in ciliates requires further confirmation.

Mitochondrial metabolism in Ich and other ciliates is comparable to other eukaryotes. Ich possesses the enzymes of various oxidative pathways, including pyruvate dehydrogenase, the citric acid cycle and β-oxidation. Ich can also channel reducing equivalents (NADH, FADH_2_) generated by these oxidative pathways for ATP synthesis via oxidative phosphorylation. All alveolate organisms sequenced to date, including Ich, harbor an atypical eukaryotic mitochondrial F_1_-F_0_-ATP synthase (see above under discussion of mitochondrial genome). The ciliates also possess all the enzymes that participate in the glyoxalate cycle (isocitrate lyase, malate synthase) and 2-methylcitric acid cyle (2-methylcitrate synthase, 2-methylcitrate dehydratase, methylisocitrate lyase), which are absent in fish. The glyoxalate pathway breaks down isocitrate (a citric acid cycle intermediate) directly into succinate and malate. This bypass pathway helps conserve carbon by avoiding its release as CO_2_, which would occur during a full progression of the citric acid cycle. Similarly, the 2-methylcitric acid cycle is required to detoxify propionyl Co-A (obtained from β-oxidation and branched chain amino acid oxidation), a task that is achieved in fish by the methyl malonyl-CoA pathway. Both these pathways allow the ciliates to convert fatty acid oxidation products into carbohydrates, a process that is known to happen in many bacteria and plants but not in animals.

Fatty acid metabolism is very reduced in all three cilitates as they lack almost all enzymes that participate in traditional FAS-I and FAS-II fatty acid synthesis. However, they seem to have the complete set of enzymes required for fatty acid elongation and metabolism via β-oxidation. Also, Ich cannot synthesize steroids such as cholesterol but seems to be capable of modifying them - for example, cholesterol can be converted into cholesteryl esters. One very striking metabolic feature of Ich and other ciliates is that they are deficient in the *de novo *biosynthesis of both purines and pyrimidines and thus appear to be solely reliant on salvage pathways for sustaining nucleotide metabolism. These pathways are attractive candidates for drug intervention [88]. The ciliates have a battery of purine and pyrimidine salvage enzymes but are also missing some - for example, they cannot interconvert IMP and GMP as they lack both GMP synthase and GMP reductase, requiring them to salvage precursors for both GMP and AMP, as they cannot get one from the other. In similar fashion, Ich and other ciliates depend on pyrimidine salvage enzymes such as uridine kinase and cytosine deaminase. Cytosine deaminase is absent in fish.

Amino acid metabolism in Ich is minimal and it appears to be auxotrophic for many varieties. However, unlike fish and other ciliates, Ich encodes the enzyme cysteine synthase, which can use H_2_S as a sulfur donor to synthesize cysteine. The metabolism of glutamine, glutamate, aspartate and alanine is very similar to that in fish and other ciliates. Although Ich and other ciliates cannot synthesize phenylalanine or tyrosine *de novo*, they still harbor the shikimic acid pathway and have the pentafunctional AROM polypeptide. This pathway is required for chorismate/folate biosynthesis. It is absent from fish and has been studied as a drug target in other systems. The enzyme used by Ich and other ciliates to make selenocysteine (cysteine synthase A) is different from that used by fish (selenocysteine lyase).

While glutathione formation and it roles in oxidation/reduction metabolism is the same between Ich and fish, Ich seems to also possess enzymes necessary for trypanothione synthesis and its use in oxidation/reduction metabolism. If verified, this pathway could be an excellent candidate for drug development.

## Conclusions

Because of its evolutionary proximity to the well-studied, free-living *T. thermophila *and *P. tetraurelia*, Ich's genome sequence provides an interesting comparative viewpoint on the consequences of adaptation to a parasitic lifestyle. Ich has experienced a dramatic reduction in macronuclear gene content, but at the same time retains remarkable diversity of many gene families, such as kinases and membrane transporters, even in comparison to complex metazoan organisms. Ich's basic metabolic and cellular functions appear largely intact relative to its free-living relatives, but unlike *Tetrahymena *and *Paramecium*, Ich contains far fewer lineage-specific ortholog groups, in particular those presumed to be involved in signaling pathways and gene regulation. This suggests a genomic consequence of Ich's dependence on a host has been a reduction in the capacity for behavioral and regulatory complexity characteristic of predatory ciliates.

The full catalog of immobilization antigens for this strain, as well as candidates for other surface proteins, will facilitate elucidation of the mechanisms of antigenic variation and the development of more effective vaccines to prevent white spot disease. Likewise, the comparative genomics and comprehensive metabolic reconstruction made possible by the genome sequence provide numerous candidates for effective therapeutic intervention. Strikingly, several of these candidates are also being investigated as potential drug targets against other parasites, such as apicomplexans. These include the highly divergent ATP synthase, purine and pyrimidine salvage enzymes and calcium-based regulatory pathways. Thus, the fight against white spot disease may well benefit from research directed against malaria and other human diseases. To facilitate their use by the research community, the Ich genome sequence and annotation have been loaded into the genome browser of the *Tetrahymena *Genome Database [91].

## Materials and methods

### Animal care

Because *I. multifiliis *is an obligate parasite, the collection of sufficient biological material to allow genomic and transcriptomic sequencing required cultivation of the parasite on live fish. All experiments were carried out in strict accordance with the recommendations of the Guide for the Care and Use of Laboratory Animals of the National Institutes of Health so as to minimize pain and suffering. The protocol was approved by the Institutional Animal Care and Use Committee of Cornell University (protocol number 1996-0083). Fish were anesthetized with tricaine methane sulfonate (MS-222) when handled for parasite collection in order to reduce stress.

### Strain origin and propagation

*I. multifiliis *(isolate G5; serotype D) was isolated from an albino channel catfish (*Ictalurus punctatus*) in 1995 and propagated by passage on juvenile channel catfish as previously described [92]. In 2004, a cloned line of the G5 isolate was derived from a single tomont by hand-pipetting individual tomonts into wells of a 96-well microtiter plate. Tomonts hatched overnight at room temperature. Theronts from a single well were then used to infect a channel catfish and progeny from that infection were subsequently maintained by serial passage on fish [92]. Strain G5 became senescent and was lost in 2009.

### Genomic DNA isolation

Tomonts were isolated from infected channel catfish as previously described and individually collected by hand-pipetting [17]. DNA was isolated from batches of 200 to 500 cells, either directly from tomonts, or from MAC fragments obtained from cell lysates. To lyse cells, tomonts were homogenized using a pestle for a 1.5 ml microcentrifuge tube in 0.2 ml of lysis buffer (10 mM Tris, 3 mM CaCl_2_, 1 mM MgCl_2_, 0.25 M sucrose, 0.5% NP40, 0.5% Tween-20, pH 7.9). An additional 1 ml of lysis buffer was added to the lysate and MAC fragments collected by centrifugation in a microcentrifuge tube at 1,000 × g for 10 minutes at 4°C. DNA was prepared from tomonts or the MAC pellet, as previously described [17], treated with 40 μg/ml RNAse A/T1 (Fermentas; Glen Burnie MD, USA) for 2 hours at 37°C, precipitated with ethanol and resuspended in 10 mM Tris, 1 mM EDTA, pH 8.0.

### Genome sequencing and assembly

Plasmid libraries were constructed and end-sequenced at the J Craig Venter Institute, as previously described [11], producing a total of 297,031 high quality reads [93]. In addition, four and a half 454 FLX Titanium runs were performed, resulting in 3,167,209 good reads (SRX036996, SRX036983, SRX036682, SRX036681, SRX036678). All reads were assembled using Celera Assembler version 5.3 [94], setting error rate to 8% and the utgGenomeSize to 200 Mb. Following initial assembly, the reads that comprised scaffolds having a GC content of less than 26% were reassembled with Celera v 5.3. A total of 216,200 Sanger reads and 2,008,917 454 reads contributed to the Ich assembly, yielding 2,342 contigs in 1,803 scaffolds with a contig N50 of 51,903 bp. Unfortunately, because of the presence of symbiont reads, the number of unassembled Ich reads cannot be accurately determined. Of the 540 intra-scaffold gaps, 455 were successfully targeted by an automated primer design program [95] modified from the original version to iteratively expand the target amplicon size, instead of a fixed tiling. Sanger clones spanning gaps were selected for primer walks, which produced 1,406 good reads. Celera Assembler was run on the combined Sanger shotgun, 454 shotgun, and Sanger finishing reads dataset. The final assembly produced 2,274 contigs (accession numbers AEDN01000001 to AEDN01002274) in 2,015 scaffolds (accession numbers GL983039 to GL985055) with a contig N50 of 55,110 bp and average depth of 19X.

The ribosomal RNA locus, found on an amplified palindromic chromosome, was present as a truncated 7 kb contig in the initial assembly, based on alignment to published 18S and 28S sequences. The complete rDNA chromosome was assembled by recruiting additional Sanger mates to the existing contig using the J Craig Venter Institute sequence editor Cloe, up to the palindromic center of the chromosome (accession ID GL985055).

The Ich mitochondrial genome was not present in the initial assembly, likely due to high coverage. To detect it, degenerate and singleton reads were assembled with Celera Assembler, and contigs over 2 kb were BLASTed against the NCBI non-redundant nucleotide database, resulting in the identification of one 12 kb contig with similarity to the mitochondrial genome of *Tetrahymena malaccensis*. All Sanger reads were aligned to this seed 12 kb contig with Nucmer. Reads aligning with over 97.5% identity were combined with their mates and assembled using TigrAssembler [96], producing an extended contig. This process was iterated until a telomeric tandem repeat was reached on one side and a gap on the other. Overlapping 454 reads were used to extend through the gap, and the alignment of Sanger reads and reassembly was again repeated until the other telomere was reached. The final edited contig qualifies as finished with two small areas of quality exception that contain 454 reads and low quality Sanger reads (accession ID JN227086).

### Optical map generation and analysis

High molecular weight Ich DNA was prepared directly from isolated trophont stage cells by a modified pulsed-field gel electrophoresis method [97]. Optical maps were prepared by OpGen, Inc. (Gaithersburg MD, USA) as previously described [98]. In brief, single DNA molecules were captured onto a microfluidics optical chip, subjected to *in situ *digestion with *Spe*I restriction endonuclease (New England Biolabs; Ipswich MA, USA) and analyzed by automated fluorescence microscopy to generate single molecule maps. *Spe*I was selected because it cuts on average about every 10 kb in the Ich genome. Collections of single molecule maps were then assembled by the Gentig program by their overlapping restriction fragment patterns to produce whole-genome ordered restriction maps, or optical maps, of 69 complete chromosomes, four partial chromosomes and a single 1.6 Mb bacterial symbiont chromosome. Electronic *Spe*I digests were produced for all eukaryotic scaffolds, resulting in 732 scaffolds with more than one cut each. SOMA [28] was used to align the scaffolds to the optical map, using a three-tiered algorithm. The highest confidence alignment algorithm, MATCH, uniquely mapped 337 scaffolds. This was followed by the FILTER algorithm, which uses heuristic filtering to exclude the scaffolds already placed, resulting in 30 additional mapped scaffolds. The final (less reliable) algorithm, SCHEDULE, mapped 188 additional scaffolds, a total of 555 scaffolds containing 36.1 Mbp. MapSolver placed 319 scaffolds containing 27.2 Mbp. Telomere-containing scaffolds were found by searching for three tandem copies of the sequence GGGGTT, identifying 121 scaffolds, all of which ended in the repeats in their proper orientation. Applying the criteria described in the Results and discussion section, we considered 295 scaffolds to be reliably placed, including 56 that contain telomeric repeats.

### EST sequencing and alignment to the genome

Packed cell pellets (10 to 200 μl) were resuspended in approximately 0.5 ml sterile carbon-filtered H_2_O and 8 volumes of Trizol reagent (Invitrogen, Carlsbad, CA, USA) were added. Total RNA was extracted following the manufacturer's instructions. Equal amounts of total RNA from theront and trophont stages were pooled. PolyA+ RNA was selected and normalized by Evrogen, Inc. (Moscow, Russia). The normalized cDNA population was sequenced using the Illumina platform, generating 100 bp paired-end reads. A total of 1.65 × 10^7 ^good reads were obtained, for a total of 1.67 Gb of raw RNA-seq data (SRX048641). These reads were aligned to the genome sequence and assembled using the TopHat suite (TopHat, Bowtie and Cufflinks) [99,100]. Alignments were further refined using PASA [101]. Of 24,264 assemblies input into PASA, 24,078 (99.2%) produced valid alignments (95% identity to genome sequence over 90% of length) and 23,585 subclusters. In addition, 32,606 Sanger ESTs identified as being derived from Ich were downloaded from NCBI and aligned to the genome using PASA. Of these, 22,483 produced valid alignments. Many of the non-aligned ESTs matched genes of fish or bacterial origin, suggesting that they are contaminants (see [4] for discussion). Assembly of the valid ESTs produced 4,751 subclusters.

### Protein-coding gene finding

To train gene finding algorithms, a set of 1,044 gene structures was modeled manually using the Sanger and Illumina EST alignments and homology to predicted genes of other species, especially other ciliates. This set was used to train three *ab initio *gene prediction programs: Augustus [102], GeneZilla and GlimmerHMM [103]. An initial full set of gene predictions was generated based on the three *ab initio *algorithms, Ich ESTs, and protein homologies to *T. thermophila*, *P. tetraurelia*, *Oxytricha trifallax *[104] and a J Craig Venter Institute non-redundant protein database, aligned using the AAT [105] and GeneWise [106] programs. Pfam [107] domains were also searched against the genomic sequence. Evidence from the gene finders, protein and domain homology searches and ESTs were used to refine gene models using EvidenceModeler [108]. High quality EST alignments were used to automatically update gene structure annotations using PASA (stringent condition). After extensive manual annotation of selected genes, a total of 8,096 gene models were generated.

### Automated functional annotation

Gene names were computationally assigned by searching protein databases, including the J Craig Venter Institute Panda comparative database, Panther [109], Pfam and Uniprot [110], using BlastP [111]. A subset of the results was manually reviewed to determine cutoffs that produced reasonable names from each of the databases. A subset of gene models was analyzed for correctness and sensitivity to functional assignments. Paralogous families were computed based upon shared domain composition [101]. A minimum of three paralogs were required to designate a 'family'. Multivariate analysis of codon usage was performed using the codonW package [112] as previously described [11].

### Non-coding RNAs

Transfer RNAs were detected using tRNAscan-SE with default parameters [113]. Mitochondrial tRNAs were detected with the same program, set to general (not organellar) mode. 5S rRNAs and other ncRNAs were identified by BlastN search of the Ich genome with *T. thermophila *genes as query sequences.

### Manual curation of selected families

#### Immobilization antigens

The i-antigens were predicted, analyzed and curated manually. The sequences of 12 i-antigens from the genus *Ichthyophthirius *were aligned (ClustalW) [114] and the alignment manually adjusted. The aligned regions were used to build two hidden Markov models using the HMMer programs hmmbuild and hmmcalibrate [115]. These hidden Markov models were searched against the proteome to identify one known G5 input sequence and nine novel i-antigen sequences, some of which were not full length. These additional sequences were added to the set described above and used to rebuild HMMs for a final search.

#### Protein kinases

Two methods were used to identify protein kinase genes. First, 440 genes were annotated with the protein kinase-specific Pfam domain PF00069, all of which grouped with orthologs from other species; 402 mapped to 105 existing ortholog groups and the remaining 36 to previously ungrouped genes (35 from *T. thermophila *and one from *Entamoeba invadens*). Secondly, the Ich kinome was annotated and grouped into kinase families based on orthology to highly curated kinase genes from *T. thermophila*, *Saccharomyces cerevisiae*, *Caenorhabditis elegans *and *Homo sapiens*. The data for these organisms were obtained from the kinbase database [116]. A total of 633 Ich genes were annotated as members of various kinase families in this manner; of these, 402 were already qualified as protein kinases based on Pfam domain annotation while the remaining 231 had orthology assignments only (most of these were either atypical histidine kinases or the ciliate specific kinase families; Additional file 9). Thirty-eight Ich kinases were annotated by Pfam domain information but did not have detectable orthology to any previously known kinase families from either ciliates or other organisms. After combining the results obtained from these two methods a total of 671 Ich genes were annotated as kinases, 602 of which can be grouped into 145 ortholog groups. For comparative purposes, we also retrieved the previously published kinomes of *P. falciparum *[117] and *T. gondii *[118] and constructed preliminary kinomes for *P. tetraurelia *and *D. rerio *based on orthology to the *T. thermophila *and human kinomes, respectively, obtained from kinbase.

#### Transporters

An in-house program called Gblast was used to blast the Ich and *T. thermophila *proteomes against the entire TCDB [119]. Results were tabulated into an excel file that showed each query protein from the Ich proteome with the top hit from TCDB. Careful examination of the 25 putative Ich Ca^2+ ^channels revealed that three of these contain only two TMSs plus the P-loop, four possess one full six TMS repeat unit, seven have two such repeat units, four exhibit three repeat units and four have all four expected repeat units. In addition, three of these sequences were clearly partial sequences with one, two or three repeat units plus a fragmented repeat unit. Further, three sequences were identified that consisted of partial voltage sensors while one more consisted of a partial channel. It seems clear that several incomplete sequences are present within this group of proteins. Thus, the number of Ca^2+ ^channels in family 1.A.1.11 was overestimated by the Gblast program, probably because of inaccurate exon identification in the proteome. We estimate that there are between 13 and 19 Ich Ca^2+ ^channels of family 1.A.1.11. A corresponding examination of the 12 putative *Tetrahymena *Ca^2+ ^channels of family 1.A.1.11 revealed a similar situation where several of these sequences are incomplete. Query TMSs were obtained using the WHAT program [56], which predicts hydrophobicity and amphipathicity along the length of the protein using a window of 19 residues. All information regarding the TC hit proteins was obtained from TCDB. Information relevant to the Ich proteins was extrapolated from TCDB.

#### Proteases

Over 177,390 sequences of characterized and predicted proteases were obtained from the Merops database [120] (release 9.2) [63] and searched against the Ich predicted protein sequences using BLASTP with default settings and an e-value cutoff of less than e^-10 ^for defining protease homologs. Partial sequences (less than 80% of full length) and redundant sequences were excluded. The domain/motif organization of predicted Ich proteases was revealed by a pfam search. For each putative protease, the known protease sequence or domain with the highest similarity was used as a reference for annotation; the catalytic type and protease family were predicted in accordance with the classification in Merops, and the enzyme was named in accordance with SWISS-PROT enzyme nomenclature [121] and the literature.

#### Cytoskeletal proteins

*T. thermophila *homologs were identified previously [11] or by using reciprocal best-hit BLAST strategies. For those components that were found in the *T. thermophila *genome, a reciprocal best-hit BLAST strategy was then used to identify the Ich homologs. Genes were defined as not present in the Ich genome if either a gene family member was identified with a better reciprocal BLAST score to a different family member or a reciprocal BLAST score of better than e^-5 ^was not identified.

#### Dyneins

BLASTP [111] was used to search the predicted Ich proteome. For some genes, TBLASTN was used to search the assembly. Dynein light, light intermediate, and intermediate chain sequences from *Chlamydomonas reinhardtii *or other species as appropriate were used as queries. *T. thermophila *dynein heavy chain 4 (OADβ) was used as query for the heavy chains. Authenticity of candidate sequences was verified by reciprocal best hit blast analysis. Ich sequences were compared to several known dyneins of each type by ClustalW alignment [122]. Evolutionary analyses were performed using MEGA version 4 [19]. Trees were constructed by neighbor-joining [123] and maximum parsimony [124] with 500 bootstraps; both types of tree yielded similar results.

#### ATP synthase

The Ich MAC and mtDNA gene product sequences were searched for sequences closely similar to those reported for the *T. thermophila *ATP synthase [33], and the resulting candidates were compared using BLASTP. In some cases, the Ich gene models were manually corrected using existing EST data and homology considerations.

#### Analysis of metabolic pathways

To map metabolic pathways in Ich, EC numbers were assigned using two different approaches. First, the Ich proteome was submitted to KEGG for automated assignment, identifying 1,789 enzymes but with only 440 unique EC numbers (403 with 4 digits). Second, the Ich proteome was submitted to the OrthoMCL database. We had previously mapped EC numbers obtained for 23 different species from KEGG into orthoMCL groupings, allowing transitive assignment of EC numbers to Ich genes based on their grouping with these enzymes. This method identified 2,307 enzymes with 725 unique EC numbers (649 with 4 digits). We found nearly complete overlap between the results obtained from the two approaches, and after combining had a total of 728 unique EC numbers (651 with 4 digits; Additional file 7). These 651 EC numbers were used to 'paint' the KEGG metabolic pathway maps using KEGG online tools [125]. The Ich enzymes were also painted on existing metabolic pathway maps for *T. thermophila*, *P. tetraurelia *and *D. rerio *for comparative analyses.

## Abbreviations

bp: base pair; CAMK: Ca2+/calmodulin-dependent protein kinase; CDPK: calcium-dependent protein kinase; D2LIC: dynein-2 light intermediate chain; EST: expressed sequence tag; GPI, glycosylphosphatidylinositol; HGT: horizontal gene transfer; Ich: *Ichthyophthirius multifiliis*; KEGG: Kyoto Encyclopedia of Genes and Genomes; MAC: macronucleus/macronuclear; Mb: megabase pair; MIC: micronucleus/micronuclear; mtDNA: mitochondrial DNA; ncRNA: non-coding RNA; OAD: outer arm dynein; ORF: open reading frame; rDNA: ribosomal RNA-encoding DNA locus; sh-IAD: single-headed inner arm dynein; TC: transporter classification; TCDB: Transporter Classification Database; TMS: trans-membrane segment; VIC: voltage-gated ion channel; WGD: whole genome duplication.

## Competing interests

TGC and DCH are founding members of Tetragenetics, Inc., Ithaca, NY, and TGC is a member of its scientific advisory board. The goal of Tetragenetics is to use *T. thermophila *as a platform for the manufacture of biotechnological products, including, potentially, vaccines against *I. multifiliis*. Neither this connection, nor any financial, personal or professional interest of any of the authors, has influenced this paper.

## Authors' contributions

RSC, DMC and TGC conceived and oversaw the project. LH performed whole genome annotation and other informatic analyses. DS and DSR performed orthology, kinome and metabolic analyses. JBH, DB, JJ and DR performed genome assembly, closure and optical map analysis. VSJ performed mitochondrial genome annotation. IS assisted with immobilization antigen and transposon analysis. UK and MS analyzed membrane transporter genes. YF, HC and JG analyzed protease genes. MWM and ABV analyzed ATP synthase genes and assisted in mitochondrial annotation. DEW, VR and DJA analyzed dynein genes. CGP analyzed basal body and cytoskeletal genes. RCF and HWD provided genomic DNA. DMC provided RNA. JB ran and analyzed APIS phylogenetic analysis. MW analyzed codon usage. CM and YVP analyzed gene duplication patterns. RSC and all authors wrote the manuscript. All authors read and approved the final manuscript for publication.

## Supplementary Material

Additional file 1 **Table S1 - additional assembly statistics**. Click here for file

Additional file 2 **Figure S1 - mean scaffold coverage depth**. Mean coverage depth is plotted against scaffold length, showing that, for larger scaffolds, coverage does not diverge greatly from the mean. Click here for file

Additional file 3 **Table S2**. **(a) **Optical map results. Column B lists the scaffold IDs for the 295 scaffolds mapped to the 69 complete and four partial optical chromosome maps (listed in column A from largest to smallest, with the four partial chromosomes at the end). No scaffolds aligned reliably to chromosomes 53, 55, 65 and 66. Column C indicates the orientation of the scaffold sequence relative to the optical map, either end to beginning (EB) or vice versa. 'Chromosome Start' and 'Chromosome End' are calculated from the optical map data and correspond to the positions where each scaffold reliably aligns. 'Scaffold Start' and 'Scaffold End' indicate the portion of the predicted *Spe*I digest of each scaffold that aligns to the map. All lengths are in base pairs. Among the telomere-containing scaffolds, it is evident that the chromosome and scaffold values are not always in exact agreement with their chromosome-terminal positions due to experimental uncertainty in the optical mapping protocol. In the 18 cases (highlighted in yellow) where SOMA but not MapSolver placed a telomere-containing scaffold, the 'Chromosome Start' and 'Chromosome End' values are simply calculated from the total chromosome length and the length of the scaffold. In total, 242 scaffolds were placed by agreement between MapSolver and SOMA with no input from telomere data; 231 of these were placed by SOMA using the highest confidence MATCH algorithm, 9 using the FILTER algorithm and 2 using the SCHEDULE algorithm (see Materials and methods). Thirty-four scaffolds were placed by agreement between MapSolver, SOMA (33 MATCH, 1 SCHEDULE) and telomere position. Eighteen were placed by agreement between SOMA (9 MATCH, 7 SCHEDULE, 2 FILTER) and telomere position. One was placed on partial chromosome 73 by agreement between MapSolver, SOMA and telomere position, although the optical map position is non-terminal, presumably due to a misassembly (see Results and discussion). **(b) **Unmapped telomeric scaffolds. IDs of the 65 telomere-containing scaffolds that did not reliably align to a unique position on the optical map. Click here for file

Additional file 4 **Table S3 - correspondence of predicted genes for ATP synthase subunits of *T. thermophila *and Ich**. Click here for file

Additional file 5 **Table S4 - non-coding RNAs in the Ich genome**. Click here for file

Additional file 6 **Figure S2 - codon usage**. **(a) **Principal component analysis of relative synonymous codon usage in Ich. **(b) **Effective number of codons (ENc; a measure of overall codon bias) for each predicted ORF is plotted versus GC3 (the fraction of codons that are synonymous at the third codon position that have either a guanine or a cytosine at that position). The upper limit of expected bias based on GC3 alone is represented by the red curve. Click here for file

Additional file 7 **Table S5 - mapping of Ich predicted proteins to ortholog groups, phylogeny, kinome annotation and enzyme annotation**. Click here for file

Additional file 8 **Table S6 - ortholog grouping of the predicted proteomes of ciliates**. A listing of all unique ortholog groups mapped to Ich, *T. thermophila *and *P. tetraurelia *protein coding genes. The total number of genes mapped to each ortholog group for each species is indicated, allowing expansions to be identified. The phyletic profile of the mapped ortholog groups is given in the last column. Click here for file

Additional file 9 **Table S7 - comparison of kinase families in Ich and selected other species**. Comparison of all identifiable kinase families from Ich with other species. The numbers indicate the total number of kinase genes from each species for individual families of kinases. Colors are used to highlight kinase families that are present in all three ciliates (yellow), missing in Ich but present in other two ciliates (light blue), and shared between ciliates and apicomplexa only (green). The atypical histidine kinase family, which is greatly expanded in ciliates, is highlighted in pink. The kinase families that are expanded and have at least ten genes in Ich are indicated with red fonts. Click here for file

Additional file 10 **Figure S3 - multiple sequence alignment of Ich immobilization antigen peptide sequences**. Alignment was generated using MUSCLE [126] and edited by hand. Conserved cysteine residues are enclosed in red rectangles. Hydrophobic regions at the amino and hydroxyl termini are shown with yellow highlighting. Click here for file

Additional file 11 **Table S8 - membrane transporter analysis**. Proteins are tabulated according to TC number within the Transporter Classification Database (TCDB) [49,50]. Columns G and H present the query and hit topologies expressed in number of TMSs. Click here for file

Additional file 12 **Table S9 - membrane transporter family distribution**. Click here for file

Additional file 13 **Figure S4 - membrane transporter topological distribution**. The number of proteins exhibiting a specific topological type - that is, of a putative number of TMSs - is plotted versus the number of predicted proteins of that topology, showing that proteins with one, two or three putative TMSs are substantially less numerous than those with four or six putative TMSs. Proteins with 9 or 10 predicted TMSs are present in much lower numbers, but there are increased numbers with 11 and 12 TMSs. Larger proteins are present in relatively small numbers. In general, transport proteins often have 6 or 12 TMSs, although programs that predict topology are often in error by 1 or 2 TMSs [127]. Click here for file

Additional file 14 **Table S10 - complete listing of all predicted Ich protease-encoding genes**. Click here for file

Additional file 15 **Table S11 - comparative listing of protease-encoding gene classes in ciliates**. Click here for file

Additional file 16 **Figure S5 - comparison of Ich metabolic enzymes painted on KEGG pathways with those of *T. thermophila*, *P. tetraurelia *and *D. rerio***. For each pathway, hyperlinks are provided to view the relevant KEGG map painted in red foreground to indicate enzymes present in Ich and green background to indicate enzymes present in other organisms. Click here for file
