# Supplementary figures and images for: Comparative genomics of the pathogenic ciliate Ichthyophthirius multifiliis, its free-living relatives and a host species provide insights into adoption of a parasitic lifestyle and prospects for disease control
Source: Genome Biol. 2011 Oct 17;12(10):R100. doi: 10.1186/gb-2011-12-10-r100 (PMC3341644; doi:10.1186/gb-2011-12-10-r100)

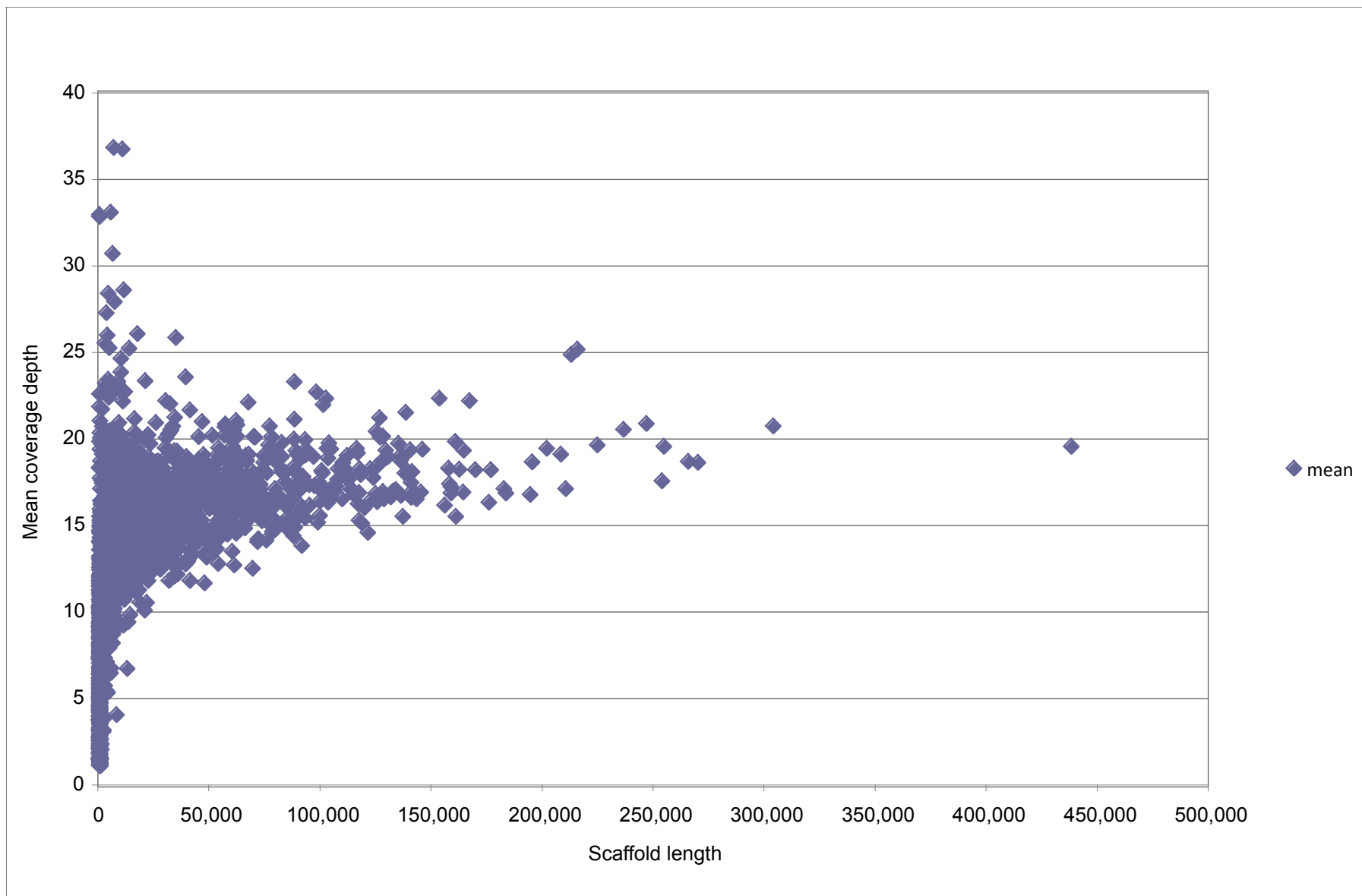

Supplement: Additional file 2 — Figure S1 - mean scaffold coverage depth. Mean coverage depth is plotted against scaffold length, showing that, for larger scaffolds, coverage does not diverge greatly from the mean. [file gb-2011-12-10-r100-S2.pdf]

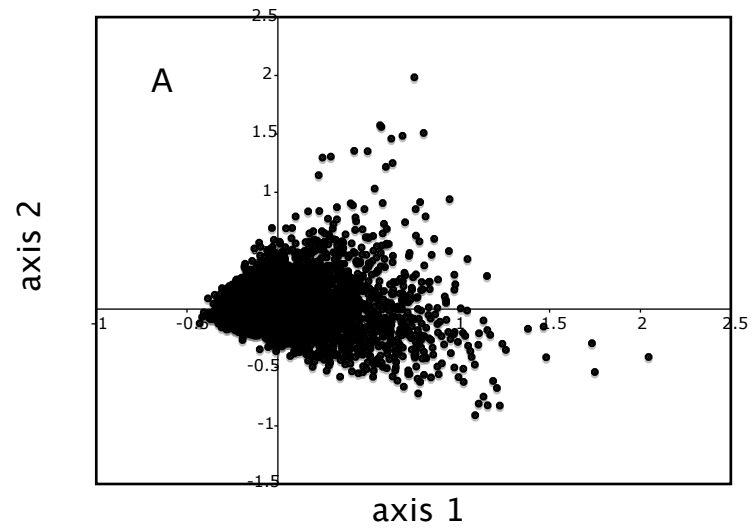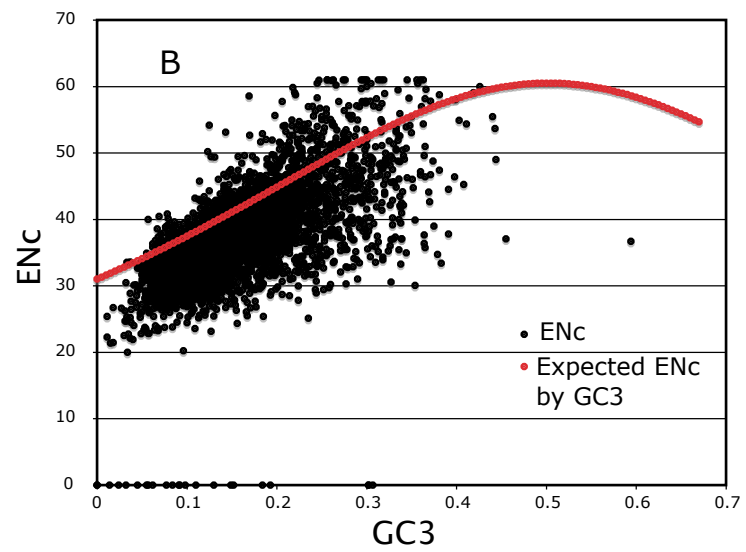

Supplement: Additional file 6 — Figure S2 - codon usage. (a) Principal component analysis of relative synonymous codon usage in Ich. (b) Effective number of codons (ENc; a measure of overall codon bias) for each predicted ORF is plotted versus GC3 (the fraction of codons that are synonymous at the third codon position that have either a guanine or a cytosine at that position). The upper limit of expected bias based on GC3 alone is represented by the red curve. [file gb-2011-12-10-r100-S6.PDF]

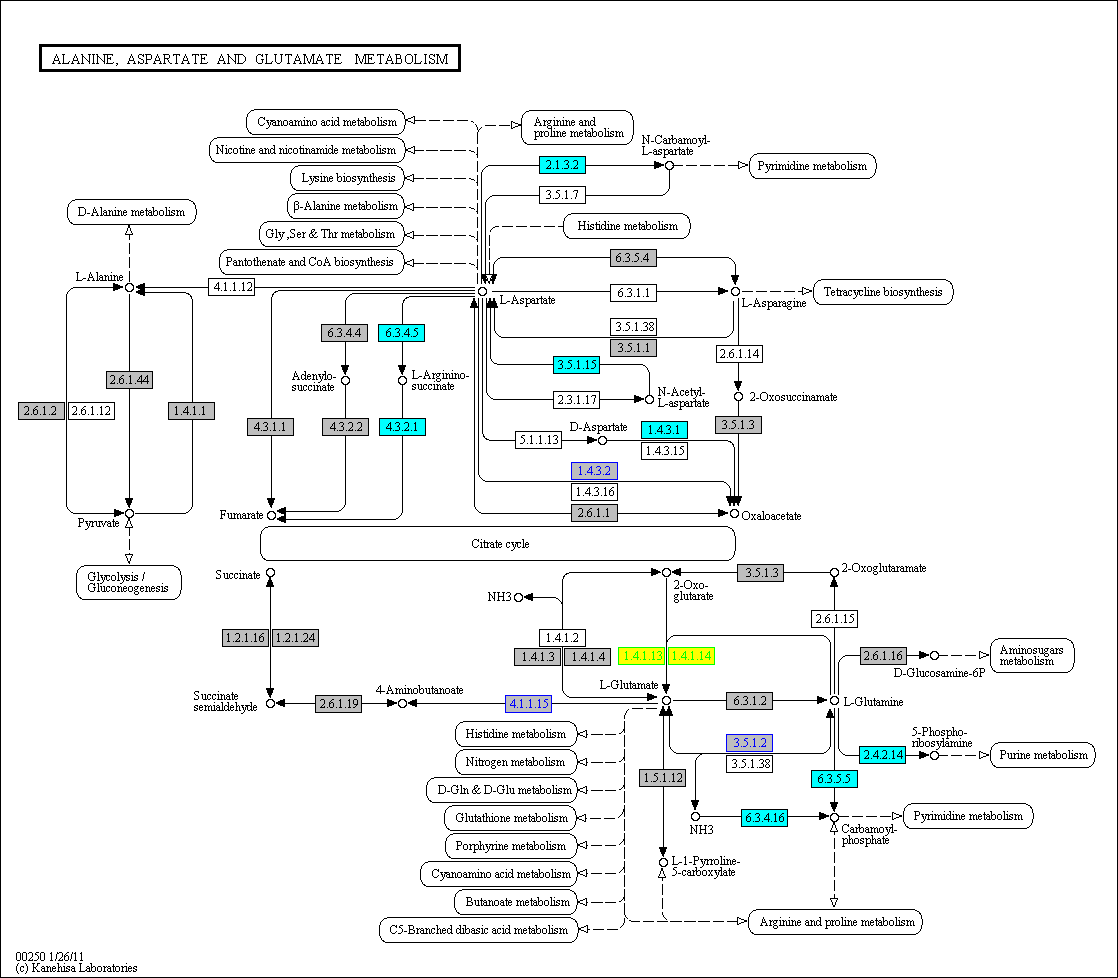

Supplement: Additional file 16 — Figure S5 - comparison of Ich metabolic enzymes painted on KEGG pathways with those of T. thermophila, P. tetraurelia and D. rerio. For each pathway, hyperlinks are provided to view the relevant KEGG map painted in red foreground to indicate enzymes present in Ich and green background to indicate enzymes present in other organisms. [file gb-2011-12-10-r100-S16.ZIP › Fig-S5/maps/Alanine&Aspartate&Glutamate.png]

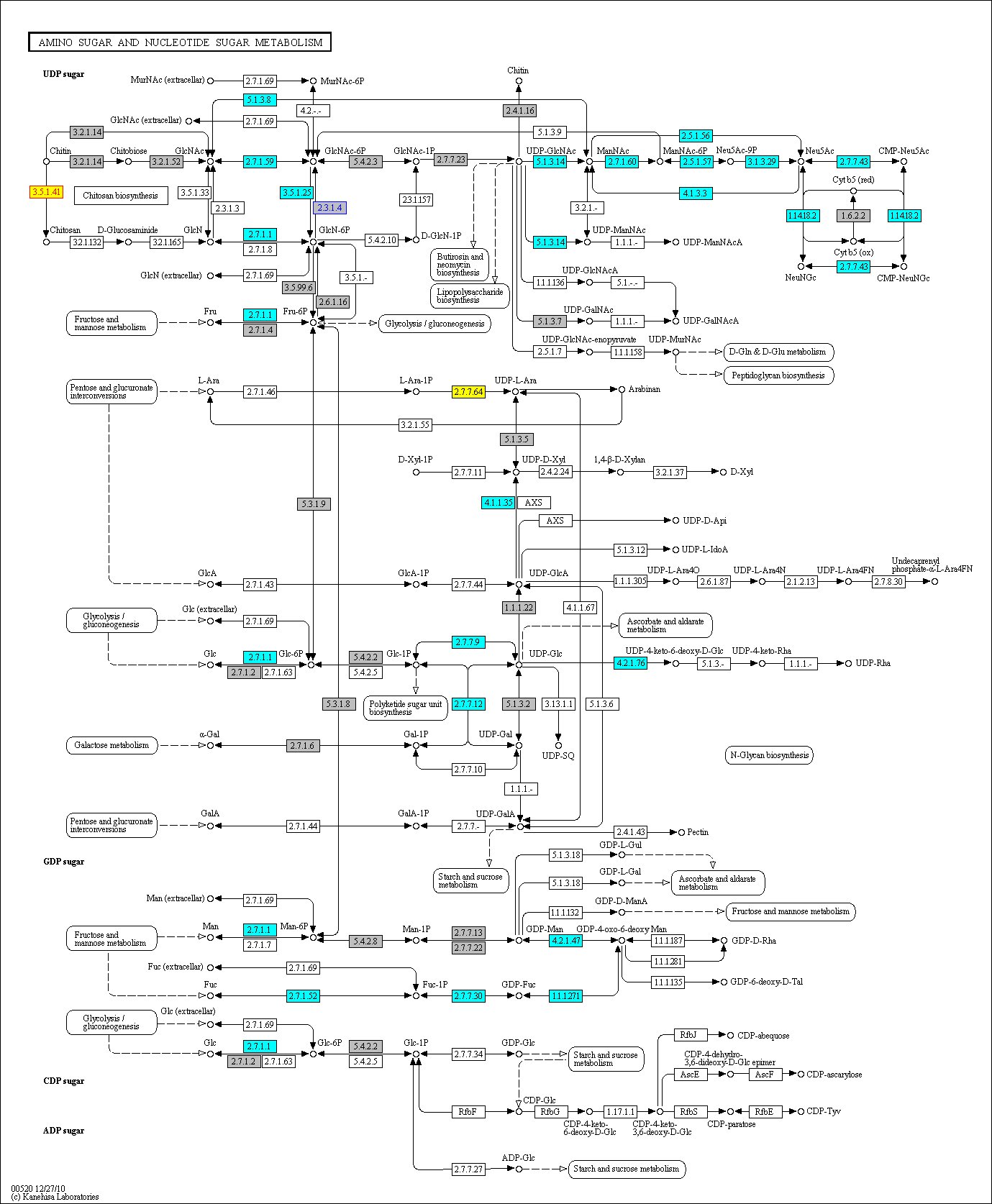

Supplement: Additional file 16 — Figure S5 - comparison of Ich metabolic enzymes painted on KEGG pathways with those of T. thermophila, P. tetraurelia and D. rerio. For each pathway, hyperlinks are provided to view the relevant KEGG map painted in red foreground to indicate enzymes present in Ich and green background to indicate enzymes present in other organisms. [file gb-2011-12-10-r100-S16.ZIP › Fig-S5/maps/Amino&Nucleotide-sugar.png]

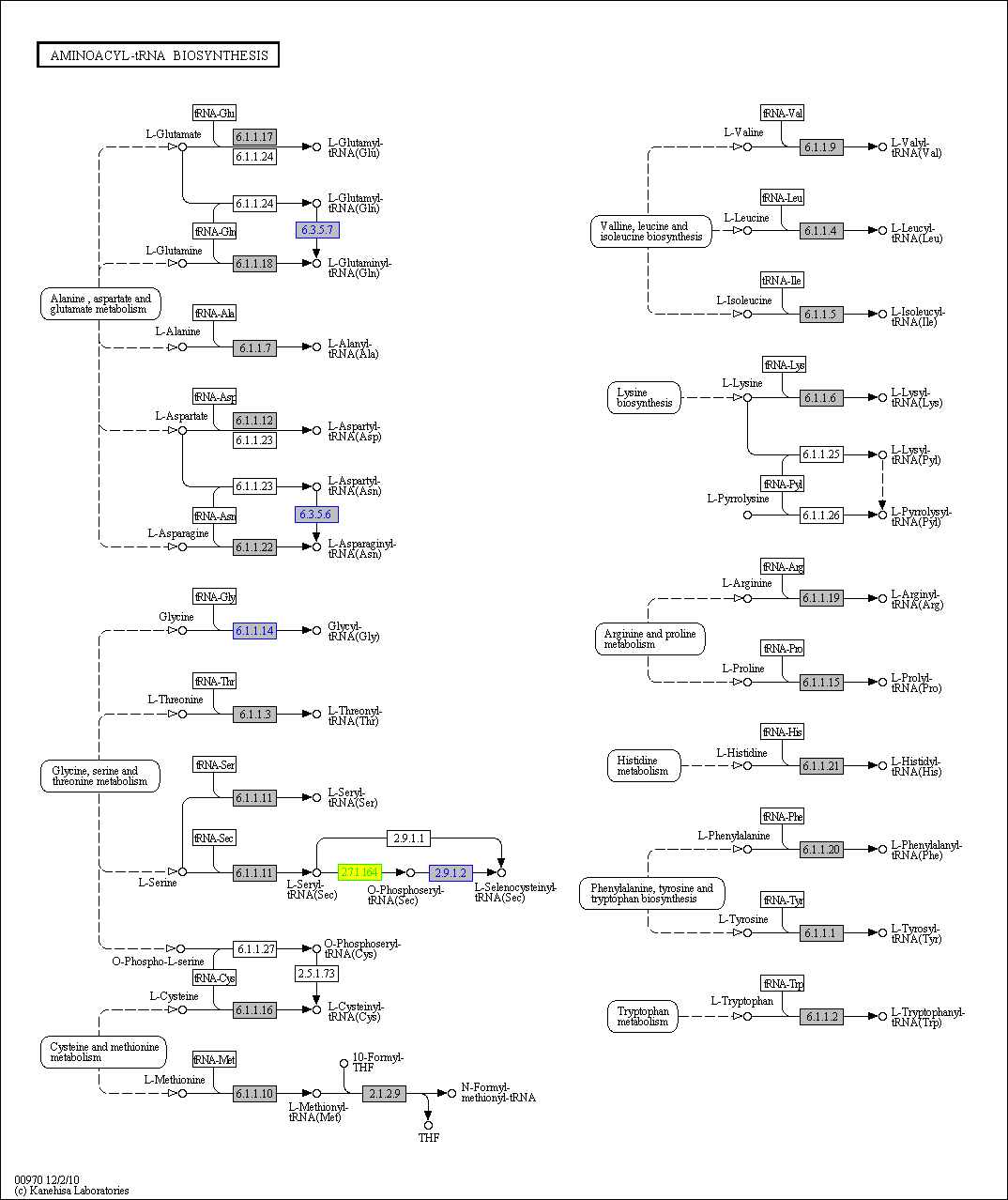

Supplement: Additional file 16 — Figure S5 - comparison of Ich metabolic enzymes painted on KEGG pathways with those of T. thermophila, P. tetraurelia and D. rerio. For each pathway, hyperlinks are provided to view the relevant KEGG map painted in red foreground to indicate enzymes present in Ich and green background to indicate enzymes present in other organisms. [file gb-2011-12-10-r100-S16.ZIP › Fig-S5/maps/Aminoacyl-tRNA.png]

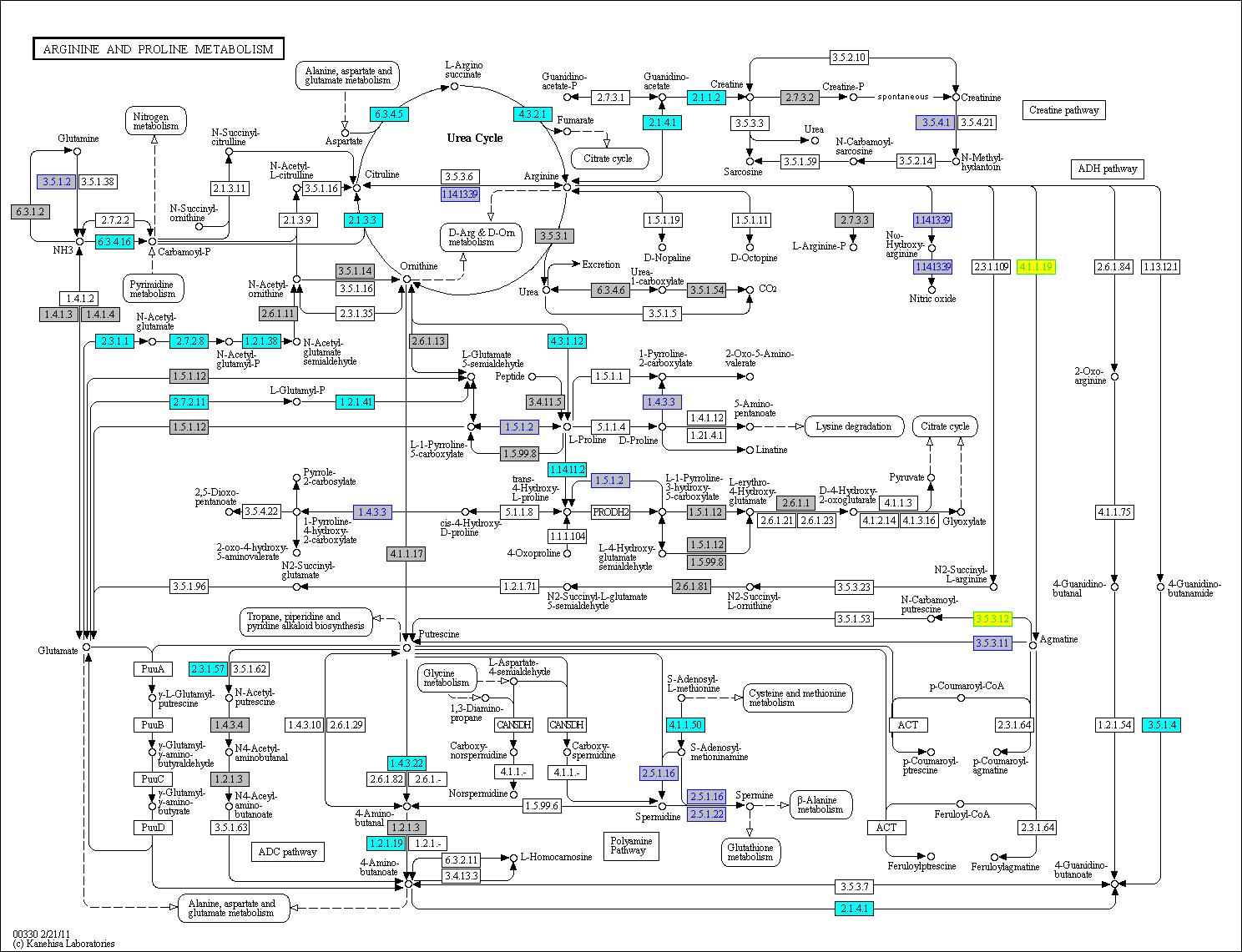

Supplement: Additional file 16 — Figure S5 - comparison of Ich metabolic enzymes painted on KEGG pathways with those of T. thermophila, P. tetraurelia and D. rerio. For each pathway, hyperlinks are provided to view the relevant KEGG map painted in red foreground to indicate enzymes present in Ich and green background to indicate enzymes present in other organisms. [file gb-2011-12-10-r100-S16.ZIP › Fig-S5/maps/Arginine&Proline.png]

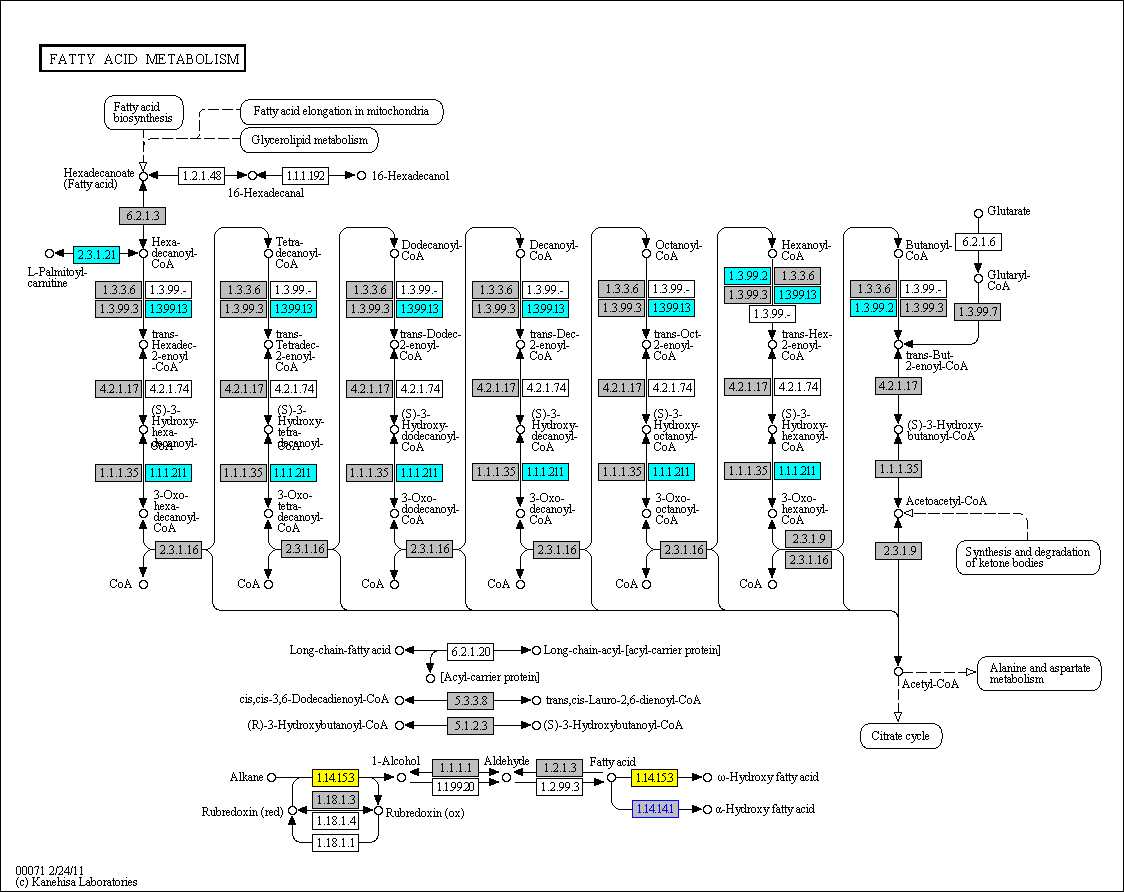

Supplement: Additional file 16 — Figure S5 - comparison of Ich metabolic enzymes painted on KEGG pathways with those of T. thermophila, P. tetraurelia and D. rerio. For each pathway, hyperlinks are provided to view the relevant KEGG map painted in red foreground to indicate enzymes present in Ich and green background to indicate enzymes present in other organisms. [file gb-2011-12-10-r100-S16.ZIP › Fig-S5/maps/betaOxidation-FA.png]

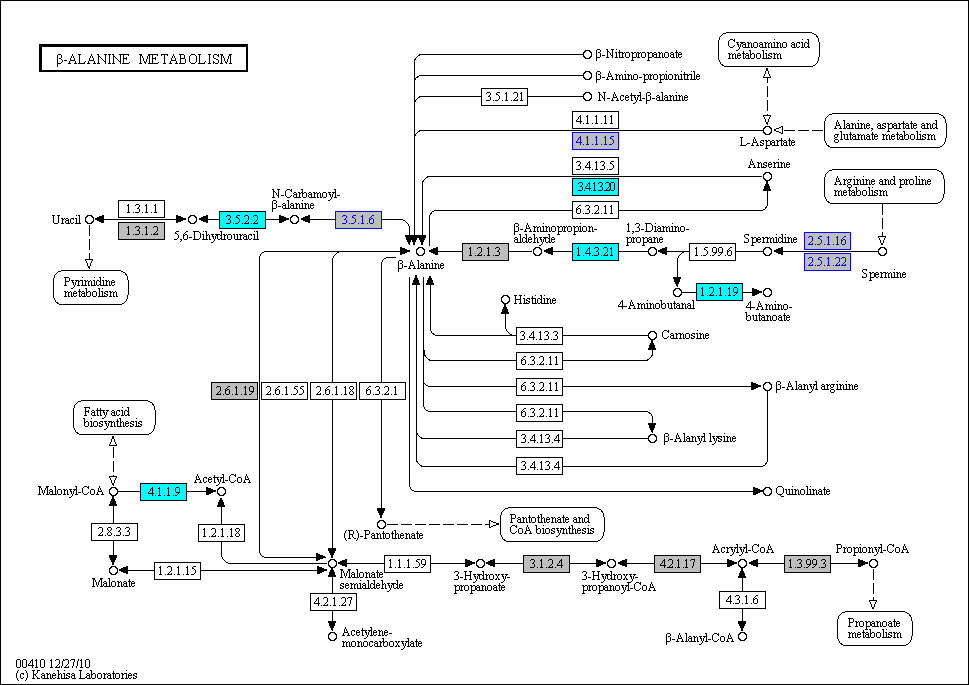

Supplement: Additional file 16 — Figure S5 - comparison of Ich metabolic enzymes painted on KEGG pathways with those of T. thermophila, P. tetraurelia and D. rerio. For each pathway, hyperlinks are provided to view the relevant KEGG map painted in red foreground to indicate enzymes present in Ich and green background to indicate enzymes present in other organisms. [file gb-2011-12-10-r100-S16.ZIP › Fig-S5/maps/Beta_alanine.png]

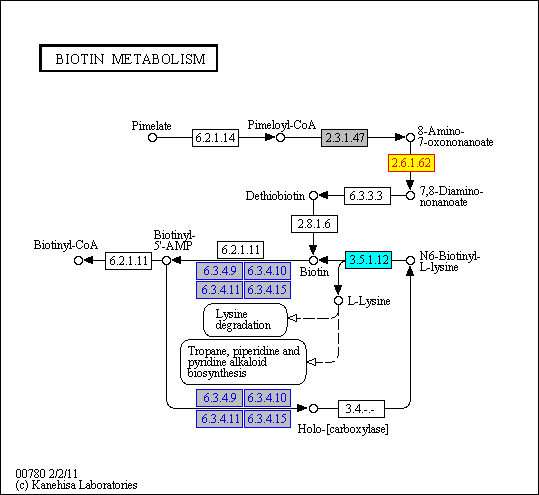

Supplement: Additional file 16 — Figure S5 - comparison of Ich metabolic enzymes painted on KEGG pathways with those of T. thermophila, P. tetraurelia and D. rerio. For each pathway, hyperlinks are provided to view the relevant KEGG map painted in red foreground to indicate enzymes present in Ich and green background to indicate enzymes present in other organisms. [file gb-2011-12-10-r100-S16.ZIP › Fig-S5/maps/Biotin.png]

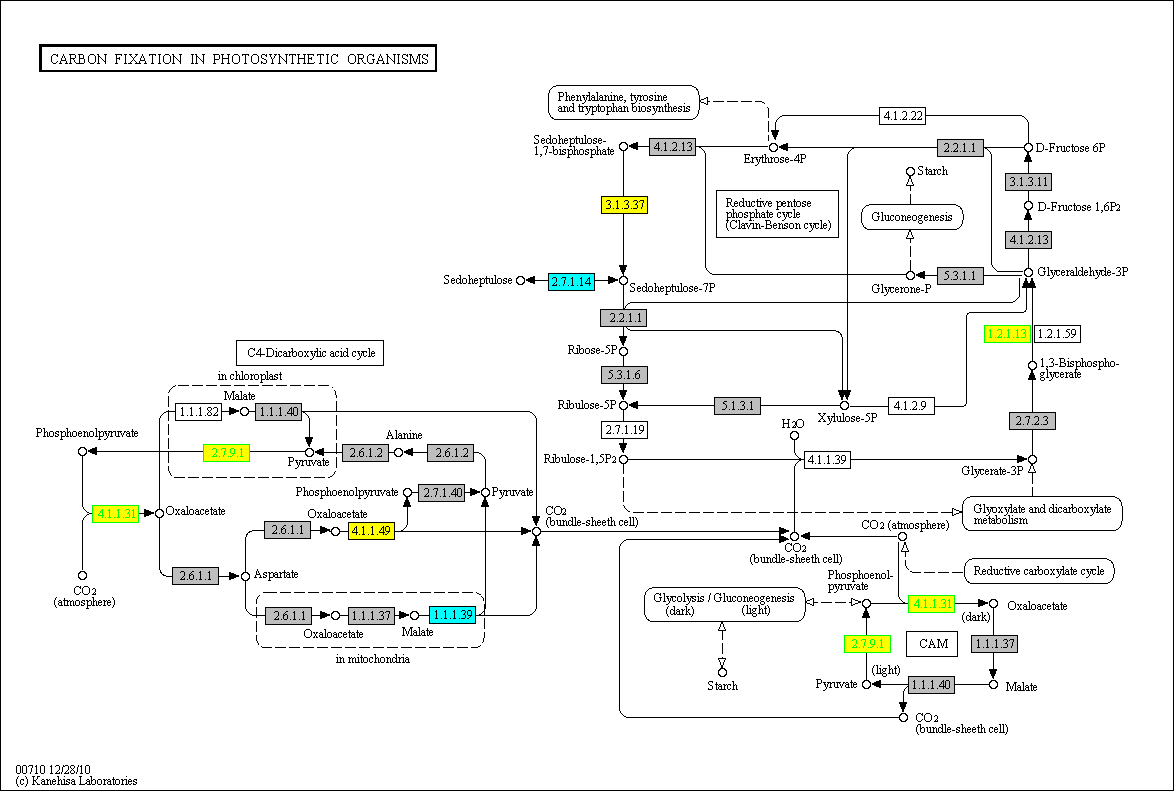

Supplement: Additional file 16 — Figure S5 - comparison of Ich metabolic enzymes painted on KEGG pathways with those of T. thermophila, P. tetraurelia and D. rerio. For each pathway, hyperlinks are provided to view the relevant KEGG map painted in red foreground to indicate enzymes present in Ich and green background to indicate enzymes present in other organisms. [file gb-2011-12-10-r100-S16.ZIP › Fig-S5/maps/carbonfixation_photosynthetic.png]

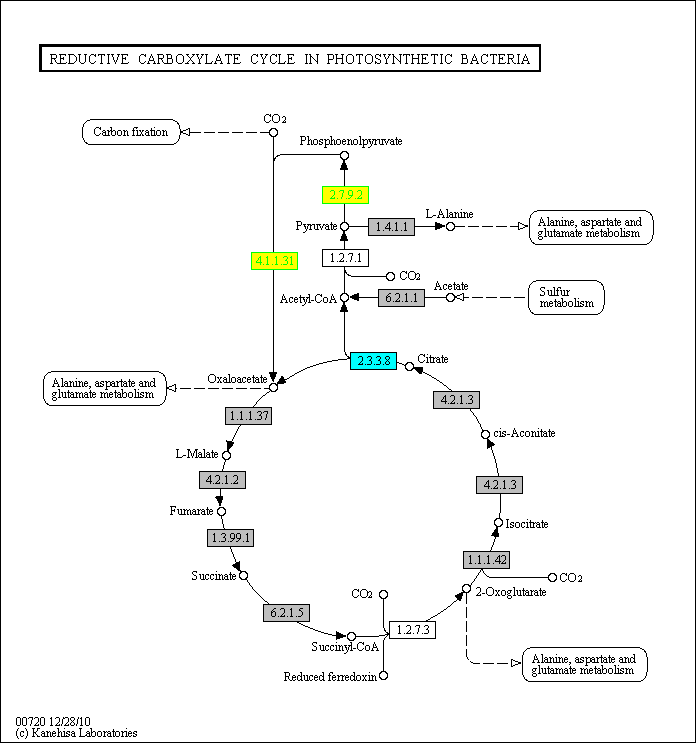

Supplement: Additional file 16 — Figure S5 - comparison of Ich metabolic enzymes painted on KEGG pathways with those of T. thermophila, P. tetraurelia and D. rerio. For each pathway, hyperlinks are provided to view the relevant KEGG map painted in red foreground to indicate enzymes present in Ich and green background to indicate enzymes present in other organisms. [file gb-2011-12-10-r100-S16.ZIP › Fig-S5/maps/carbonfixation_prokaryotic.png]

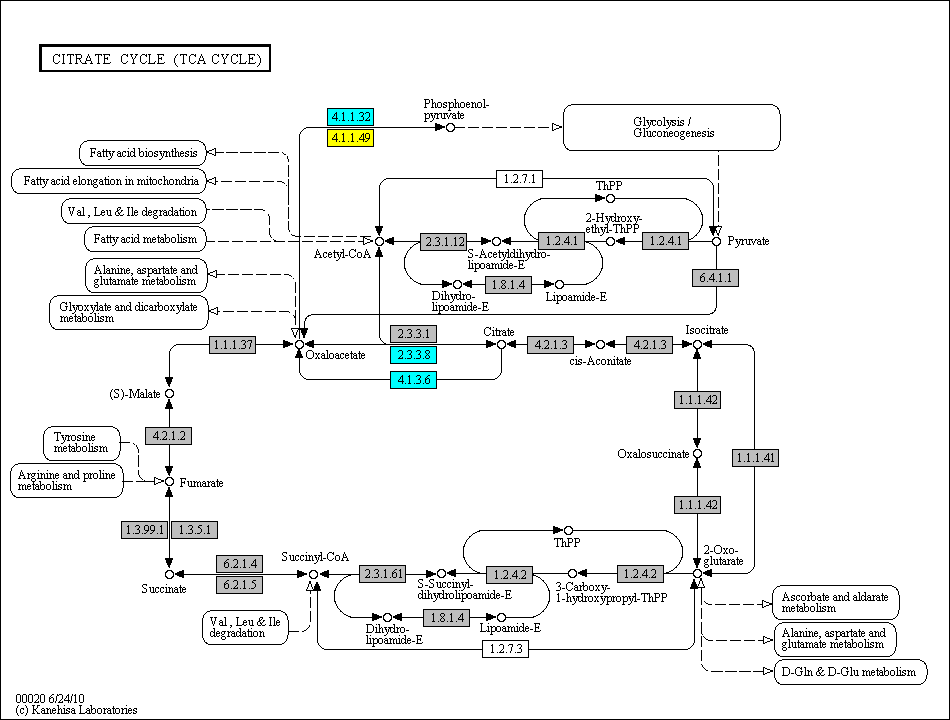

Supplement: Additional file 16 — Figure S5 - comparison of Ich metabolic enzymes painted on KEGG pathways with those of T. thermophila, P. tetraurelia and D. rerio. For each pathway, hyperlinks are provided to view the relevant KEGG map painted in red foreground to indicate enzymes present in Ich and green background to indicate enzymes present in other organisms. [file gb-2011-12-10-r100-S16.ZIP › Fig-S5/maps/CitrateCycle.png]

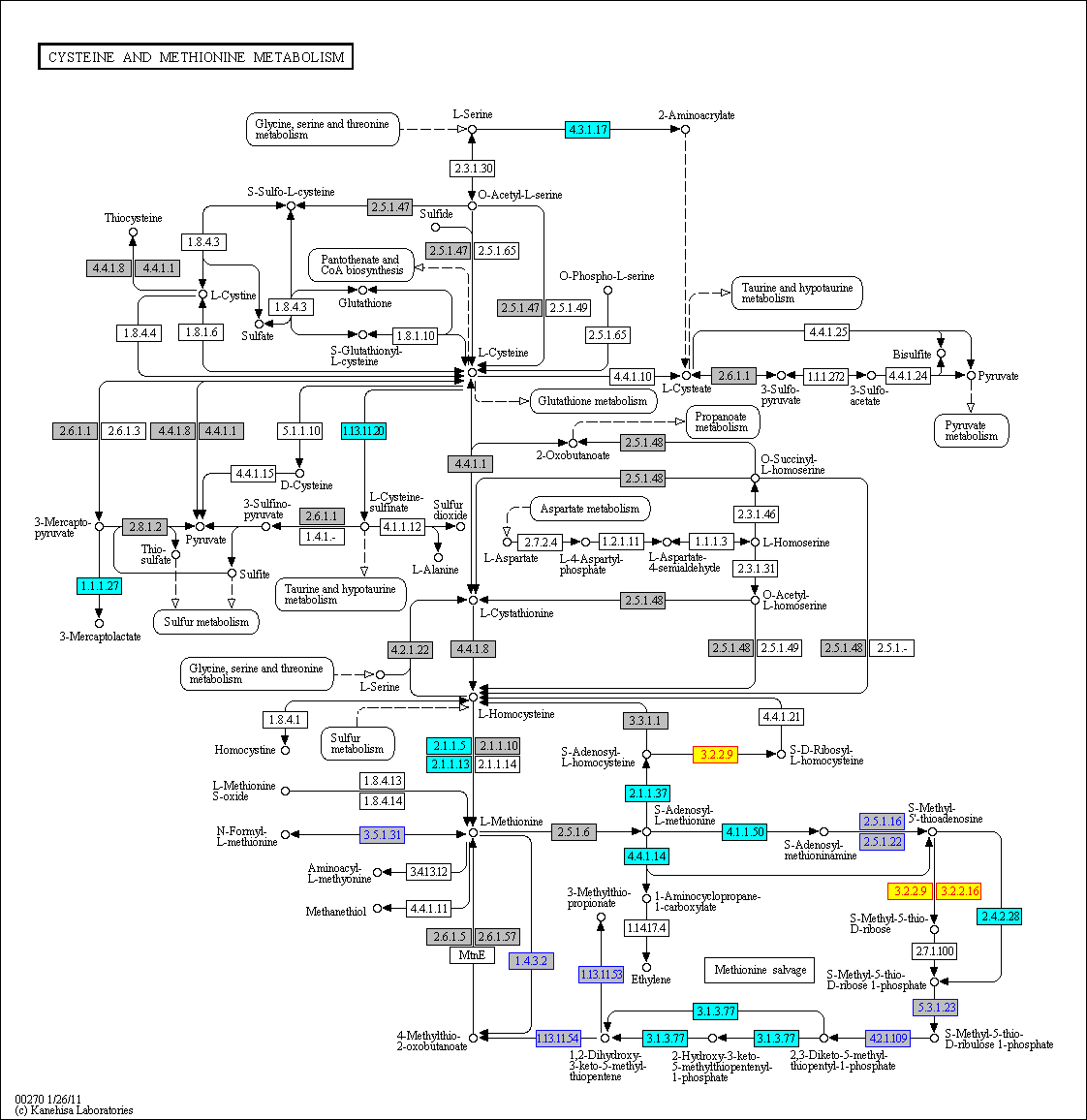

Supplement: Additional file 16 — Figure S5 - comparison of Ich metabolic enzymes painted on KEGG pathways with those of T. thermophila, P. tetraurelia and D. rerio. For each pathway, hyperlinks are provided to view the relevant KEGG map painted in red foreground to indicate enzymes present in Ich and green background to indicate enzymes present in other organisms. [file gb-2011-12-10-r100-S16.ZIP › Fig-S5/maps/Cysteine&Methionine.png]

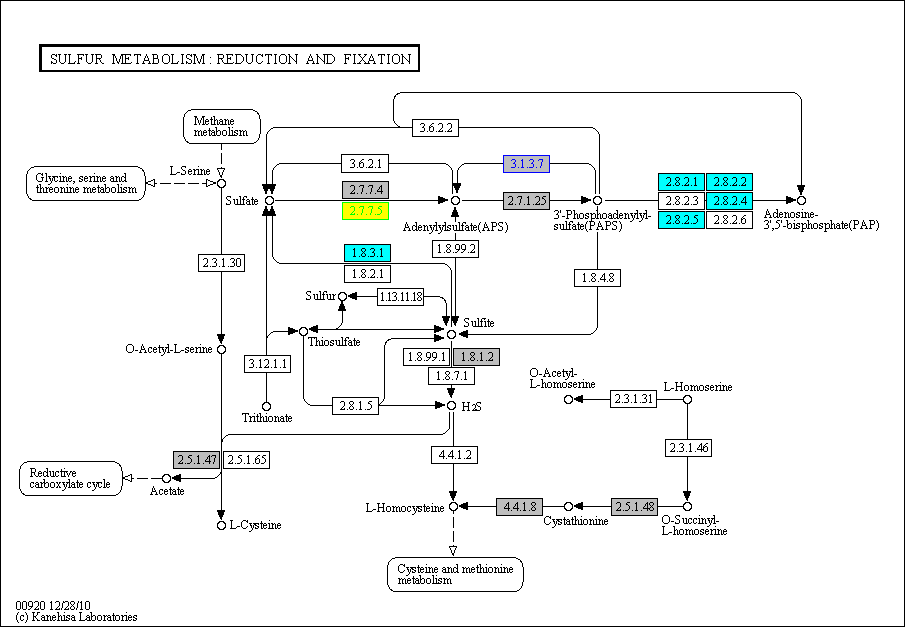

Supplement: Additional file 16 — Figure S5 - comparison of Ich metabolic enzymes painted on KEGG pathways with those of T. thermophila, P. tetraurelia and D. rerio. For each pathway, hyperlinks are provided to view the relevant KEGG map painted in red foreground to indicate enzymes present in Ich and green background to indicate enzymes present in other organisms. [file gb-2011-12-10-r100-S16.ZIP › Fig-S5/maps/ec00920.png]

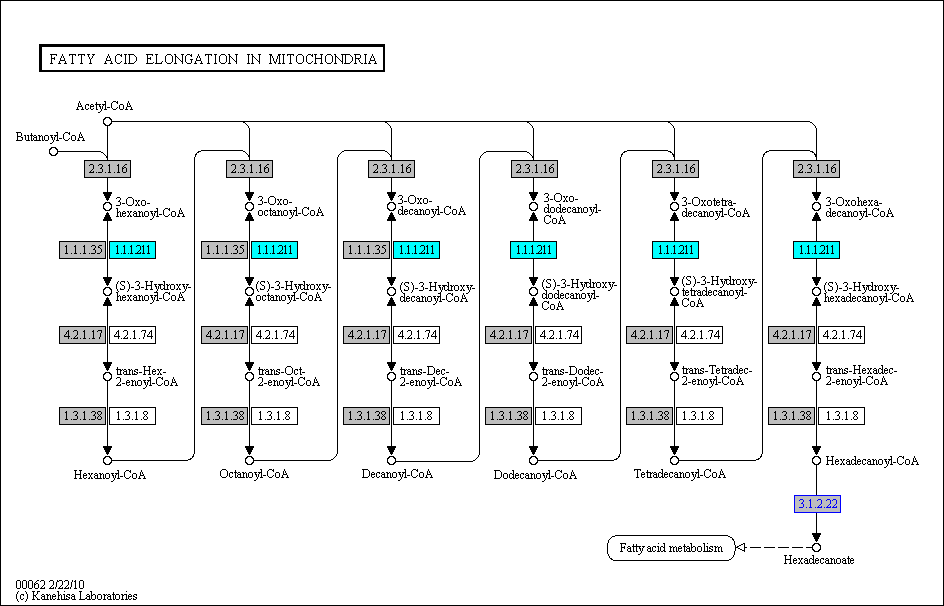

Supplement: Additional file 16 — Figure S5 - comparison of Ich metabolic enzymes painted on KEGG pathways with those of T. thermophila, P. tetraurelia and D. rerio. For each pathway, hyperlinks are provided to view the relevant KEGG map painted in red foreground to indicate enzymes present in Ich and green background to indicate enzymes present in other organisms. [file gb-2011-12-10-r100-S16.ZIP › Fig-S5/maps/FA-elongation.png]

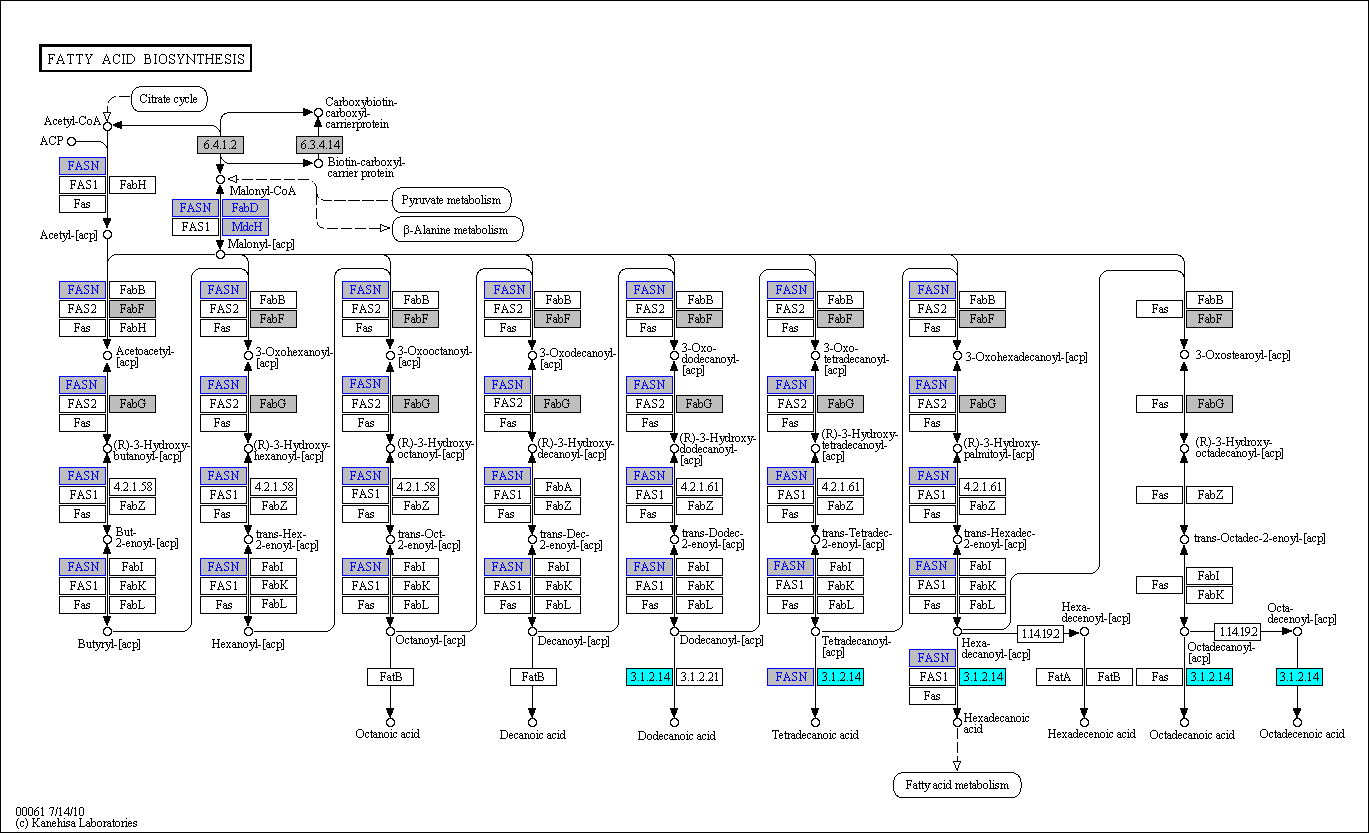

Supplement: Additional file 16 — Figure S5 - comparison of Ich metabolic enzymes painted on KEGG pathways with those of T. thermophila, P. tetraurelia and D. rerio. For each pathway, hyperlinks are provided to view the relevant KEGG map painted in red foreground to indicate enzymes present in Ich and green background to indicate enzymes present in other organisms. [file gb-2011-12-10-r100-S16.ZIP › Fig-S5/maps/FA-synthesis.png]

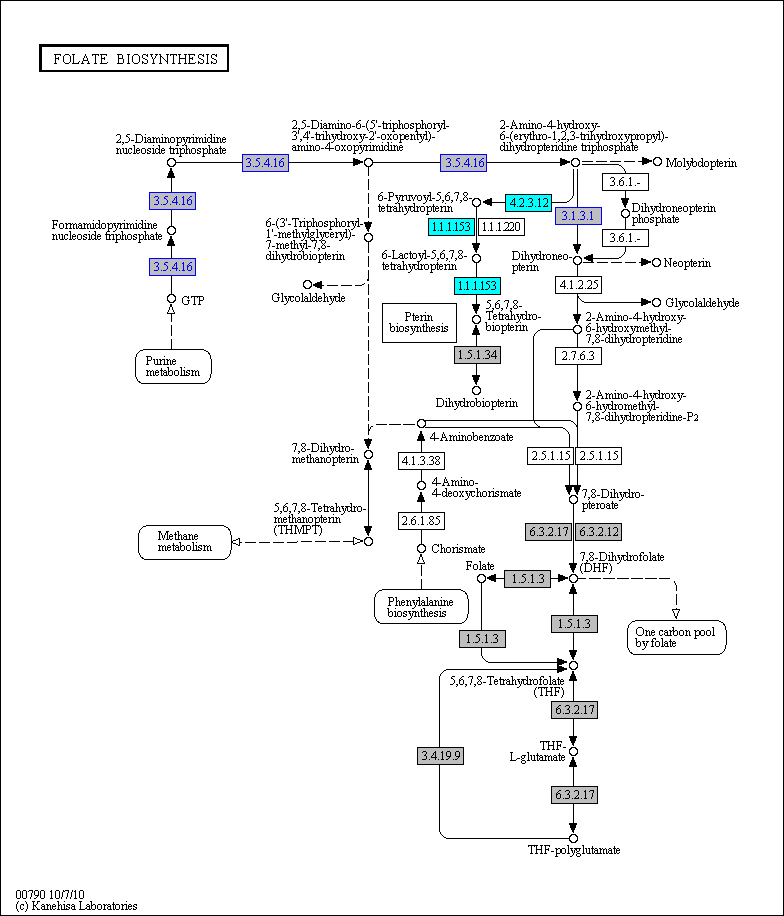

Supplement: Additional file 16 — Figure S5 - comparison of Ich metabolic enzymes painted on KEGG pathways with those of T. thermophila, P. tetraurelia and D. rerio. For each pathway, hyperlinks are provided to view the relevant KEGG map painted in red foreground to indicate enzymes present in Ich and green background to indicate enzymes present in other organisms. [file gb-2011-12-10-r100-S16.ZIP › Fig-S5/maps/Folate-biosynthesis.png]

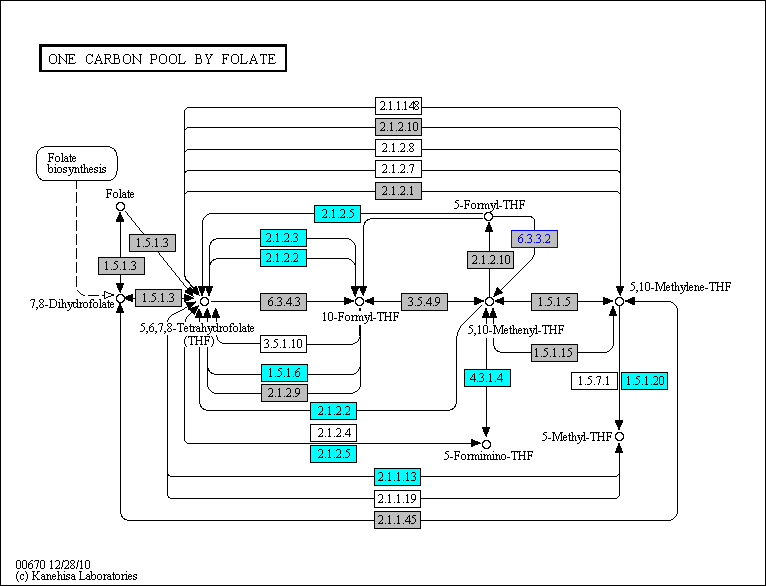

Supplement: Additional file 16 — Figure S5 - comparison of Ich metabolic enzymes painted on KEGG pathways with those of T. thermophila, P. tetraurelia and D. rerio. For each pathway, hyperlinks are provided to view the relevant KEGG map painted in red foreground to indicate enzymes present in Ich and green background to indicate enzymes present in other organisms. [file gb-2011-12-10-r100-S16.ZIP › Fig-S5/maps/Folate-oneCarbonPool.png]

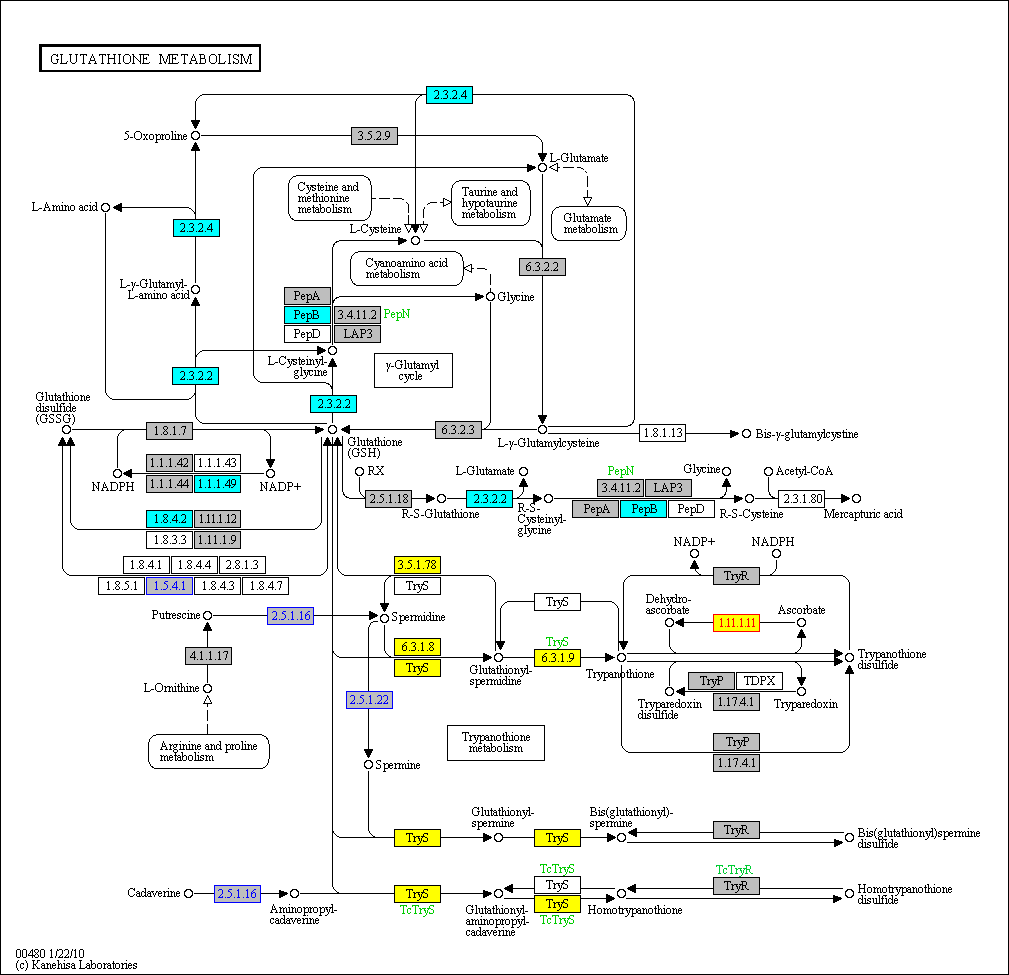

Supplement: Additional file 16 — Figure S5 - comparison of Ich metabolic enzymes painted on KEGG pathways with those of T. thermophila, P. tetraurelia and D. rerio. For each pathway, hyperlinks are provided to view the relevant KEGG map painted in red foreground to indicate enzymes present in Ich and green background to indicate enzymes present in other organisms. [file gb-2011-12-10-r100-S16.ZIP › Fig-S5/maps/Glutathione.png]

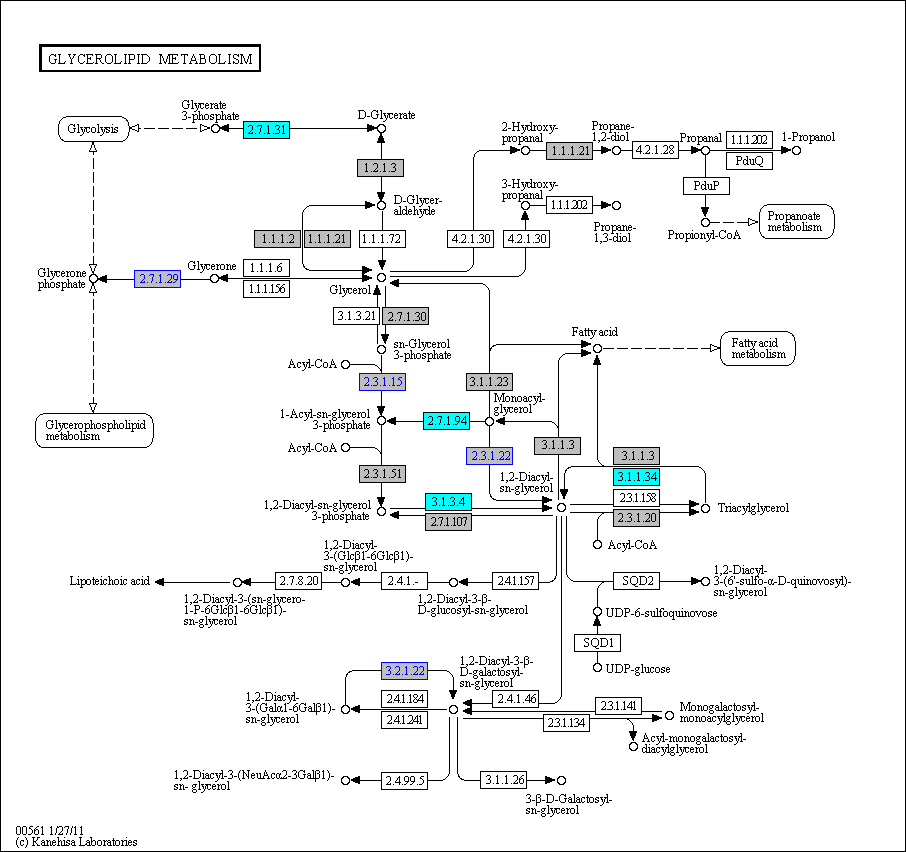

Supplement: Additional file 16 — Figure S5 - comparison of Ich metabolic enzymes painted on KEGG pathways with those of T. thermophila, P. tetraurelia and D. rerio. For each pathway, hyperlinks are provided to view the relevant KEGG map painted in red foreground to indicate enzymes present in Ich and green background to indicate enzymes present in other organisms. [file gb-2011-12-10-r100-S16.ZIP › Fig-S5/maps/Glycerolipid.png]

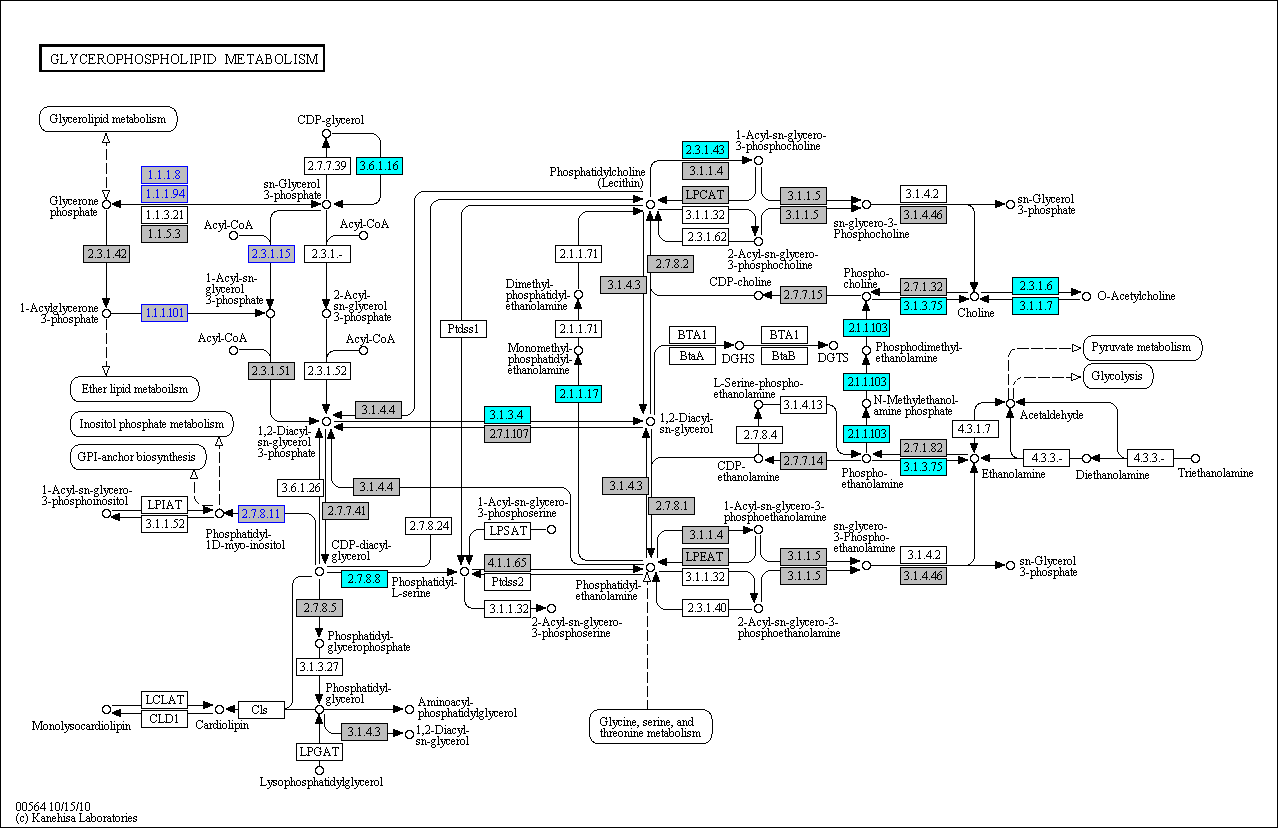

Supplement: Additional file 16 — Figure S5 - comparison of Ich metabolic enzymes painted on KEGG pathways with those of T. thermophila, P. tetraurelia and D. rerio. For each pathway, hyperlinks are provided to view the relevant KEGG map painted in red foreground to indicate enzymes present in Ich and green background to indicate enzymes present in other organisms. [file gb-2011-12-10-r100-S16.ZIP › Fig-S5/maps/Glycerophospholipid.png]

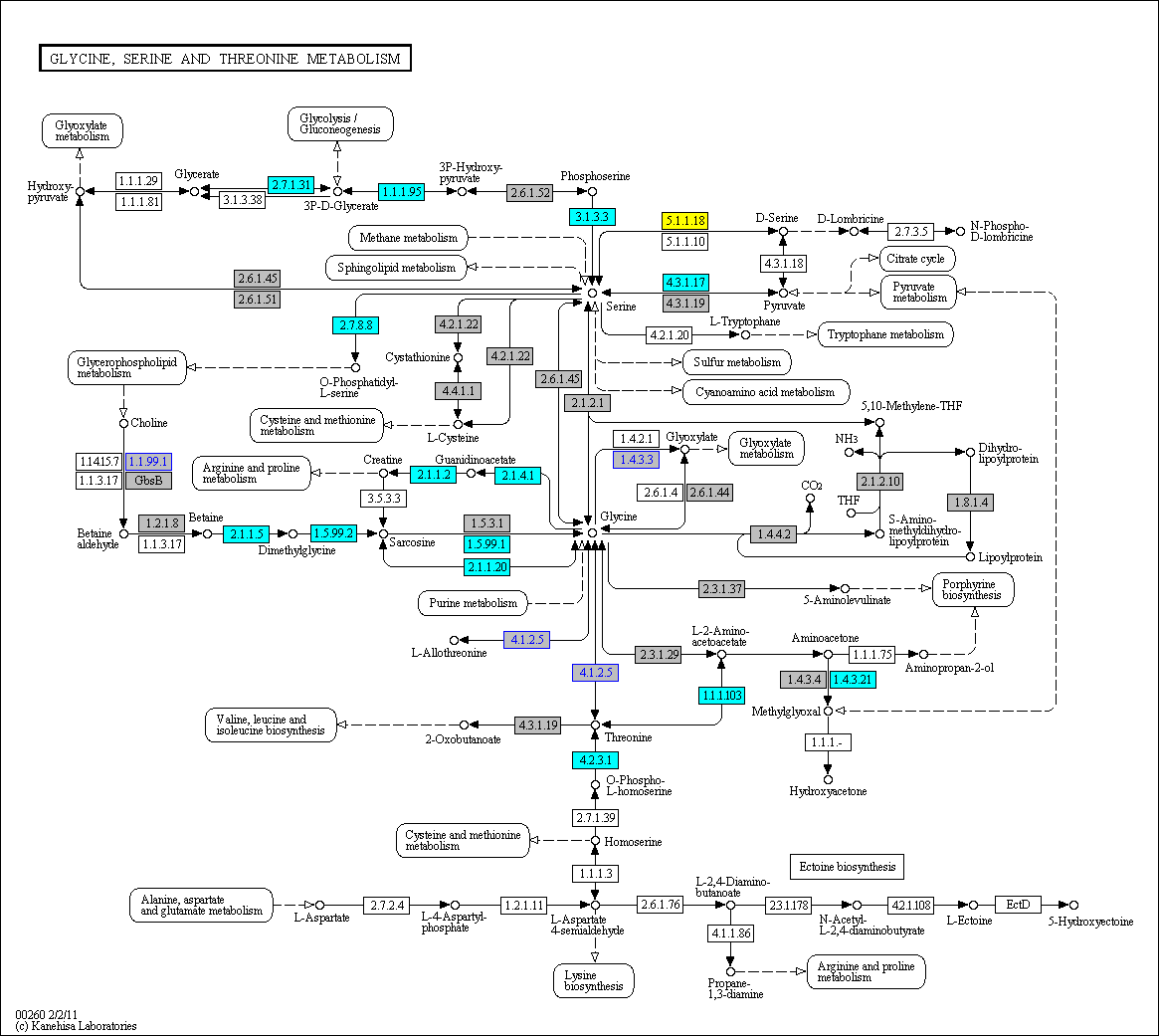

Supplement: Additional file 16 — Figure S5 - comparison of Ich metabolic enzymes painted on KEGG pathways with those of T. thermophila, P. tetraurelia and D. rerio. For each pathway, hyperlinks are provided to view the relevant KEGG map painted in red foreground to indicate enzymes present in Ich and green background to indicate enzymes present in other organisms. [file gb-2011-12-10-r100-S16.ZIP › Fig-S5/maps/Glycine&serine&Threonine.png]

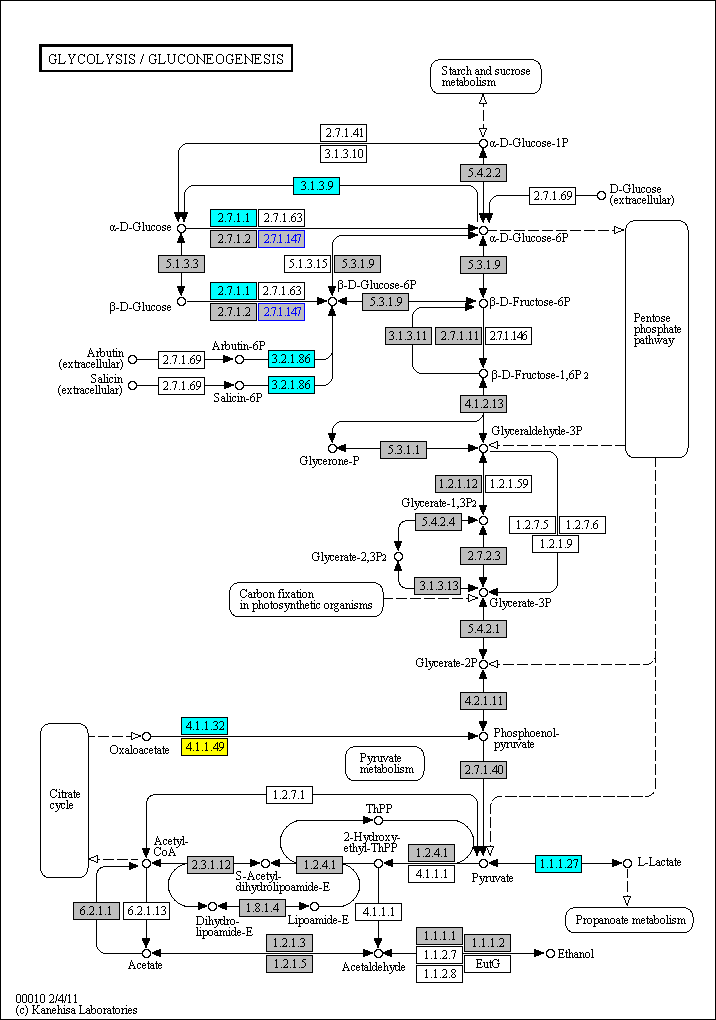

Supplement: Additional file 16 — Figure S5 - comparison of Ich metabolic enzymes painted on KEGG pathways with those of T. thermophila, P. tetraurelia and D. rerio. For each pathway, hyperlinks are provided to view the relevant KEGG map painted in red foreground to indicate enzymes present in Ich and green background to indicate enzymes present in other organisms. [file gb-2011-12-10-r100-S16.ZIP › Fig-S5/maps/glycolysis.png]

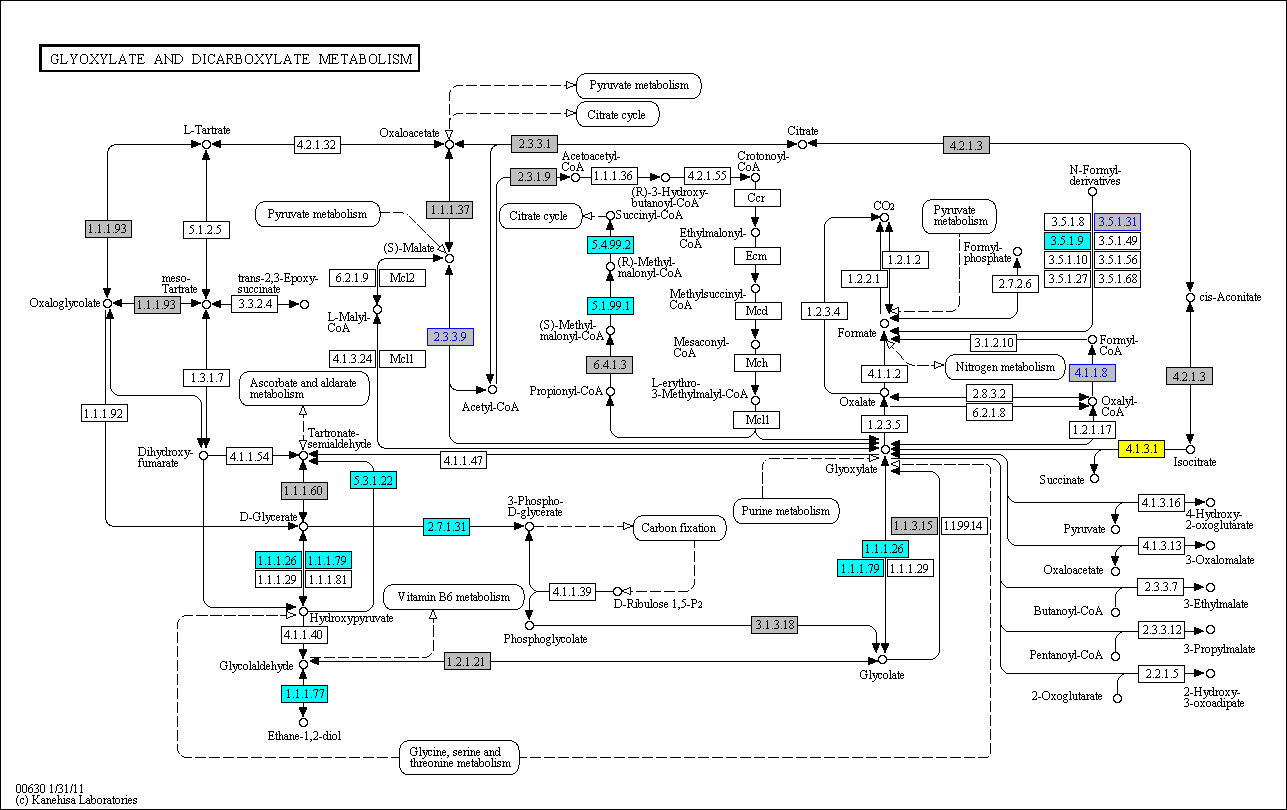

Supplement: Additional file 16 — Figure S5 - comparison of Ich metabolic enzymes painted on KEGG pathways with those of T. thermophila, P. tetraurelia and D. rerio. For each pathway, hyperlinks are provided to view the relevant KEGG map painted in red foreground to indicate enzymes present in Ich and green background to indicate enzymes present in other organisms. [file gb-2011-12-10-r100-S16.ZIP › Fig-S5/maps/Glyoxalate&dicarboxylate.png]

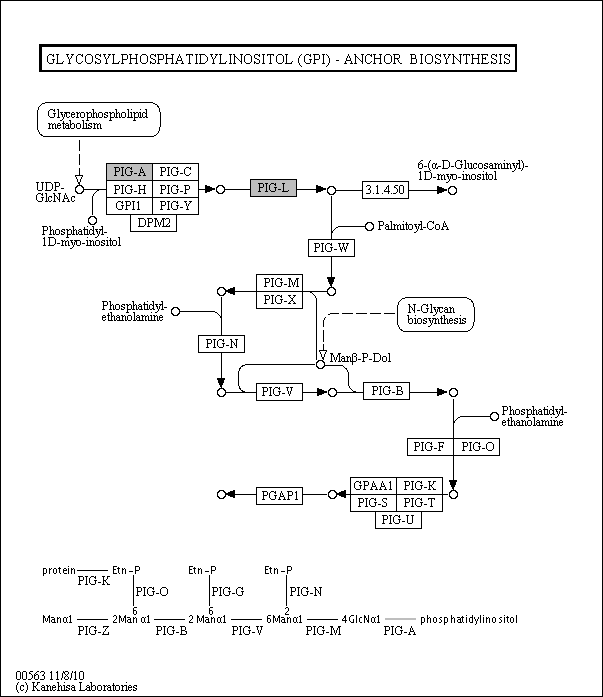

Supplement: Additional file 16 — Figure S5 - comparison of Ich metabolic enzymes painted on KEGG pathways with those of T. thermophila, P. tetraurelia and D. rerio. For each pathway, hyperlinks are provided to view the relevant KEGG map painted in red foreground to indicate enzymes present in Ich and green background to indicate enzymes present in other organisms. [file gb-2011-12-10-r100-S16.ZIP › Fig-S5/maps/GPI-anchor.png]

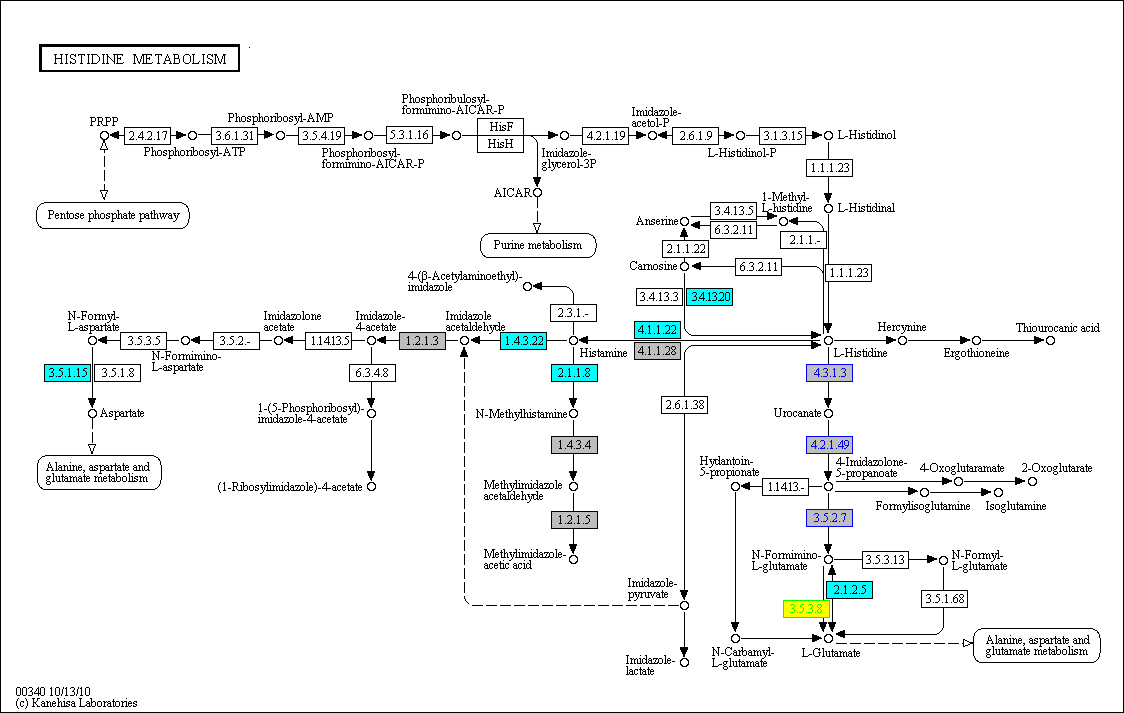

Supplement: Additional file 16 — Figure S5 - comparison of Ich metabolic enzymes painted on KEGG pathways with those of T. thermophila, P. tetraurelia and D. rerio. For each pathway, hyperlinks are provided to view the relevant KEGG map painted in red foreground to indicate enzymes present in Ich and green background to indicate enzymes present in other organisms. [file gb-2011-12-10-r100-S16.ZIP › Fig-S5/maps/histidine.png]

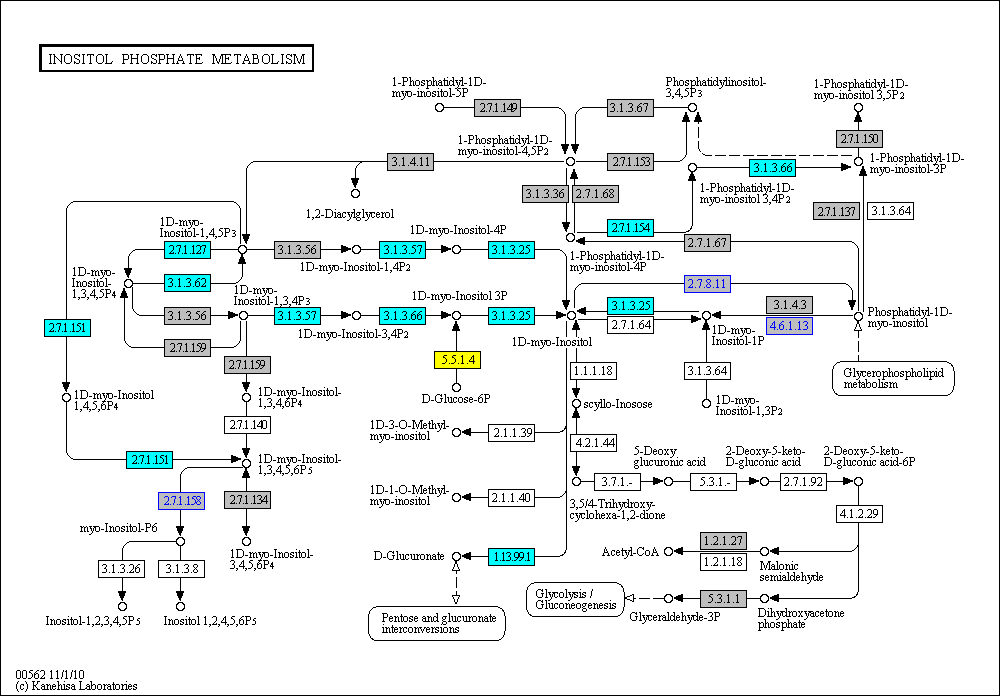

Supplement: Additional file 16 — Figure S5 - comparison of Ich metabolic enzymes painted on KEGG pathways with those of T. thermophila, P. tetraurelia and D. rerio. For each pathway, hyperlinks are provided to view the relevant KEGG map painted in red foreground to indicate enzymes present in Ich and green background to indicate enzymes present in other organisms. [file gb-2011-12-10-r100-S16.ZIP › Fig-S5/maps/Inositol-P.png]

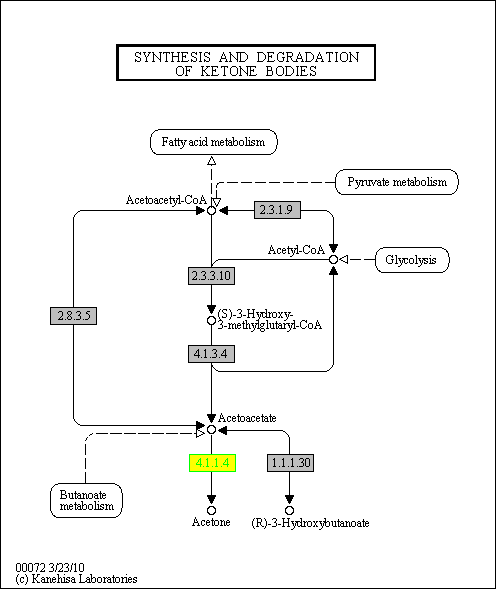

Supplement: Additional file 16 — Figure S5 - comparison of Ich metabolic enzymes painted on KEGG pathways with those of T. thermophila, P. tetraurelia and D. rerio. For each pathway, hyperlinks are provided to view the relevant KEGG map painted in red foreground to indicate enzymes present in Ich and green background to indicate enzymes present in other organisms. [file gb-2011-12-10-r100-S16.ZIP › Fig-S5/maps/ketoneBodies.png]

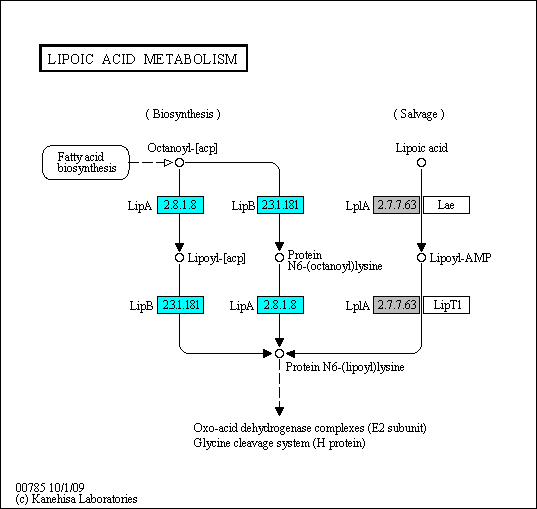

Supplement: Additional file 16 — Figure S5 - comparison of Ich metabolic enzymes painted on KEGG pathways with those of T. thermophila, P. tetraurelia and D. rerio. For each pathway, hyperlinks are provided to view the relevant KEGG map painted in red foreground to indicate enzymes present in Ich and green background to indicate enzymes present in other organisms. [file gb-2011-12-10-r100-S16.ZIP › Fig-S5/maps/Lipoate.png]

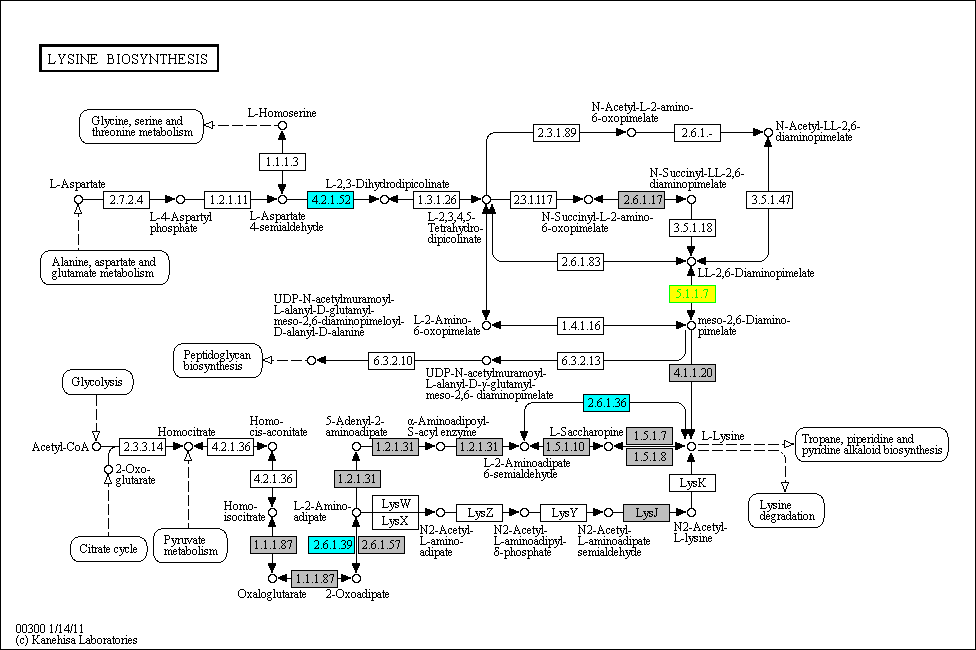

Supplement: Additional file 16 — Figure S5 - comparison of Ich metabolic enzymes painted on KEGG pathways with those of T. thermophila, P. tetraurelia and D. rerio. For each pathway, hyperlinks are provided to view the relevant KEGG map painted in red foreground to indicate enzymes present in Ich and green background to indicate enzymes present in other organisms. [file gb-2011-12-10-r100-S16.ZIP › Fig-S5/maps/Lysine-biosynthesis.png]

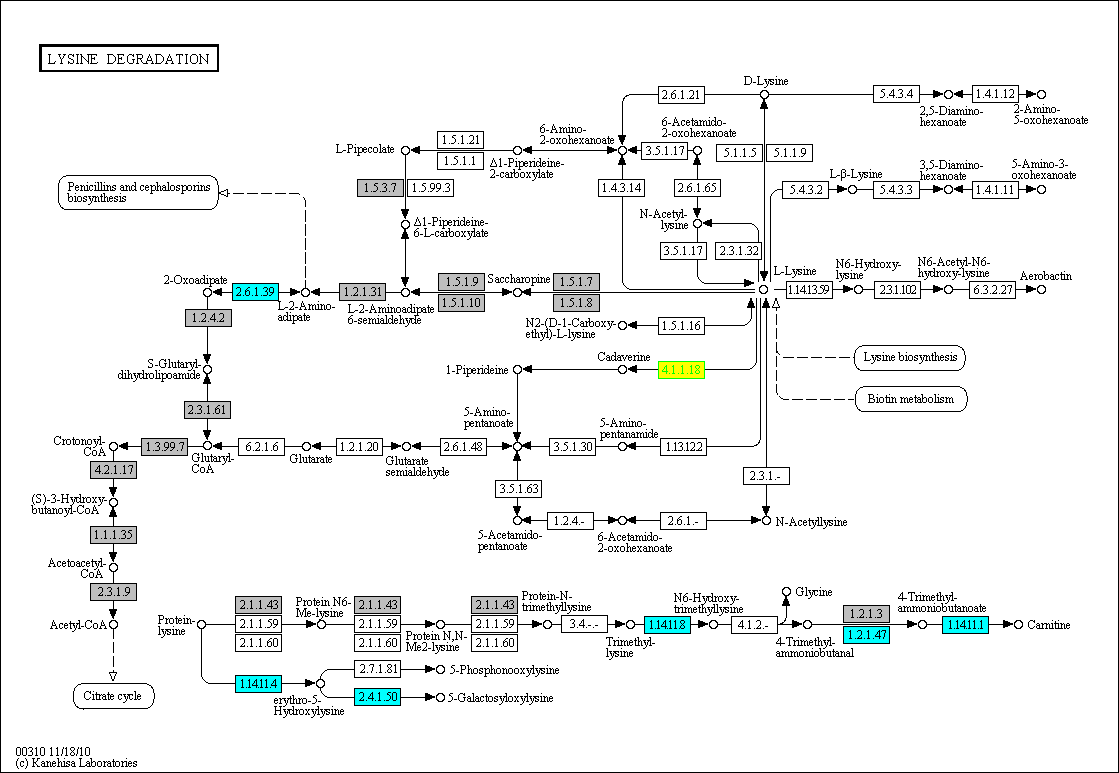

Supplement: Additional file 16 — Figure S5 - comparison of Ich metabolic enzymes painted on KEGG pathways with those of T. thermophila, P. tetraurelia and D. rerio. For each pathway, hyperlinks are provided to view the relevant KEGG map painted in red foreground to indicate enzymes present in Ich and green background to indicate enzymes present in other organisms. [file gb-2011-12-10-r100-S16.ZIP › Fig-S5/maps/Lysine-degradation.png]

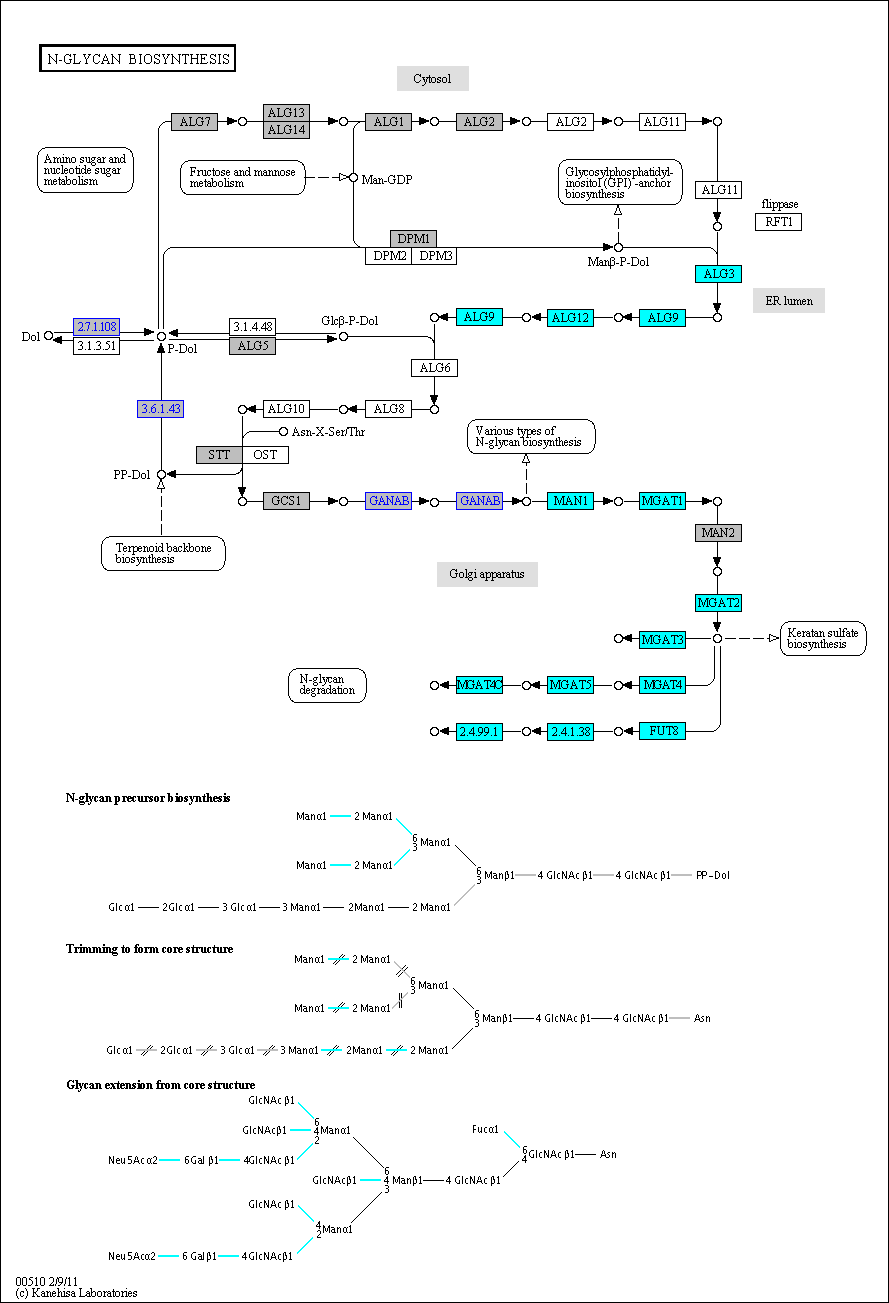

Supplement: Additional file 16 — Figure S5 - comparison of Ich metabolic enzymes painted on KEGG pathways with those of T. thermophila, P. tetraurelia and D. rerio. For each pathway, hyperlinks are provided to view the relevant KEGG map painted in red foreground to indicate enzymes present in Ich and green background to indicate enzymes present in other organisms. [file gb-2011-12-10-r100-S16.ZIP › Fig-S5/maps/N-glycan.png]

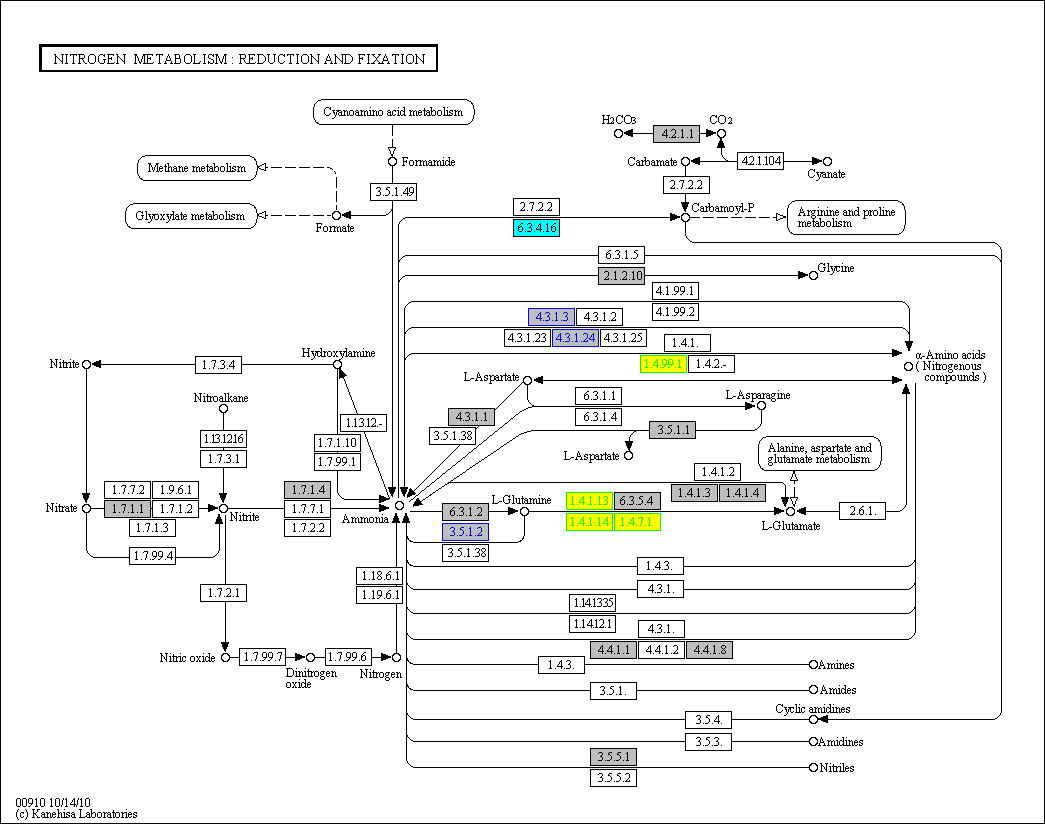

Supplement: Additional file 16 — Figure S5 - comparison of Ich metabolic enzymes painted on KEGG pathways with those of T. thermophila, P. tetraurelia and D. rerio. For each pathway, hyperlinks are provided to view the relevant KEGG map painted in red foreground to indicate enzymes present in Ich and green background to indicate enzymes present in other organisms. [file gb-2011-12-10-r100-S16.ZIP › Fig-S5/maps/Nitrogen.png]

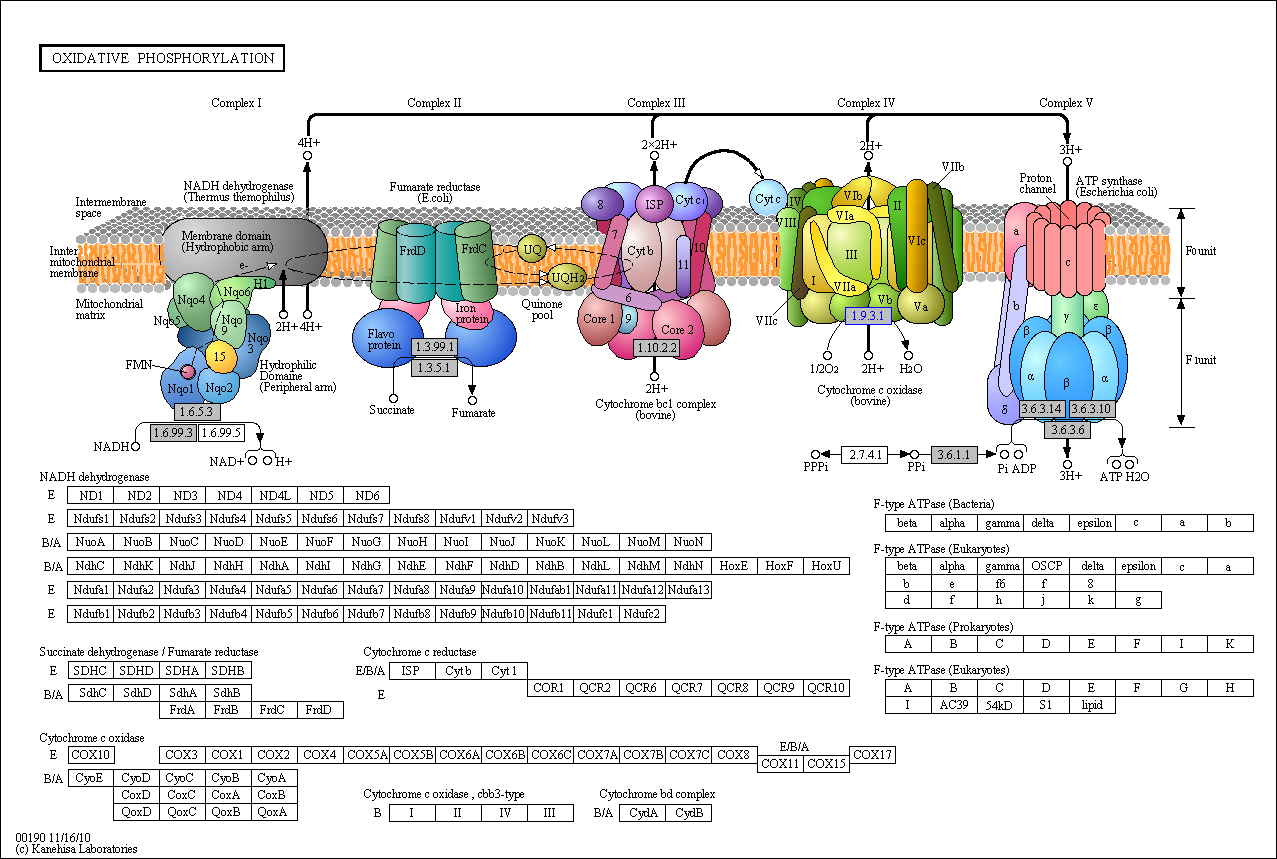

Supplement: Additional file 16 — Figure S5 - comparison of Ich metabolic enzymes painted on KEGG pathways with those of T. thermophila, P. tetraurelia and D. rerio. For each pathway, hyperlinks are provided to view the relevant KEGG map painted in red foreground to indicate enzymes present in Ich and green background to indicate enzymes present in other organisms. [file gb-2011-12-10-r100-S16.ZIP › Fig-S5/maps/OxPhos.png]

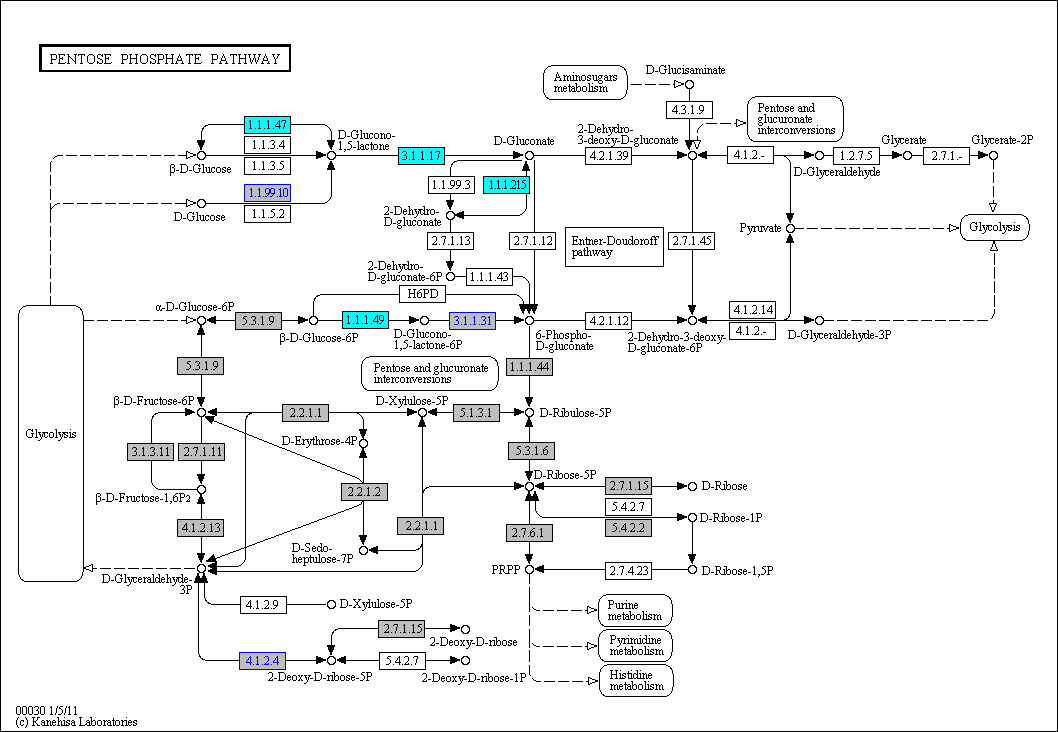

Supplement: Additional file 16 — Figure S5 - comparison of Ich metabolic enzymes painted on KEGG pathways with those of T. thermophila, P. tetraurelia and D. rerio. For each pathway, hyperlinks are provided to view the relevant KEGG map painted in red foreground to indicate enzymes present in Ich and green background to indicate enzymes present in other organisms. [file gb-2011-12-10-r100-S16.ZIP › Fig-S5/maps/Pentose-P_pathway.png]

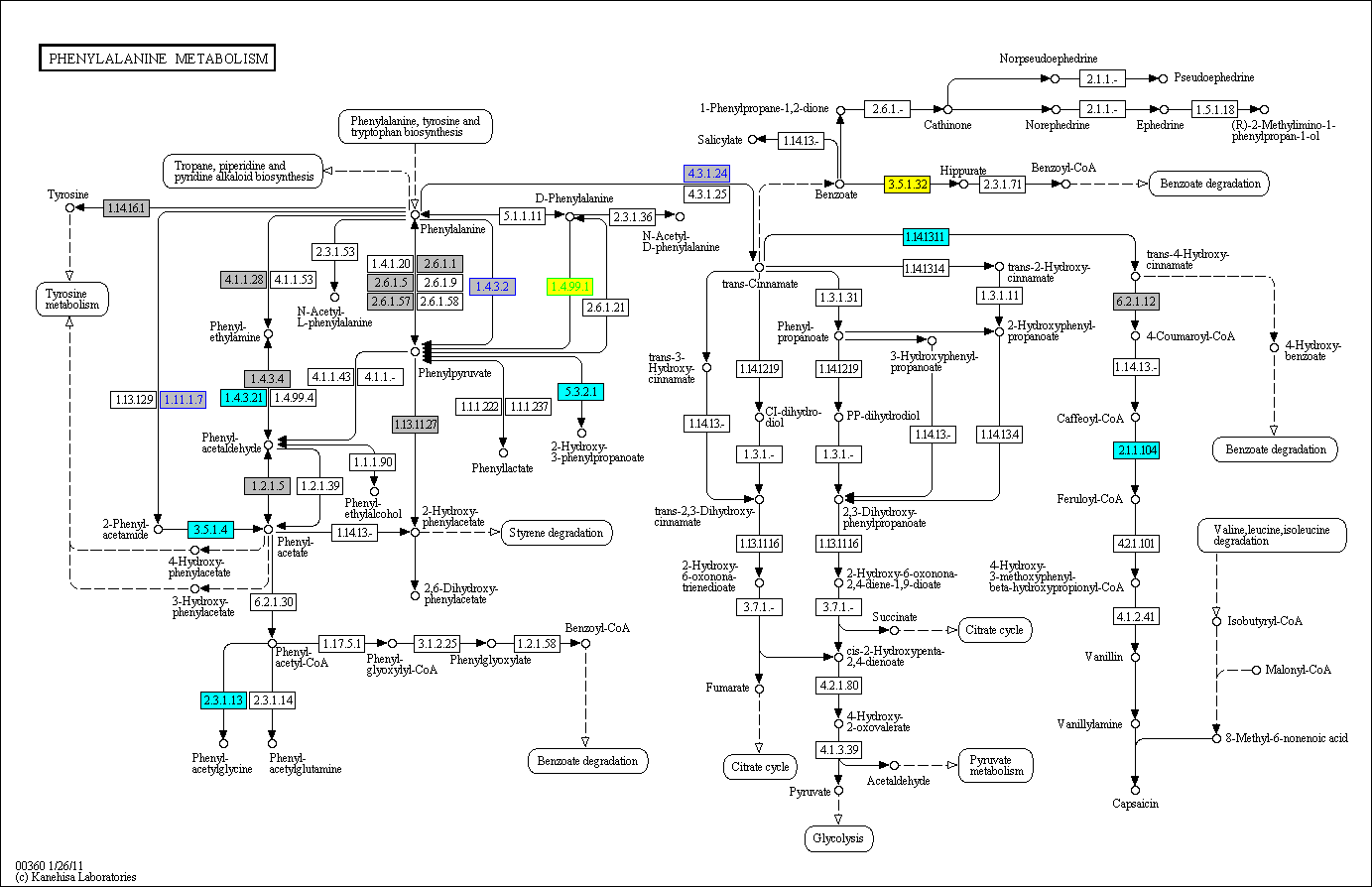

Supplement: Additional file 16 — Figure S5 - comparison of Ich metabolic enzymes painted on KEGG pathways with those of T. thermophila, P. tetraurelia and D. rerio. For each pathway, hyperlinks are provided to view the relevant KEGG map painted in red foreground to indicate enzymes present in Ich and green background to indicate enzymes present in other organisms. [file gb-2011-12-10-r100-S16.ZIP › Fig-S5/maps/Phenylalanine.png]

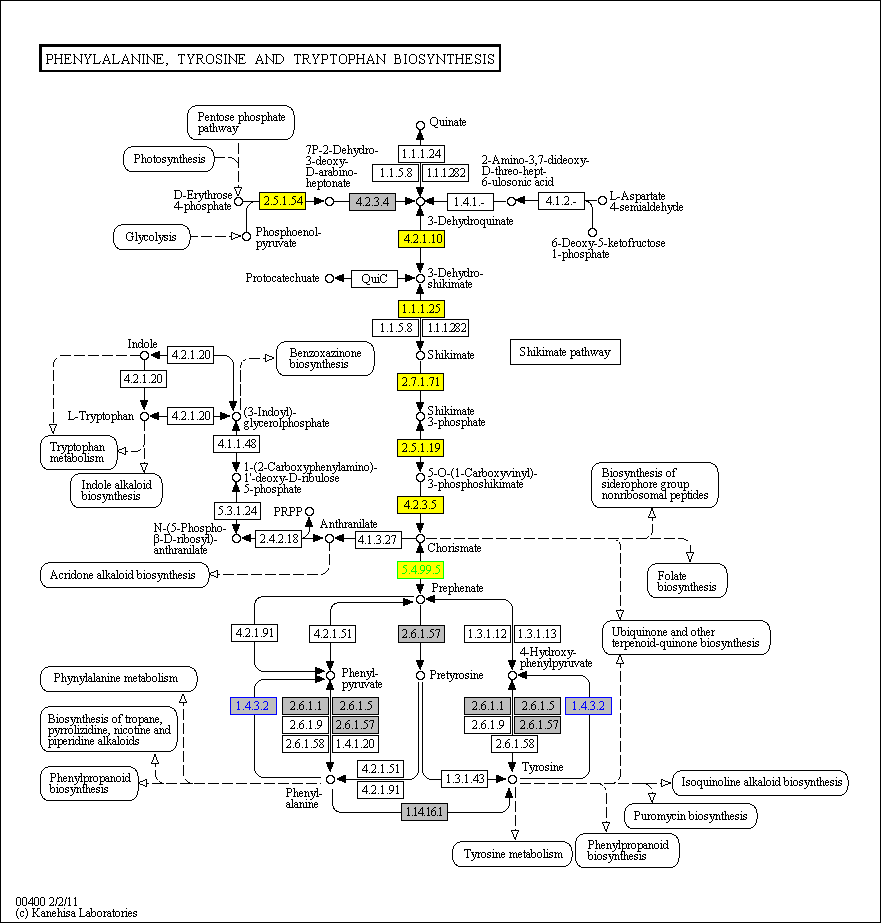

Supplement: Additional file 16 — Figure S5 - comparison of Ich metabolic enzymes painted on KEGG pathways with those of T. thermophila, P. tetraurelia and D. rerio. For each pathway, hyperlinks are provided to view the relevant KEGG map painted in red foreground to indicate enzymes present in Ich and green background to indicate enzymes present in other organisms. [file gb-2011-12-10-r100-S16.ZIP › Fig-S5/maps/Pheylalanine&Tyrosine&Tryptophan-biosynthesis.png]

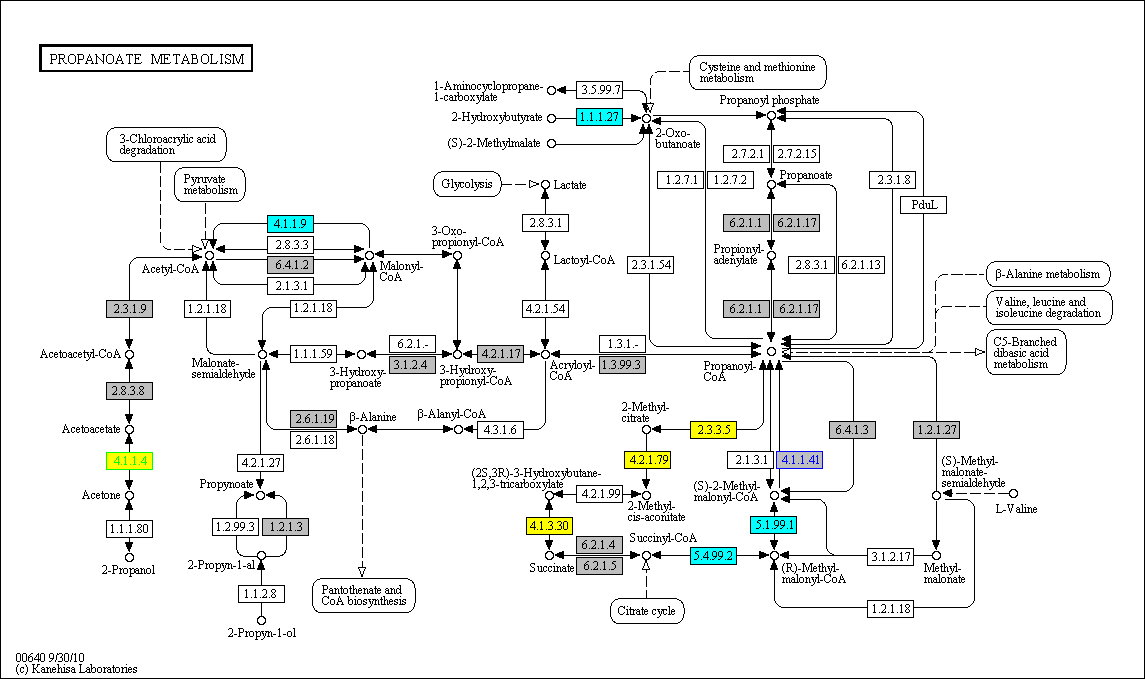

Supplement: Additional file 16 — Figure S5 - comparison of Ich metabolic enzymes painted on KEGG pathways with those of T. thermophila, P. tetraurelia and D. rerio. For each pathway, hyperlinks are provided to view the relevant KEGG map painted in red foreground to indicate enzymes present in Ich and green background to indicate enzymes present in other organisms. [file gb-2011-12-10-r100-S16.ZIP › Fig-S5/maps/Propanoate.png]

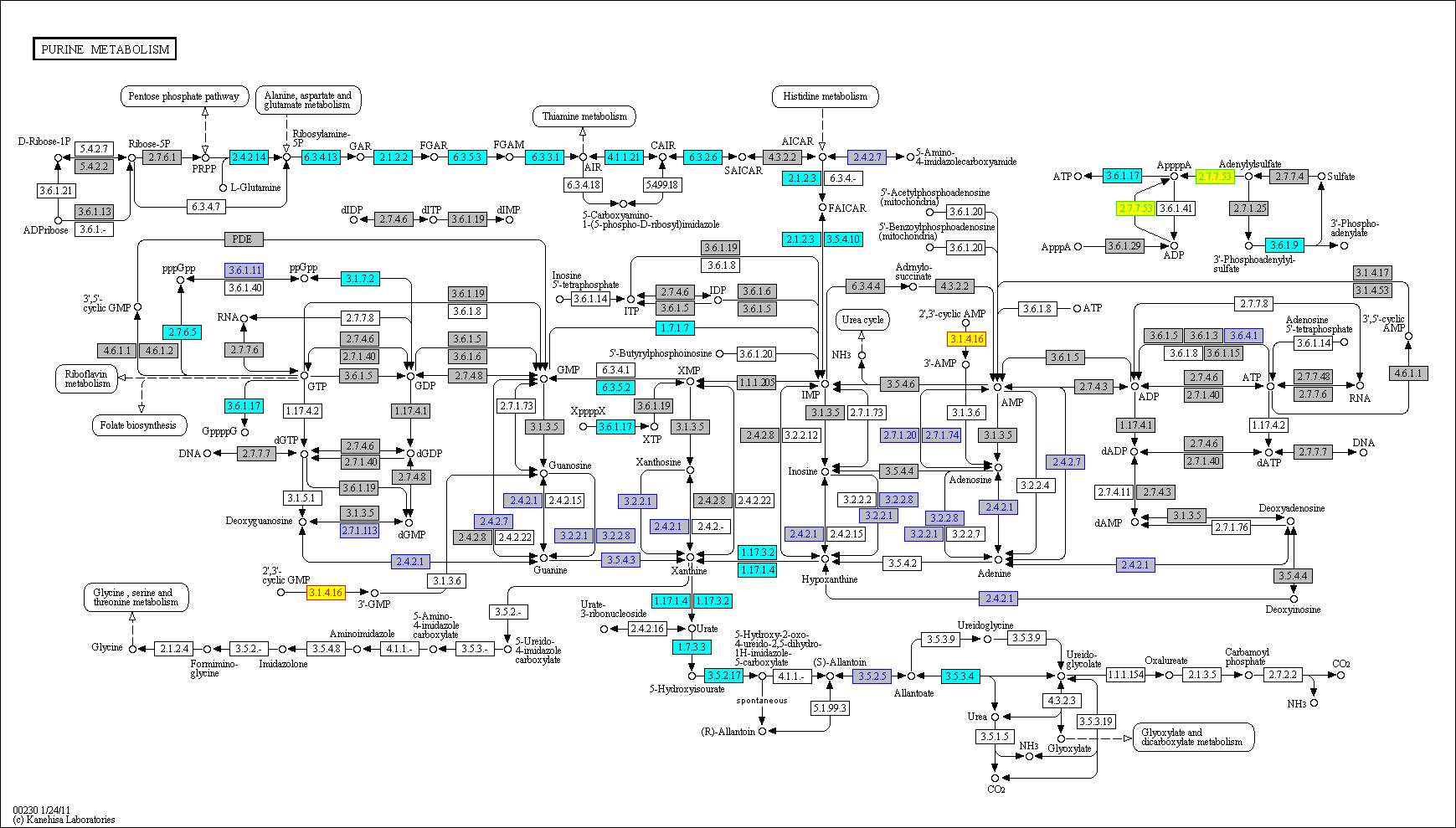

Supplement: Additional file 16 — Figure S5 - comparison of Ich metabolic enzymes painted on KEGG pathways with those of T. thermophila, P. tetraurelia and D. rerio. For each pathway, hyperlinks are provided to view the relevant KEGG map painted in red foreground to indicate enzymes present in Ich and green background to indicate enzymes present in other organisms. [file gb-2011-12-10-r100-S16.ZIP › Fig-S5/maps/purine.png]

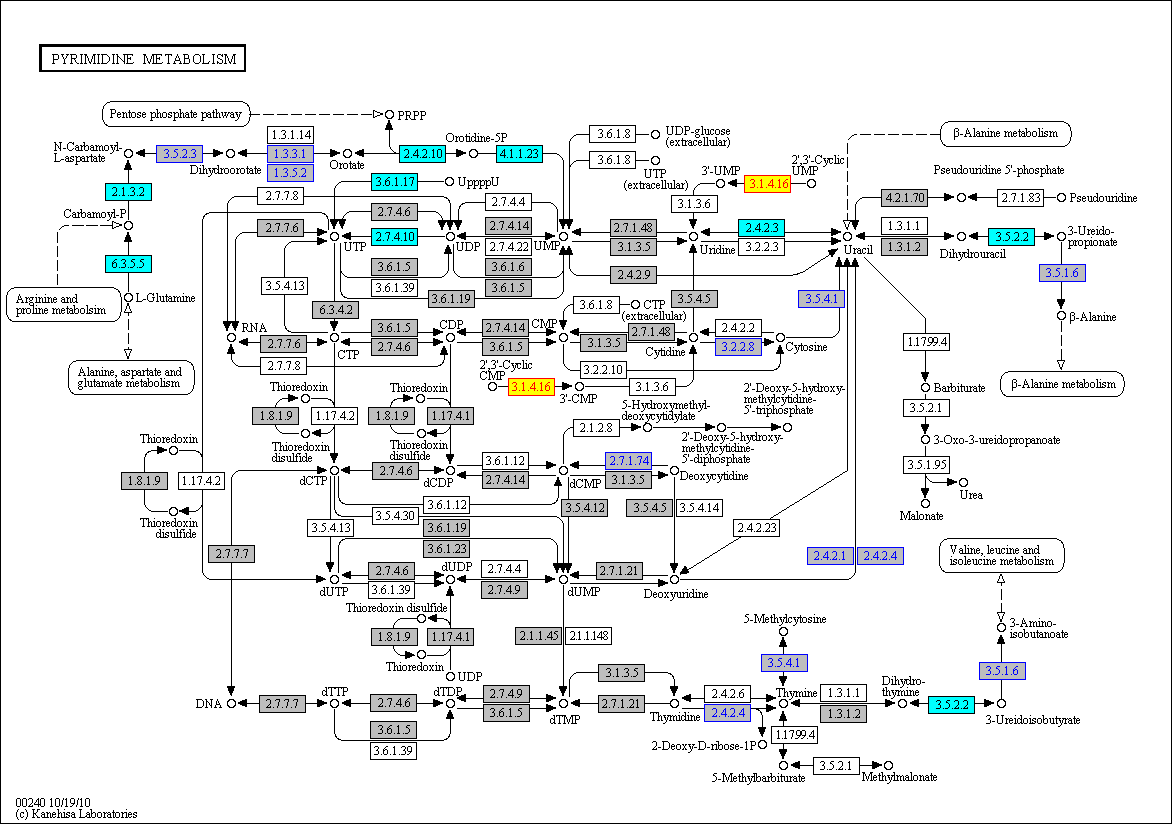

Supplement: Additional file 16 — Figure S5 - comparison of Ich metabolic enzymes painted on KEGG pathways with those of T. thermophila, P. tetraurelia and D. rerio. For each pathway, hyperlinks are provided to view the relevant KEGG map painted in red foreground to indicate enzymes present in Ich and green background to indicate enzymes present in other organisms. [file gb-2011-12-10-r100-S16.ZIP › Fig-S5/maps/pyrimidine.png]

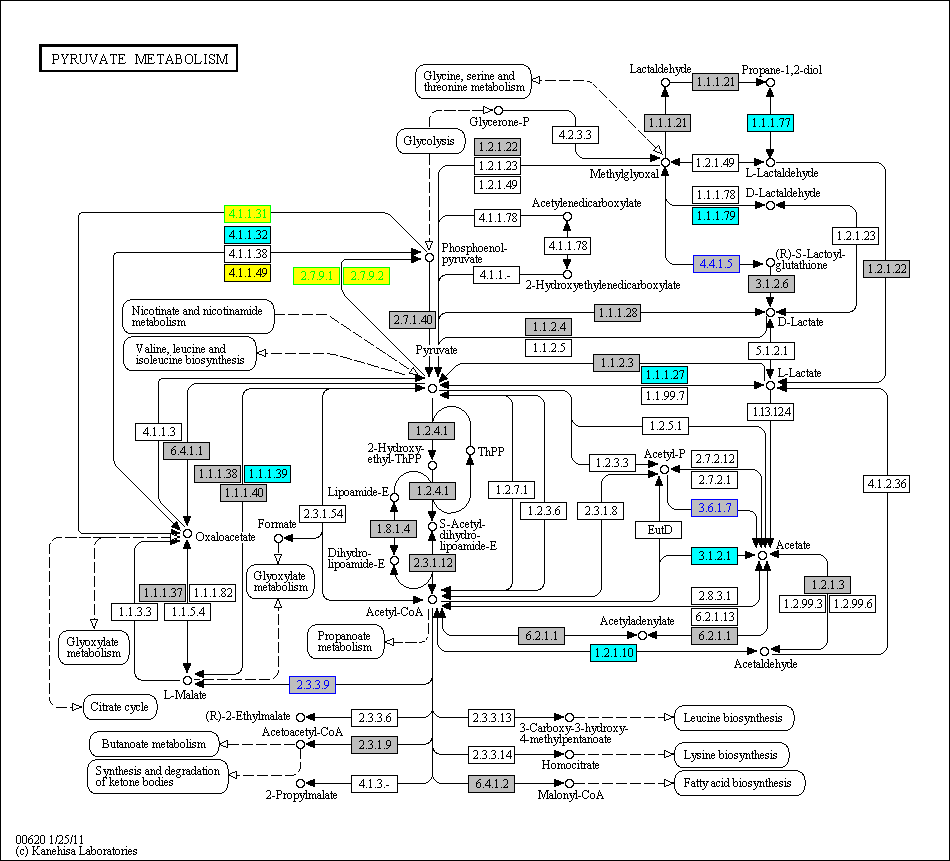

Supplement: Additional file 16 — Figure S5 - comparison of Ich metabolic enzymes painted on KEGG pathways with those of T. thermophila, P. tetraurelia and D. rerio. For each pathway, hyperlinks are provided to view the relevant KEGG map painted in red foreground to indicate enzymes present in Ich and green background to indicate enzymes present in other organisms. [file gb-2011-12-10-r100-S16.ZIP › Fig-S5/maps/Pyruvate.png]

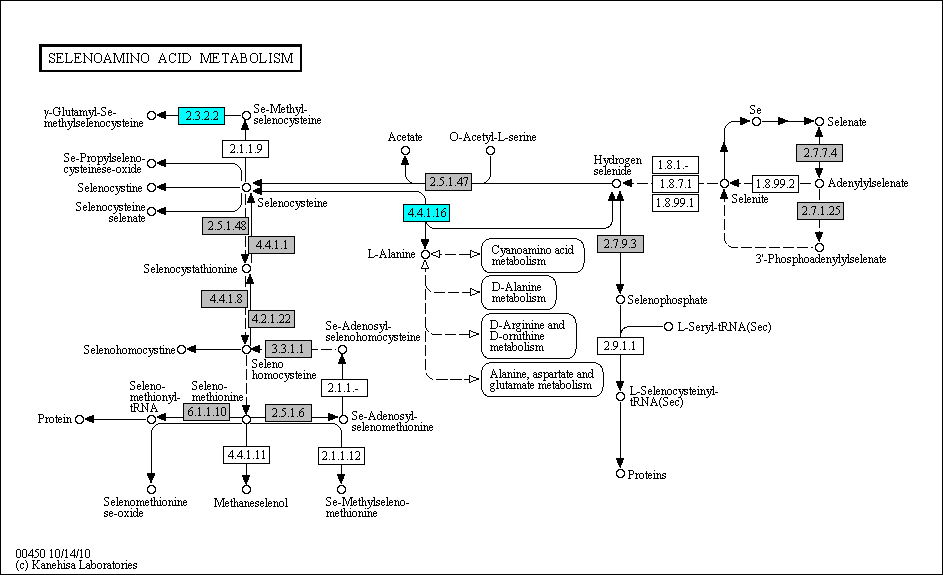

Supplement: Additional file 16 — Figure S5 - comparison of Ich metabolic enzymes painted on KEGG pathways with those of T. thermophila, P. tetraurelia and D. rerio. For each pathway, hyperlinks are provided to view the relevant KEGG map painted in red foreground to indicate enzymes present in Ich and green background to indicate enzymes present in other organisms. [file gb-2011-12-10-r100-S16.ZIP › Fig-S5/maps/Selenoamino-acid.png]

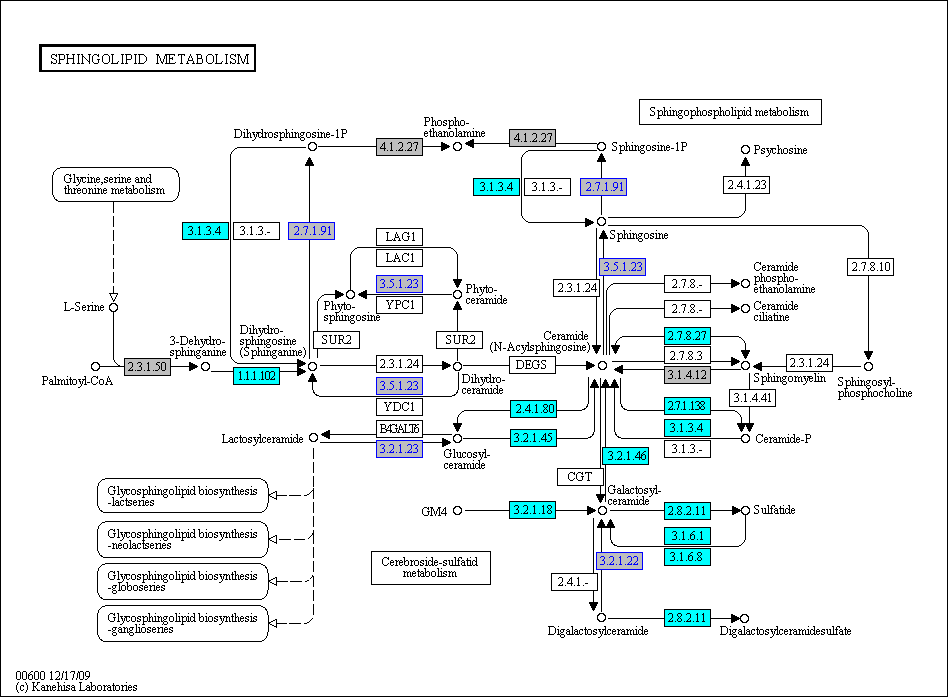

Supplement: Additional file 16 — Figure S5 - comparison of Ich metabolic enzymes painted on KEGG pathways with those of T. thermophila, P. tetraurelia and D. rerio. For each pathway, hyperlinks are provided to view the relevant KEGG map painted in red foreground to indicate enzymes present in Ich and green background to indicate enzymes present in other organisms. [file gb-2011-12-10-r100-S16.ZIP › Fig-S5/maps/Sphingolipid.png]

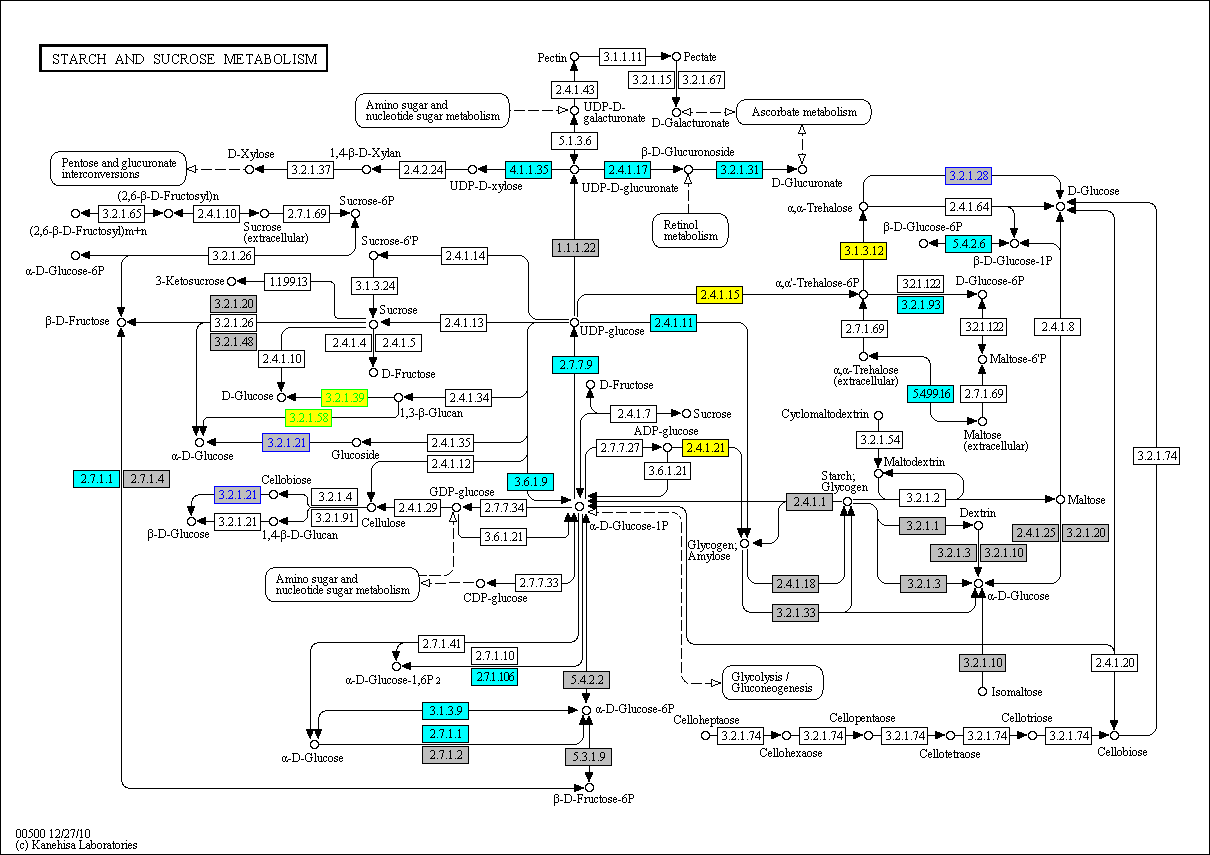

Supplement: Additional file 16 — Figure S5 - comparison of Ich metabolic enzymes painted on KEGG pathways with those of T. thermophila, P. tetraurelia and D. rerio. For each pathway, hyperlinks are provided to view the relevant KEGG map painted in red foreground to indicate enzymes present in Ich and green background to indicate enzymes present in other organisms. [file gb-2011-12-10-r100-S16.ZIP › Fig-S5/maps/Starch&Sucrose.png]

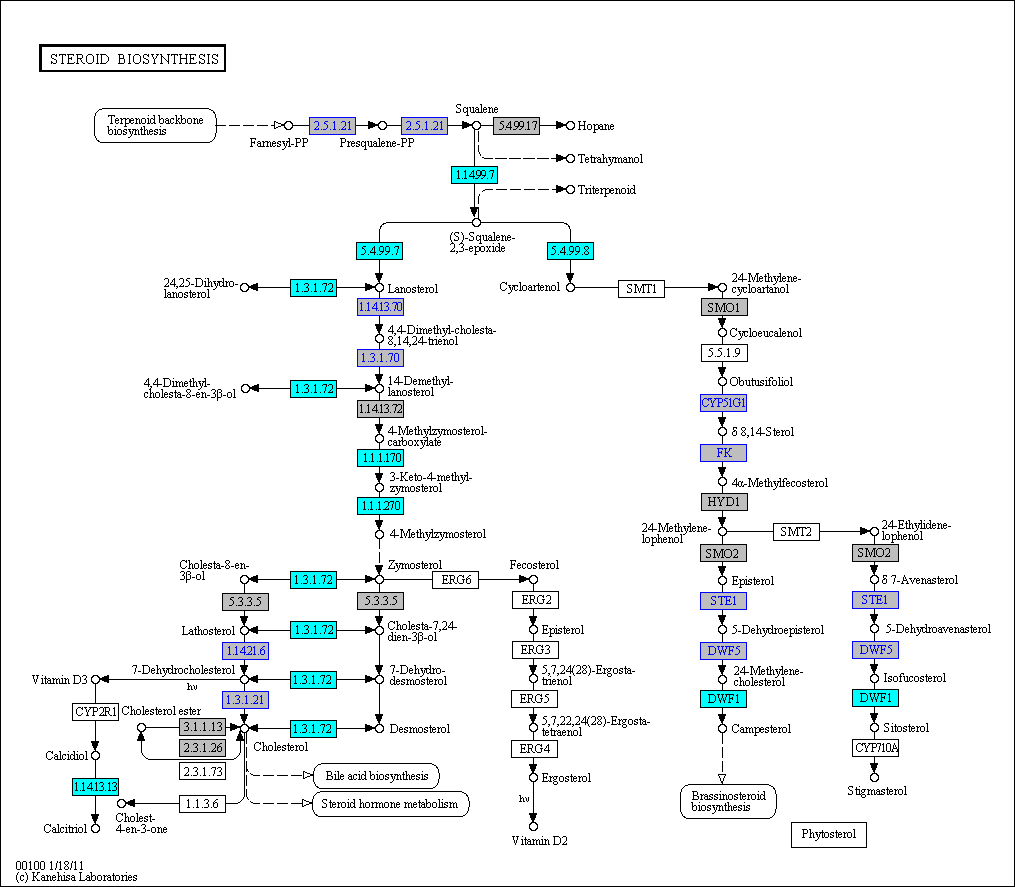

Supplement: Additional file 16 — Figure S5 - comparison of Ich metabolic enzymes painted on KEGG pathways with those of T. thermophila, P. tetraurelia and D. rerio. For each pathway, hyperlinks are provided to view the relevant KEGG map painted in red foreground to indicate enzymes present in Ich and green background to indicate enzymes present in other organisms. [file gb-2011-12-10-r100-S16.ZIP › Fig-S5/maps/Steroid.png]

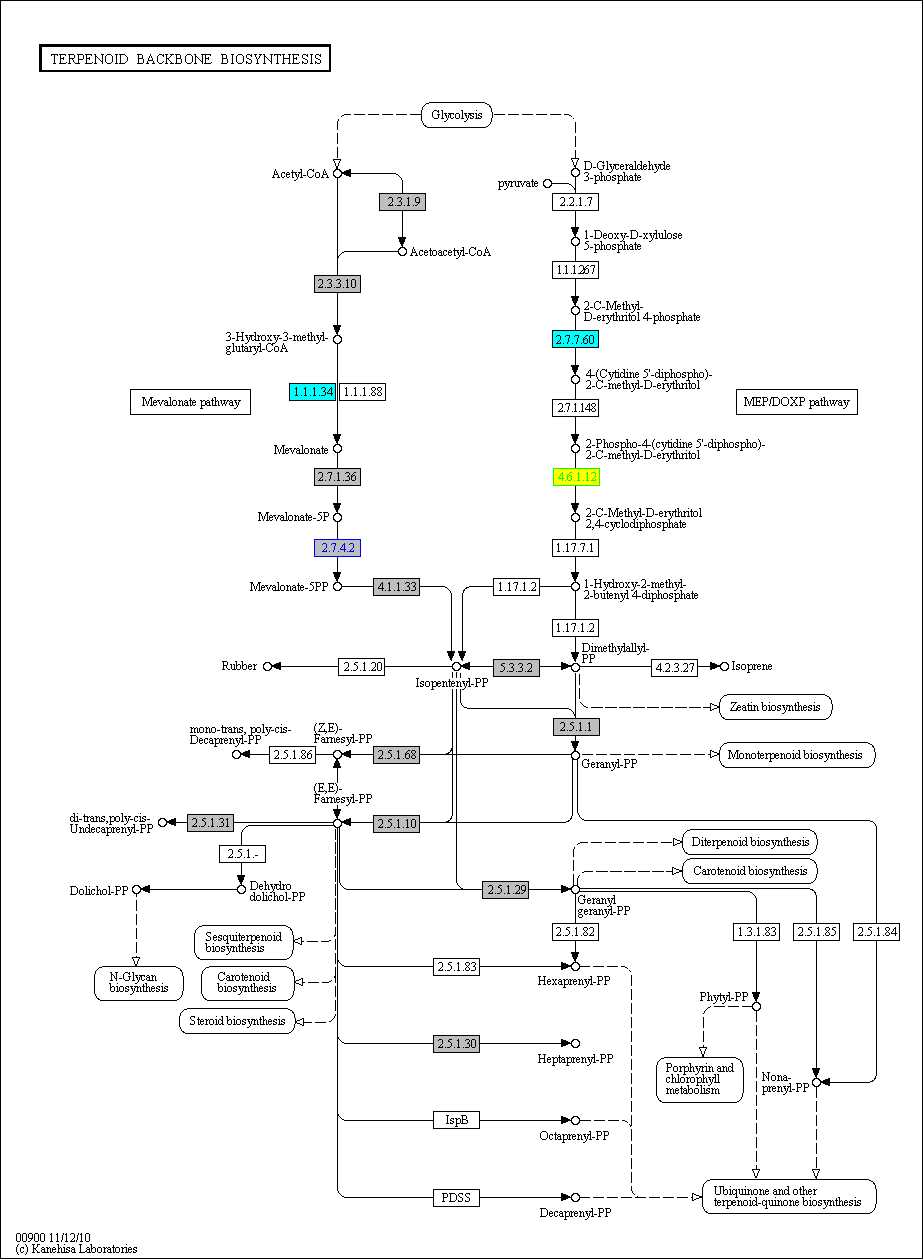

Supplement: Additional file 16 — Figure S5 - comparison of Ich metabolic enzymes painted on KEGG pathways with those of T. thermophila, P. tetraurelia and D. rerio. For each pathway, hyperlinks are provided to view the relevant KEGG map painted in red foreground to indicate enzymes present in Ich and green background to indicate enzymes present in other organisms. [file gb-2011-12-10-r100-S16.ZIP › Fig-S5/maps/Terpenoid_biosynthesis.png]

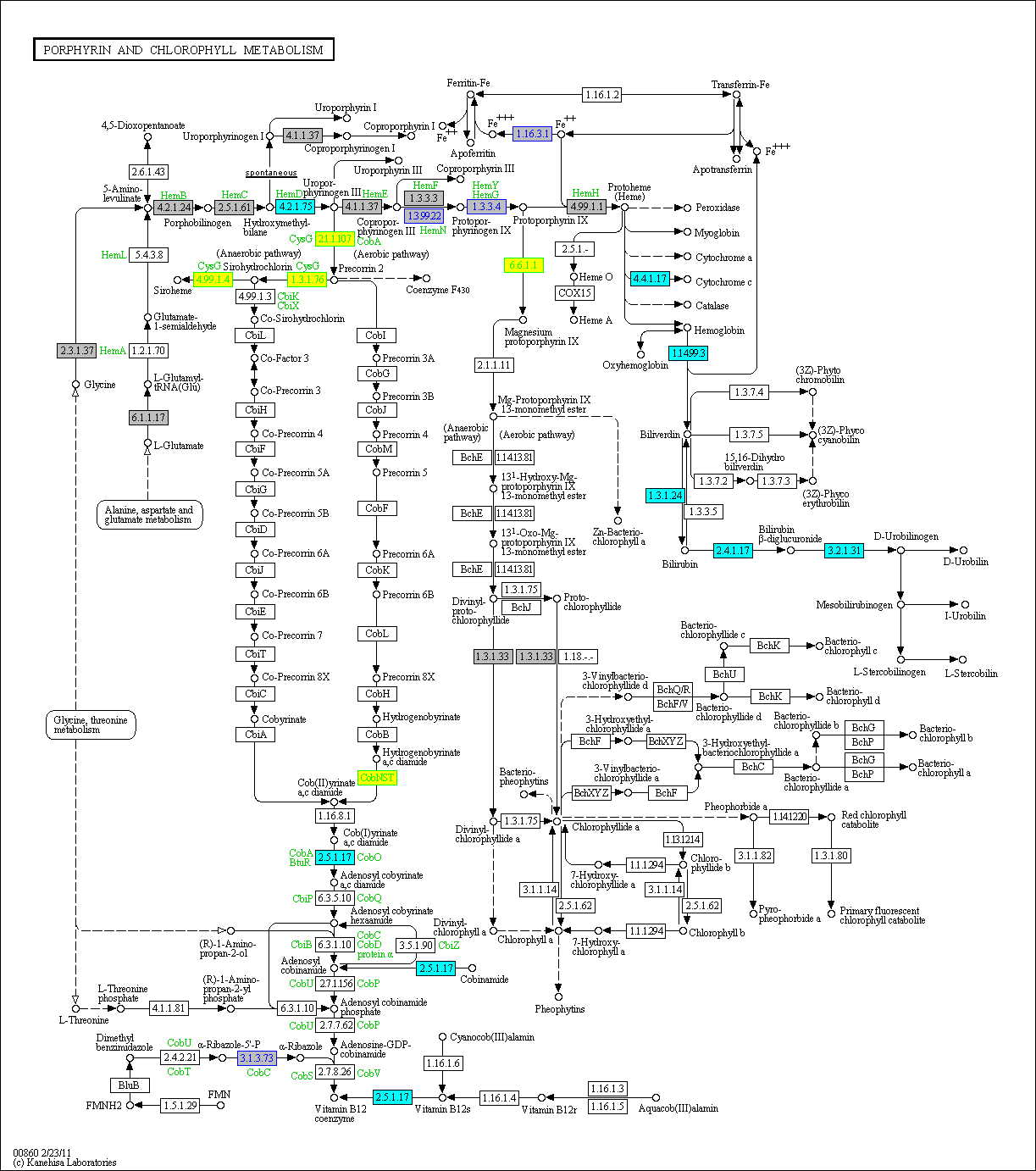

Supplement: Additional file 16 — Figure S5 - comparison of Ich metabolic enzymes painted on KEGG pathways with those of T. thermophila, P. tetraurelia and D. rerio. For each pathway, hyperlinks are provided to view the relevant KEGG map painted in red foreground to indicate enzymes present in Ich and green background to indicate enzymes present in other organisms. [file gb-2011-12-10-r100-S16.ZIP › Fig-S5/maps/Tetrapyrrole.png]

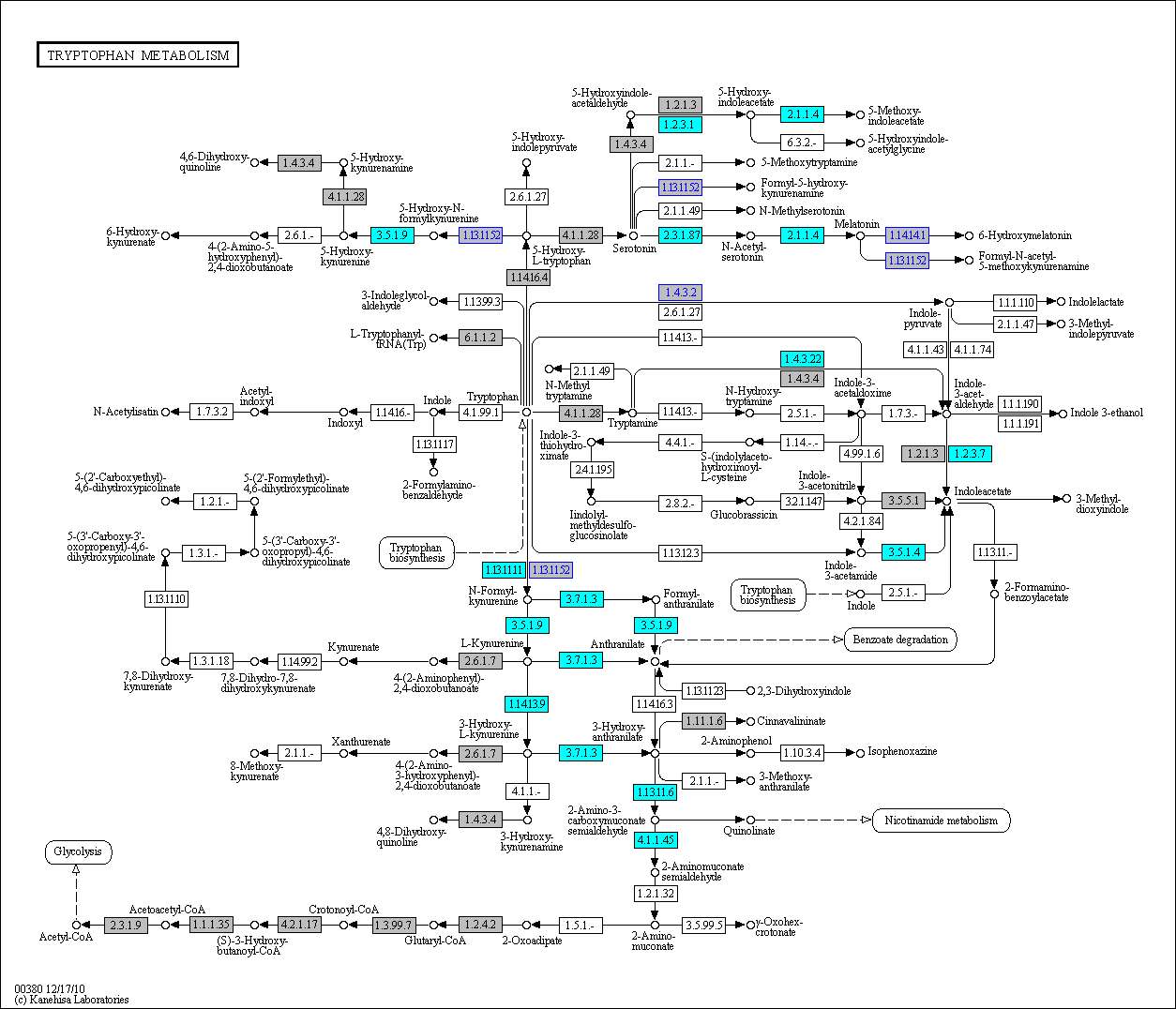

Supplement: Additional file 16 — Figure S5 - comparison of Ich metabolic enzymes painted on KEGG pathways with those of T. thermophila, P. tetraurelia and D. rerio. For each pathway, hyperlinks are provided to view the relevant KEGG map painted in red foreground to indicate enzymes present in Ich and green background to indicate enzymes present in other organisms. [file gb-2011-12-10-r100-S16.ZIP › Fig-S5/maps/Tryptophan.png]

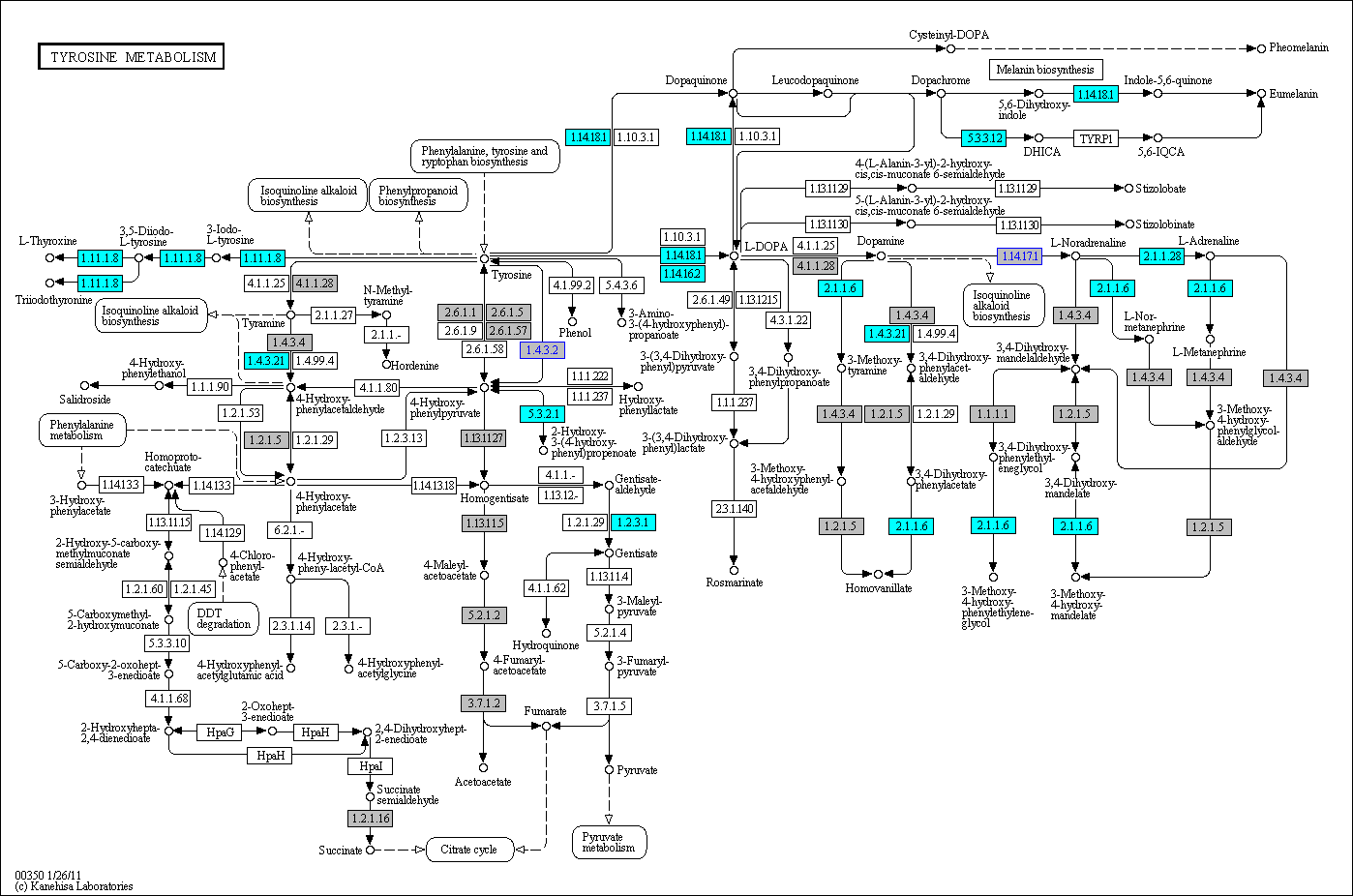

Supplement: Additional file 16 — Figure S5 - comparison of Ich metabolic enzymes painted on KEGG pathways with those of T. thermophila, P. tetraurelia and D. rerio. For each pathway, hyperlinks are provided to view the relevant KEGG map painted in red foreground to indicate enzymes present in Ich and green background to indicate enzymes present in other organisms. [file gb-2011-12-10-r100-S16.ZIP › Fig-S5/maps/tyrosine.png]

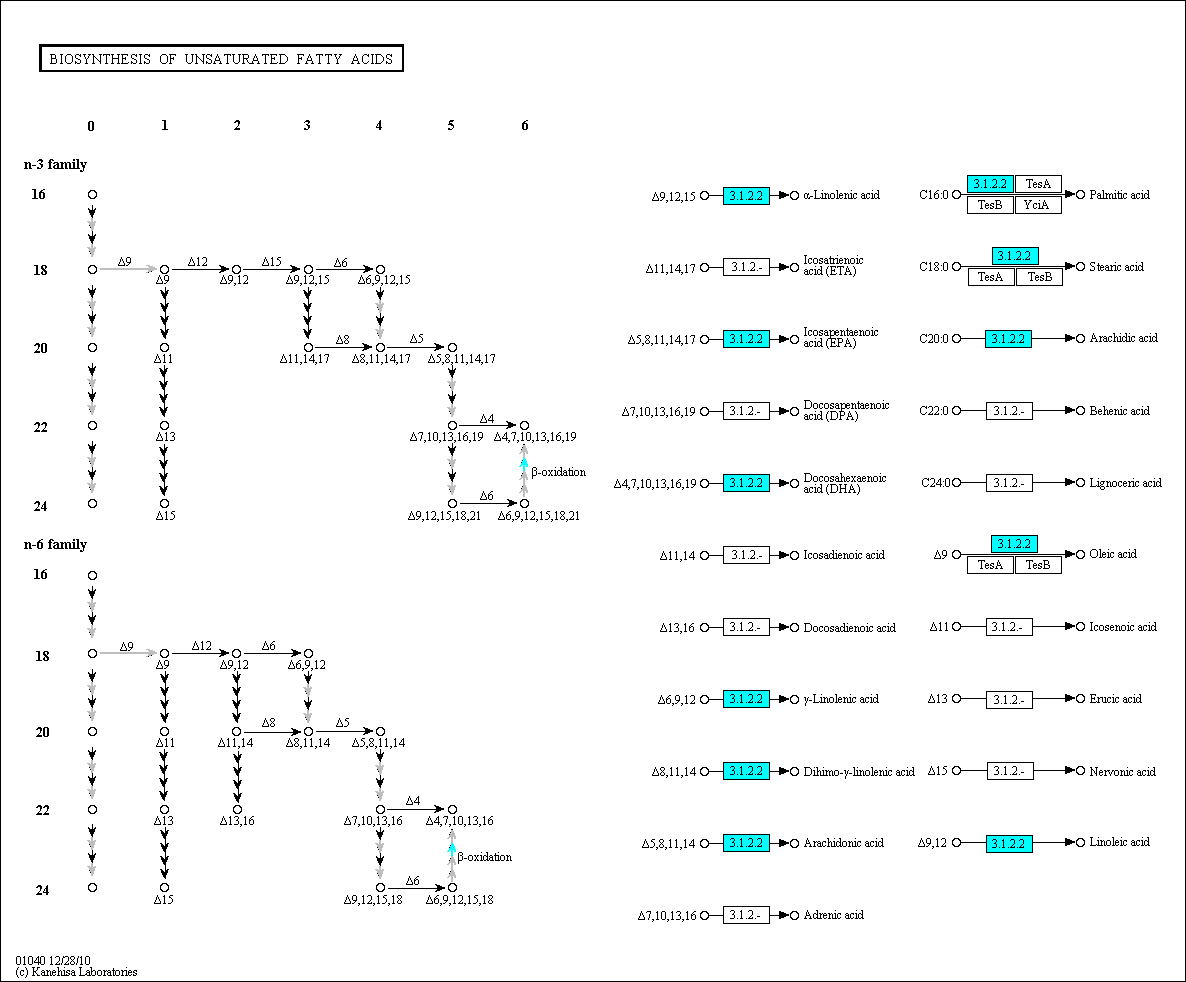

Supplement: Additional file 16 — Figure S5 - comparison of Ich metabolic enzymes painted on KEGG pathways with those of T. thermophila, P. tetraurelia and D. rerio. For each pathway, hyperlinks are provided to view the relevant KEGG map painted in red foreground to indicate enzymes present in Ich and green background to indicate enzymes present in other organisms. [file gb-2011-12-10-r100-S16.ZIP › Fig-S5/maps/Unsaturated-FA_biosynthesis.png]

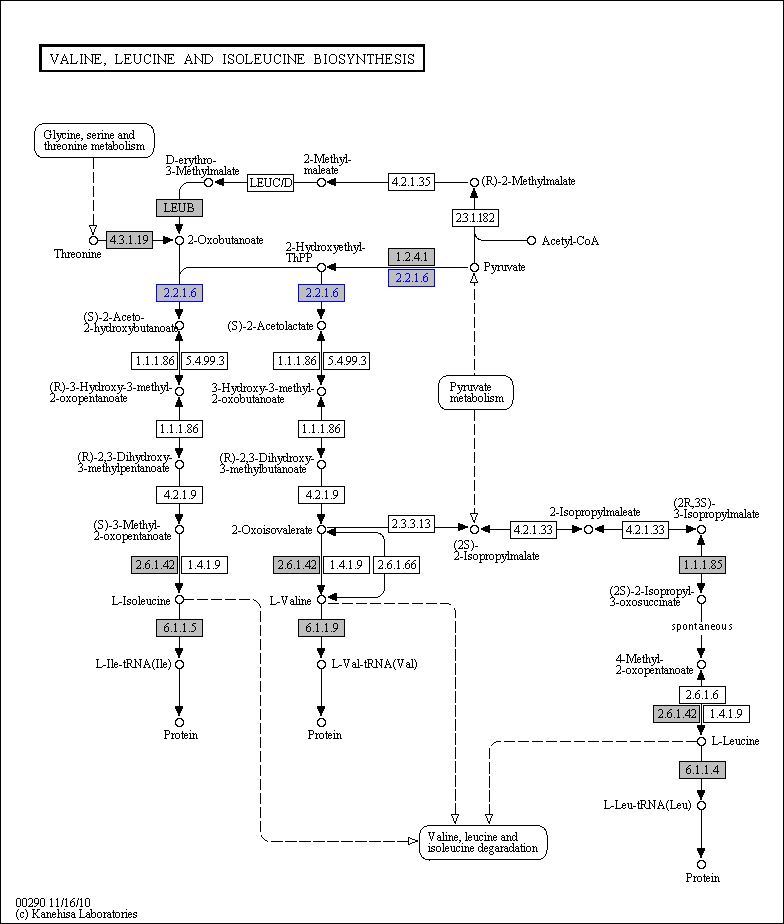

Supplement: Additional file 16 — Figure S5 - comparison of Ich metabolic enzymes painted on KEGG pathways with those of T. thermophila, P. tetraurelia and D. rerio. For each pathway, hyperlinks are provided to view the relevant KEGG map painted in red foreground to indicate enzymes present in Ich and green background to indicate enzymes present in other organisms. [file gb-2011-12-10-r100-S16.ZIP › Fig-S5/maps/Valine&Leucine&Isoleucine-biosynthesis.png]

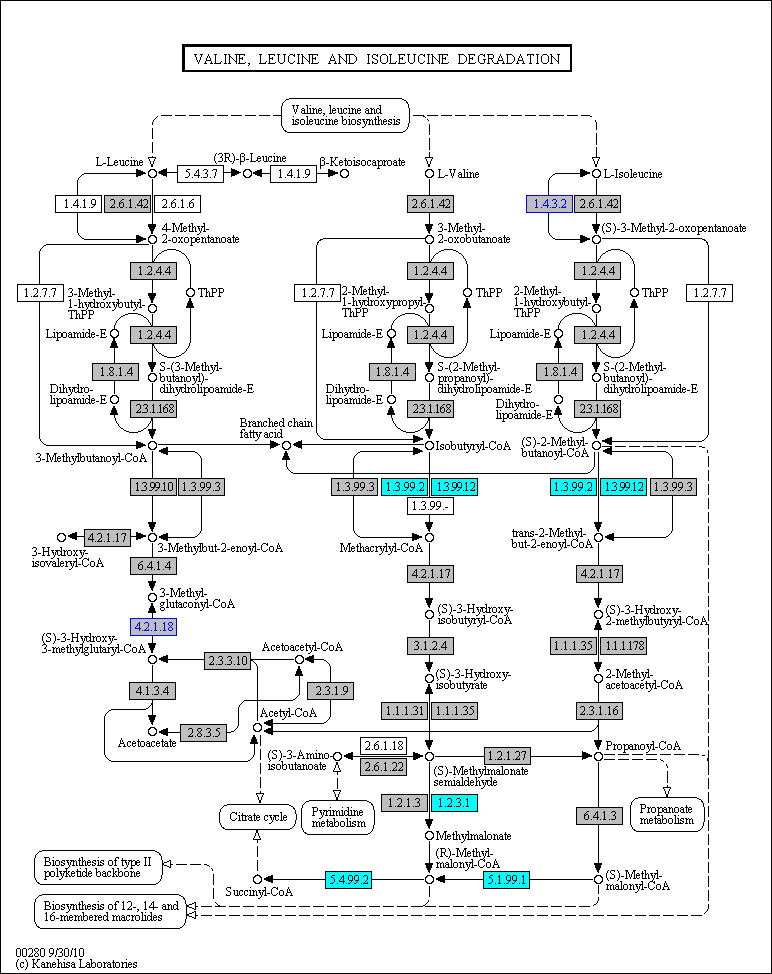

Supplement: Additional file 16 — Figure S5 - comparison of Ich metabolic enzymes painted on KEGG pathways with those of T. thermophila, P. tetraurelia and D. rerio. For each pathway, hyperlinks are provided to view the relevant KEGG map painted in red foreground to indicate enzymes present in Ich and green background to indicate enzymes present in other organisms. [file gb-2011-12-10-r100-S16.ZIP › Fig-S5/maps/Valine&Leucine&Isoleucine-degradation.png]
