# Supplementary material for: Comparative genomics of the pathogenic ciliate Ichthyophthirius multifiliis, its free-living relatives and a host species provide insights into adoption of a parasitic lifestyle and prospects for disease control
Source: Genome Biol. 2011 Oct 17;12(10):R100. doi: 10.1186/gb-2011-12-10-r100 (PMC3341644; doi:10.1186/gb-2011-12-10-r100)
Supplement: Additional file 4 — Table S3 - correspondence of predicted genes for ATP synthase subunits of T. thermophila and Ich. [file gb-2011-12-10-r100-S4.DOCX]

| **T. thermophila gene product** | **Ich gene product(s)** | **Genbank Accession** | **%ID^a^** |
| --- | --- | --- | --- |
|  |  |  |  |
| TTHERM_00571860 F_1_ α subunit | **2533.m000153** | IMG5_124890 | 85% |
| TTHERM_00571860 F_1_ α subunit | **2338.m000163** | IMG5_092580 | 85% |
| TTHERM_00585260 F_1_ β subunit | **2407.m000171** | IMG5_103590 | 90% |
| TTHERM_00585260 F_1_ β subunit | **2671.m000128** | IMG5_145220 | 89% |
| THERM_00118610 F_1_ γ subunit | **2354.m000074** | IMG5_094520 | 73% |
| TTHERM_00684790 F_O_ OSCP subunit | **2862.m000025** | IMG5_180630 | 68% |
| TTHERM_00765280 BCAT-like subunit | **2518.m000096** | IMG5_122050 | 68% |
| Ymf66 (ATP synthase F_O_ subunit a-like protein; product of TepyoMp02) | **2995.m00075** (**ymf66**) | IMG5_M206945 (ymf66) | 50% |
| ATP synthase F_O_ subunit c (product of TepyoMp18) | **2995.m00077** (**atp9**) | IMG5_M206962 (atp9) | 79% |
| TTHERM_00137990 | **2964.m000048** | IMG5_200230 | 67% |
| TTHERM_00145440 | **2608.m000052b** | IMG5_136670b | 71% |
| TTHERM_00127260 F_O_ b subunit candidate | **2138.m000094** | IMG5_067880 | 66% |
| TTHERM_00529760 Fo b subunit candidate | **2496.m000116** | IMG5_117260 | 72% |
| TTHERM_00823660 Fo b subunit candidate | **2573.m000131** | IMG5_131360 | 66% |
| TTHERM_01188360_revised^b^ putative ATP synthase F_O_ d subunit | **2574.m000038** | IMG5_131610 | 74% |
| TTHERM_00486310 | **2041.m000060** | IMG5_054710 | 79% |
| TTHERM_00649060 | **2696.m000050** | IMG5_149900 | 68% |
| TTHERM_00289380 | **2893.m000121** | IMG5_186670 | 73% |
| TTHERM_01528510 | **2676.m000106** | IMG5_146230 | 61% |
| TTHERM_00316290 | **2089.m000167** | IMG5_061210 | 83% |
| TTHERM_00825290 | **2768.m000067** | IMG5_163230 | 54% |
| TTHERM_00571670 | **2041.m000062** | IMG5_054730 | 69% |
| TTHERM_00068120_revised^b^ | **2810.m000063a** | IMG5_172140a | 62% |
| TTHERM_01094890_revised^b^ Putative F_1_ delta subunit | **(EST data)** | (EST data) | ~72% |

^a^as calculated by NCBI BLASTp

^b^translated from revised *T. thermophila* gene models as published in the supplementary materials of [1]

1. Balabaskaran Nina P, Dudkina NV, Kane LA, van Eyk JE, Boekema EJ, et al. (2010) Highly divergent mitochondrial ATP synthase complexes in Tetrahymena thermophila. PLoS Biol 8: e1000418.
