# Supplementary material for: Comparative genomics of the pathogenic ciliate Ichthyophthirius multifiliis, its free-living relatives and a host species provide insights into adoption of a parasitic lifestyle and prospects for disease control
Source: Genome Biol. 2011 Oct 17;12(10):R100. doi: 10.1186/gb-2011-12-10-r100 (PMC3341644; doi:10.1186/gb-2011-12-10-r100)
Supplement: Additional file 10 — Figure S3 - multiple sequence alignment of Ich immobilization antigen peptide sequences. Alignment was generated using MUSCLE [126] and edited by hand. Conserved cysteine residues are enclosed in red rectangles. Hydrophobic regions at the amino and hydroxyl termini are shown with yellow highlighting. [file gb-2011-12-10-r100-S10.PDF]

i-antigens_locus_wrap.msf

```
IMG5_106840A    1 ..........MKQIKILQKKMHKNIIITLIISLFINKLKATKCIIGTETNIAGSDDDRGDRANCVNCKANYYFSG..........ANFISGVS....QCFQCPSQ......KTVDAQAN.............
IMG5_069260A    1 ....................MYKNILITLIFSLFLTELKATLCPNGTETNVANQRDDGGNPTNCVNCRVNFYFNGV........PATFNPGVS....QCMPCIDI......KTEDAQAN.............
IMG5_069230A    1 ....................MNKNILITVIISLFFNELKAADCPYGTETDIAGQRNNRGNIANCVNCRANYYFNGS........SANFIPGVS....QCGRCTYS......KTVGAQAN.............
IMG5_069240A    1 ....................MNKNILITVIISLFFNELKAANCPYGTETDIAGQRNDRGNIANCVNCRANYYFNGS........SADFIPGVS....QCGRCTYS......KTVGAQAN.............
IMG5_002150A    1 ....................MKFNILIILIISLFINELRAVNCPNGAAIANGQSDTGAADINTCTHCQKHFYFNGGNPAGQAPGAAQFNPGVS....QCIACQVHK.ADSQHRQGGDANLAAQCSNLCPAGT
IMG5_203550A    1 ....................MKYNILIILIISLFINELRALNCPAGTQTQAGLFEDG..DLANCTHCRANFYYNGGNPAGDAVGAQAFTPGAANATGQCVACQVNR.EGSAGTQGAAANLATQCSVACPAGT
IMG5_203560A    1 ....................MS..ILIIIIKNIYIQIKNNLLYHIYYILLKIK.SNKIANIKINFNFKNQFVFYLKR..KIYLFIHKFFSFIN......Y..LIF......KKKRDQKMK............
IMG5_080670A    1 ....................MSNNLLILLIITLFINELNALACPVGTQTAAGAQQDD..AIINCNHCKANFYYKS.......VDGTPFNAGAS....VCTPCSKNK.AADDVPPPAAA..............
IMG5_069270A    1 ....................MKNNILVILIISLFINQIKSANCPVGTETNTAGQVDDLGNPANCVNCQKNFYYNN.........AAAFVPGAS....TCTPCPQKKDAGAQPNPPATANLVTQCNVKCPAGT
IMG5_106820A    1 .........MQQIKILLKKKMHKIILLSLIISLFINKLSFLPCPEGTETNVSGVDDDQGNPANCVNCQPDYYFNG..........DNFIPGVS....ECEECQIS......KAEGTQAN.............
IMG5_190880A    1 MTKLTIIYNKINHNFIKNKKCKIIILISLIILLFINNLKSQTCLQGTETNVAGEDNSLGDLANCVNCKPDYYFNG..........DNFIPGVS....ECEECPIP......KFEDGQAN.............
IMG5_190870A    1 ....................MQKIILISLIILLFINNLKSQTCLQGTETNVAGEDDSLGDLANCVNCKPDYYFNG..........NNFIPGVS....ECEECPIP......NLEGGQAN.............
IMG5_018010A    1 ....................MQKIIQISLIISLFINQFNFLPCPEGTETNVAGIDDDSGNPANCVNCKIDYYFNG..........DNFIPGVS....VCKECQIL......KYEGEQAN.............
IMG5_190900A    1 ....................MKQSFLISLIILLFINKFNFLPCPKGTETNVSGDDDNQGNPVNCVNCKPDYYYNG..........DNFDPGVS....ECEVCPIP......KQEDAIAN.............
IMG5_106810A    1 ....................M....ILKQIFNLFN.YLITQLCLLGTETNVAGEKDHQRNPANCVNFKPDYYFNG..........DNFIPGVS....ECQQCPIPL.....KIAAQQAN.............
IMG5_106830A    1 ....................MHKNILIILIIQLFINKLKAVKCNWGTETNLEGIDDNLGNRANCVNCKANYYFSG..........ANFIPGVS....LCERCPIS......KFQDARAN.............
IMG5_190890A    1 ....................MYKYFLITLVRSLFINELIAIKCNIGTETNIAGDDDNRGDIANCVNCRANYYYNG..........DNFIPGVS....QCEKCPIS......KKVGARPN.............

IMG5_106840A   90 ...............................................................................AGRIATLAIQCNTDCPTGTQTEQGILLMYYK..EKNVIFVRLTIIS.......
IMG5_069260A   82 ...............................................................................SGRIATLTLQCKSDCPDGTQTALGNTNYLAQ..RQECSFCQANHYFYSEIN..
IMG5_069230A   82 ...............................................................................PGRIATLALQCDDNCPDGTQTALGKTKYVAQ..KQECSFCKANYYYETTKDF.
IMG5_069240A   82 ...............................................................................PGRIATLALQCDDNCPDGTQTALGKTKYVAQ..KQECSFCKANYYYETTKDF.
IMG5_002150A  108 AVEDGQPT.FTQSLSQCVNCKANFYFNGGNPTGQAPGAGQFDPTQLIVNPDNNPEVPNVSGPNGQCVACQVNKSDSQLRPGAQANLATQCNNECPTGTAIQDGAIFIYTQS.ISQCTFCKVDFYFNGGNPSA
IMG5_203550A  110 AVADGQTLNFALSVAQCTHCRANFYYNGGNPAGDAVGAQAFTP..............GAANATGQCVACQVNREGSVGTQGAAANLATQCSVACPAGTAVADGQTLNFALS.VTQCTHCRANFYYNGGNPAG
IMG5_203560A   82 ...........................................................................YLIIFISLLFINQLRAVKCPAGSQTDAGSDQVAAENNLPECTKCKLNFYYAGASP..
IMG5_080670A   85 ...............................................................................LGADATIVNQCNVSCPAGTTIAN.QSTNYVNA.ATECIKCAANFYYSGPN...
IMG5_069270A  100 AIAGGATD.YAAIITECVNCRINFYNENAP...........................NFNAGASTCTACPVNRVGGALNAGNAATIVAQCNVACPTGTALDDGVTTDYVRS.FTECVKCRVNFYYNGNN...
IMG5_106820A   91 ...............................................................................PGGIATLALQCDINCPAGTQTENGETVYVAQ..KQECNFCKADHYNIFDKD..
IMG5_190880A  100 ...............................................................................AGGISRLAVQCDINCPDDTQTENGDTVYVSQ..KEEYSFCQADYFFIIDEI..
IMG5_190870A   80 ...............................................................................EGGIATLAVQCDINCPDGTQTENGDTFYIAQ..KEECSFCQADYFFIIDEI..
IMG5_018010A   80 ...............................................................................PGGIATLAVQCDINCPYGTQTENGQTFYIAQ..KQECNFCKADYYYFIEIN..
IMG5_190900A   80 ...............................................................................PARFAVLSVQCDINCPYGTQTEHGQTVYVQQ..AEECNFCKDNHFYEFFGL..
IMG5_106810A   76 ...............................................................................AGGIAKLALQCEINCPAGTQTEHGETVYVAQ..KEECNFCKAGFYFQSDIF..
IMG5_106830A   80 ...............................................................................DGNIATLAVQCDINCPTGTQTEQGNTTYVLL..RNECNFCKANYYYESFII..
IMG5_190890A   80 ...............................................................................AGGNARLVLQCDINCPTGTQTEQGNTTYVAQ..REEYNFCKVNHYYQTKGL..

IMG5_106840A  134 ................................................................................................................KIIQCFFHQVLANVINVQYK
IMG5_069260A  131 ......VFRAGG...........................................................................DGCDACPVR..KTSGAEAIAGINASLAQQCDVTCPDGTVTATGST
IMG5_069230A  132 ......VFLLGF...........................................................................SRCDVCPVK..KASGSQATAGTNASIVKQCDVACPAGTATAYGAT
IMG5_069240A  132 ......VFLLGF...........................................................................SRCDVCPVK..KASGSQATAGTNASIVKQCDVACPAGTATAYGAT
IMG5_002150A  238 QNPGNGQFTPGQLIVN.................PDAATVAQIPMVPGPN......................................SKCVACES...KKTNSQSRSGLEANLAAQCGTECPAGTLVTDGVT
IMG5_203550A  227 DAVGAQAFTPGAANATGQCVACQVNREGSVGTQGAAANLATQCSVACPAGTAVADGQTLNFALSVTQCTHCRANFYYNGDANFVAGTNQCTACPVGANKGADAAAAQGNAAKIKTQCNVLCPTGTSNANGDT
IMG5_203560A  137 ............................................................................................................................LAAGNAVN
IMG5_080670A  133 ......AFIAGTSICT.............................PCPE......................................NGKKDFQL.......AISNKGDNATINIQCNVQCPYGTVSATGEY
IMG5_069270A  200 ...GNTPFNPGKSQCTPCPAIKPAN..VAQATLGNDATITAQCNVACPDGTISAAG.VNNWVAQNTECTNCAPNFYNNNVPNFNPGNSTCLPCPAN..KDYGAEATAGGAATLAKQCNIACPDGTAIASGAT
IMG5_106820A  140 ......DFEPGD...........................................................................YFCEECSIK..KTSGSQATIGFEAQIEKQCNIACPTGTTTETAET
IMG5_190880A  149 ......DFQPGE...........................................................................SVCYKCSIN..KASGSQATLGSEASIEKQCNVVCPTGTTTEIGDS
IMG5_190870A  129 ......DFQPGE...........................................................................SVCYQCSIN..KASGSQATLGSEATSEKQCNVVCPTGTTTEIGDS
IMG5_018010A  129 ......DFEPGD...........................................................................SYCDICQIK..KTSGSLATIGYEATSEKQCIVTCPTGTVSMHGET
IMG5_190900A  129 ......PFEPGM...........................................................................SHCEECSIK..KVNGSLSALGSDALQYKQCITTCPTGTVTYDGVT
IMG5_106810A  125 ......TFSAGV...........................................................................HRCEQCGIK..KAGGSKATLGVNASIEQQCNINCPTGTITDTGAT
IMG5_106830A  129 ......NFTGGV...........................................................................DKCNKCPVQ..KASGFEATLGSNAKLAIQCDVTCPTGTTTKTGST
IMG5_190890A  129 ......FFQPGV...........................................................................DSCNKCSVQ..KTSDSEATLGRNASLAKQCDVSCPTGTSTKTGST

IMG5_106840A  154 KLLVLKQLQDLMQNQPYNVTLLALPE...............QQYKLDKLVMYYLNMNVLITAMLTFILIQLDLFQVQMNVNLVQQKKRFYSNKRFRCFYGCIMLC...........................
IMG5_069260A  180 .SFVQDRIECVNC............................AKHNKADLAKIDLQCEIKCPAGTVLLNGITTNFSNNVSECVKCAANFYTTKQNGWVAGTDICVPCSNILTSGAEANFPATANQEKQCRITG
IMG5_069230A  181 .SYVQNKNECTYCDANFYYKYTNFNPGYSTCSACPIKKTLDAKHTQGDNAKIDVQCDVACPVGTVTFNGKTTNFENDKSECVKCAANFYTRKQNGWIAGTDICIPCPNILISGAQANYPIFASQAKQCKV..
IMG5_069240A  181 .SYVQNKNECTYCDANFYYKYTNFNPGYSTCSACPIKKTLDAKHTQGDNAKIDVQCDVACPVGTVTFNGKTTNFENDKSECVKCAANFYTRKQNGWIAGTDICIPCPNILISGAQANYPMFASQAKQCKV..
IMG5_002150A  312 PTYTVSLSQCVNCKAGFYQN.SNFEAGKSQCNKCAVSKTG.SASVPGNSATSATQCQNDCPAGTVVDDGTSTNFVALASECTKCQANFYASKTSGFAAGTDTCTECSKKLTSGATAKLYAEATQKAQCASS.
IMG5_203550A  359 .FWTAAITDCVNCAANFYFN.GAFNAGTSQCIQCPVSKAN.PATAPGGSASLSTQCNSACPTGTVLDDGTTTNYTTQANQCTKCAVNFFASKTTGFVAGTDTCTECTKKLTSGATAKVFAEATQKIQCAAT.
IMG5_203560A  145 .......GICTQCPQN......................KIDSVATLGSEATIAKQCDISCPPGTVLDDGVIQNYVKSPAECTKCLPNYFQSVENNQIVEAKTCAECSYKLPSGAQARIITEATRKVQCGVA.
IMG5_080670A  185 .YWVQAKTDCVNCDVNFYFIGDAFIAGTNKCLECPSNKAQ.GVATAGSIATIILQCSLDCPDGTVLNDGKTTKYVADSKECVKCAANYYNQKRLGWLAGTDICIACNIKLTSGATAKLPSDATQATQQIFR.
IMG5_069270A  324 .NYVALQTECLNCAANFYFDGNNFQAGSSRCKACPANKVQGAVATAGGTATLIAQCALECPAGTVLTDGTTSTYKQAASECVKCAANFYTTKQTDWVAGIDTCTSCNKKLTSGAEANLPESAKKSIQCDFA.
IMG5_106820A  189 .NYKIHENECINCNVNFYFDSNEFLDGVSTCKPCPVKKIIGAVRTQ.......VQCDVQCPDGTVVKDGKTT.FENEKSECVKCAPNFYTAKQEEWIAGIDICFPCSNILIQGAQANYPSVATQATQCRG..
IMG5_190880A  198 .NYQYHKNECVYCNSNFYFDSNSFLAGTSTCKPYPVNKVVGAIHTLGQNAKKDVQCDVKCPAGTVVKDGKTT.FENDKSECVKCAPNFYTAKPKSWVAGIDKCSPCPNILIQGAQANYPASA...TQCRV..
IMG5_190870A  178 .NYQYEETECVYCNSNFYFDSNDFLAGTSTCKPCPVKKIVGSIHTVGSNAKKDVQCEVKCPAGTVVQDGKAT.FENDKSECVKCAPNFYTAKPQSWVAGIDKCFPCPNILIQGAQANYPASASLATQCRV..
IMG5_018010A  178 .NFYNEKKECNRCNIHFYFDSDNFIDGESTCKPCPVKKIIGGVRNLGKNAKKDVQCEVQCPSGTVVQDGKAT.FENDKSECVKCAPNFYTAKPQSWVAGIDICFPCPNILIQGAKANYGTSASMATQCRV..
IMG5_190900A  178 .NFDLFSWQCVSCNYNFYFDQNEFLPGISTCKPCPVKKIIGAVYNKAEFAKKEIQCDVECPAGTVVKDGKTT.FENKKSECVKCAPNFYTEKQEDWVAGIDICFPCPNILIQGAQANYASFATHAVQCKG..
IMG5_106810A  174 .IYLSHQSECVNCNINFYLESHYFLAGESTCKPCPVKKSFGARHTLDQNAKIEVQCDVECPNGTVIYDGKTNNFKNEKSECIKCAANFNNVKQEEWVAGTDICFPCSNILIQGAQANYHSVATQATICRG..
IMG5_106830A  178 .SYVSHKNECVNCNADFYFDSFYFYAGISTCKPCPVKKTSGAIHTEGLNAQIYIQCDAECPFGTVIYDGKTTYFKNDKSECVKCATNFYTIKQNGWIAGTDTCIPCPNILILSGKANYPFSATYAAQCKI..
IMG5_190890A  178 .SYVSYKYECVNCKANFYYNSFGFQPGESSCEPCQNKKKIGAKETEGLNAKIDIQCDAECPTGTVVYDGDATY.KSDKSDCVKCAPYYYTTKQKDWVAGIDACIPCPKIENKGSKANYPQFATQSTQCNG..

IMG5_069260A  283 AAKVFSQFIQFSLIFIYLYFL. 303
IMG5_069230A  310 ...VFSQFLSFSLLFIYIYFI. 327
IMG5_069240A  310 ...VFSQFLSFSLLFICLYFIL 328
IMG5_002150A  441 ...TFAKFLSMSLIFISFYLL. 458
IMG5_203550A  487 ...TFAKFLSISLLFVSFYLL. 504
IMG5_203560A  247 ......QFLSISLLFISFYLL. 261
IMG5_080670A  314 ...I.................. 314
IMG5_069270A  454 ...N...FLSISLLLISYYLL. 468
IMG5_106820A  310 ...TFSQFLTFSLLFIYLYFL. 327
IMG5_190880A  323 ...VFSQFLKFSLLFIYFYFI. 340
IMG5_190870A  306 ...VFSQFLKFSLLFIYFYFL. 323
IMG5_018010A  306 ...VFSQLLKFSLLFIYFYFL. 323
IMG5_190900A  306 ...AFSQFLTFSLIFIYLYFL. 323
IMG5_106810A  303 ...TFSQFLTFSLLFICLYFL. 320
IMG5_106830A  307 ...VFSQFLTFSLLLIYLYFL. 324
IMG5_190890A  306 ...AFSQFFTFSLLFIYLYILL 324
```
