# Supplementary material for: Comparative genomics of the pathogenic ciliate Ichthyophthirius multifiliis, its free-living relatives and a host species provide insights into adoption of a parasitic lifestyle and prospects for disease control
Source: Genome Biol. 2011 Oct 17;12(10):R100. doi: 10.1186/gb-2011-12-10-r100 (PMC3341644; doi:10.1186/gb-2011-12-10-r100)
Supplement: Additional file 16 — Figure S5 - comparison of Ich metabolic enzymes painted on KEGG pathways with those of T. thermophila, P. tetraurelia and D. rerio. For each pathway, hyperlinks are provided to view the relevant KEGG map painted in red foreground to indicate enzymes present in Ich and green background to indicate enzymes present in other organisms. [file gb-2011-12-10-r100-S16.ZIP › Fig-S5/index.docx]

**Figure S5:** Comparison of metabolic pathways between *Danio rerio* (zebrafish ) and the three ciliates *Tetrahymena thermophila*, *Paramecium tetraurelia* and *Ichthyophthirius multifiliis* (Ich).

Mappings were done on KEGG metabolic pathway maps using the color pathways tools (http://www.genome.jp/kegg/tool/color_pathway.html).  Four digit EC numbers from all four species were painted on the same map and color-coded to distinguish the following categories:

Grey = enzyme present in all four species

Grey + Blue foreground = enzyme present in *D. rerio* and at least one other ciliate

Cyan = enzyme present in *D. rerio* but absent in all three ciliates

Yellow = enzyme present in all three ciliates but absent in *D. rerio*

Yellow + Green foreground = enzyme absent in *D. rerio* and Ich but present in one other ciliate

Yellow + Red foreground = enzyme absent in *D. rerio* but present in Ich and one other ciliate

Clicking on the hyperlinked KEGG pathway numbers below allows viewing of the color-coded enzyme mapping.  The notes below are a brief summary of the status of each pathway in the four species.

***Carbohydrate Metabolism***

[**00010**](maps/glycolysis.png) **Glycolysis/Gluconeogenesis**: Glycolysis the same in all four species and all of them can synthesize all glycolytic metabolites by gluconeogenesis (i.e. starting with oxaloacetic acid and ending with glucose-6-phosphate)

[**00020**](maps/CitrateCycle.png) **Citric Acid Cycle cycle (TCA cycle)**: Same in all four species; pyruvate dehydrogenase present in all. Interestingly, all three ciliates (but not *D. rerio*) have isocitrate lyase and malate synthase (glyoxoalate cycle – see below). These enzymes provide a bypass in the TCA cycle allowing conversion of isocitrate directly to succinate and malate.  This avoids the CO_2_-producing steps allowing more efficient assimilation of carbon from fatty acids via acetyl-CoA.

[**00630**](maps/Glyoxalate&dicarboxylate.png) **Glyoxalate and dicarboxylate metabolism**: All three ciliates have the glyoxalate cycle which is absent in *D. rerio*.

[**00030**](maps/Pentose-P_pathway.png) **Pentose phosphate pathway**: All three ciliates lack the first two enzymes of this pathway that generates NADPH. What other pathways exists in ciliates for maintaining their cytosolic NADPH levels in the absence of these pentose phosphate pathway enzymes is not clear.

[**00500**](maps/Starch&Sucrose.png) **Starch and sucrose metabolism**: All three ciliates are capable of synthesizing starch/amylopectin but not glycogen. *D. rerio* can make glycogen but not starch/amyopectin.

[**00520**](maps/Amino&Nucleotide-sugar.png)**Amino sugar and nucleotide sugar metabolism**: All four species can synthesize UDP-glucose, UDP-galactose and uridine diphosphate N-acetylglucosamine. *D. rerio* has a more comprehensive set of enzymes and can make GDP-fucose & neuraminic acid.

[**00620**](maps/Pyruvate.png) **Pyruvate metabolism**: Same in all four species.

[**00640**](maps/Propanoate.png)**Propanoate metabolism**: Major differences between *D. rerio* and ciliates. *D. rerio* metabolizes propionyl-CoA via the methylmalony-CoA pathway. Ciliates metabolize propionyl-CoA via the methyl-citric acid cycle.  Enzymes of this pathway have been studied as therapeutic targets (e.g. in *Mycobacterium tuberculosis*).

[**00562**](maps/Inositol-P.png) **Inositol phosphate metabolism**: Essentially the same in all four species with the exception that the Ich gene for CDP-diacylglycerol-inositol 3-phosphatidyltransferase (2.7.8.11), could not be found, possibly because it is located in a gap in the assembly or due to an incorrect gene model. This enzyme is required for making phosphatidyl-inositol-monophosphate from which all other phosphorylated versions are made (see below in glycerophospholipid metabolism for this enzyme mapping).

**Energy Metabolism**

[**00190**](maps/OxPhos.png) **Oxidative phosphorylation and F_1_F_0_-ATP synthase**: Pathway present in all four species.  Ciliates have an unusual F_1_F_0_-ATP synthase subunit composition (see text).  Therefore this enzyme is a potential therapeutic target.

[**00910**](maps/Nitrogen.png) **Nitrogen metabolism**: No major differences between species

[**00920**](maps/Sulfur.png) **Sulfur metabolism**: Only *D. rerio* and Ich are capable of synthesizing phosphoadenylyl sulfate.  Only Ich has the cysteine synthase enzyme which can use H_2_S as a sulfur donor for making cysteine.

**Lipid Metabolism**

[**00061**](maps/FA-synthesis.png)**Fatty acid biosynthesis**: Major differences between *D. rerio* and ciliates. All three ciliates lack both the Type I and II pathways for fatty acid biosynthesis.  *D. rerio* has the multifunctional Type I polypeptide for fatty acid synthesis.  *Paramecium* has a probable polyketide synthase that is a similar multifunctional enzyme to Type I FAS polypeptide (explaining why the coloring is grey + blue foreground for this enzyme as KEGG does not distinguish between the FAS I & PKS multifuncaitonal enzymes)

[**00062**](maps/FA-elongation.png) **Fatty acid elongation in mitochondria**: All four species are capable of elongating fatty acids.  [**00071**](maps/betaOxidation-FA.png) **Fatty acid metabolism**: All four species have all the enzymes required for fatty acid breakdown via beta-oxidation.

[**00072**](maps/ketoneBodies.png)**Synthesis and degradation of ketone bodies**: Same in all four species.

[**00100**](maps/Steroid.png)**Steroid biosynthesis**: Only *D. rerio* can convert the terpenoid isoprene into cholesterol and other steroid derivatives.  Ciliates cannot synthesize cholesterol or other steroids but can modify them.  For example ciliates can esterify cholestrol to cholestyl-esters.

[**00561**](maps/Glycerolipid.png)**Glycerolipid metabolism**: *D. rerio*, Ich and *Tetrahymena* have similar pathways for mono-, di- and tri-acylglycerol metabolism.

[**00564**](maps/Glycerophospholipid.png) **Glycerophospholipid metabolism**: *D. rerio* can make all phospholipids; Ich can make phosphatidyl-choline, phosphatidyl-ethanolamine and phosphatidyl-serine, but seems to be missing the enzyme required to make phosphatidyl-inositol (see above in inositol metabolism).

[**00600**](maps/Sphingolipid.png)**Sphingolipid metabolism**: No major difference between the four species.

[**01040**](maps/Unsaturated-FA_biosynthesis.png)**Biosynthesis of unsaturated fatty acids**: All species are capable of fatty acid desaturation.

**Nucleotide Metabolism**

[**00230**](maps/purine.png) **Purine metabolism**: Unlike *D. rerio*, ciliates are not capable of synthesizing purines, depending instead on salvage reactions. There are also interesting differences among ciliates.  Cilliates cannot convert IMP to GMP and so have to scavenge external guanine or guanosine.  Only Ich is incapable of making AMP either from adenine (*Paramecium* can do this) or adenosine (*Paramecium* and *Tetrahymena* can do this).

[**00240**](maps/pyrimidine.png) **Pyrimidine metabolism**: Unlike *D. rerio*, ciliates are incapable of synthesizing pyrimidines and depend on pyrimidine salvage for survival. In particular, uracil is made from cytosine in ciliates using cytosine deaminase, a possible therapeutic target.

**Amino Acid Metabolism**

[**00250**](maps/Alanine&Aspartate&Glutamate.png) **Alanine, aspartate and glutamate metabolism**: No major difference between species.

[**00260**](maps/Glycine&serine&Threonine.png) **Glycine, serine and threonine metabolism**: No major difference between species.

[**00270**](maps/Cysteine&Methionine.png) **Cysteine and methionine metabolism**: No major difference between species.

[**00280**](maps/Valine&Leucine&Isoleucine-degradation.png)**Valine, leucine and isoleucine degradation**: No major difference between species.

[**00290**](maps/Valine&Leucine&Isoleucine-biosynthesis.png)**Valine, leucine and isoleucine biosynthesis**: No major difference between species.

[**00300**](maps/Lysine-biosynthesis.png)**Lysine biosynthesis**: None of the four species can synthesize lysine.

[**00310**](maps/Lysine-degradation.png)**Lysine degradation**: *D. rerio*, Ich and *Tetrahymena* have similar sets of  enzymes for lysine degradation, which appear to be lacking in *Paramecium*.

[**00330**](maps/Arginine&Proline.png) **Arginine and proline metabolism**: *D. rerio* carries out the urea cycle, but this pathway is missing in all ciliates.  *D. rerio*, Ich and *Tetrahymena*, but not *Paramecium*, are capable of synthesizing proline from ornithine. Polyamine pathway: *D. rerio*, Ich and *Tetrahymena* can convert ornithine to putrescine, then to spermidine and then spermine.  *Paramecium* can make putrescine but not spermidine and spermine.

[**00340**](maps/histidine.png) **Histidine metabolism**: When compared to *D. rerio*, ciliates have limited histidine metabolism but possess the auromatic amino acid decarbosylase (AAD) enzyme that is capable of synthesizing histamine, although the biological relevance of this is not clear.

[**00350**](maps/tyrosine.png)**Tyrosine metabolism**: *D. rerio* and ciliates can break down tyrosine to acetoacetate (and with help of the AAD enzyme can decarboxylate tyrosine to tyramine & can potentially convert L-DOPA to dopamine, although they cannot synthesize L-DOPA).

[**00360**](maps/Phenylalanine.png)**Phenylalanine metabolism**: No major difference between species.

[**00380**](maps/Tryptophan.png)**Tryptophan metabolism**: No major difference between species. Interestingly ciliates are capable of converting tryptophan to serotonine and tryptamine using the AAD enzyme (see above in histidine and tyrosine metabolism).

[**00400**](maps/Pheylalanine&Tyrosine&Tryptophan-biosynthesis.png)**Phenylalanine, tyrosine and tryptophan biosynthesis**: All three ciliates have the AROM pentafunctional polypeptide which is part of the shikimic acid pathway for chorismate biosynthesis.  *D. rerio* does not have this enzymes or the pathway, making this pathway a potential drug target.

**Metabolism of Other Amino Acids**

[**00450**](maps/Selenoamino-acid.png)**Selenocysteine metabolism**: *D. rerio* and the three ciliates differ in how they make Se-Cys.  *D. rerio* can make Se-Cys from H_2_Se and acetyl-serine but ciliates can make Se-Cys from acetyl-serine only.

[**00480**](maps/Glutathione.png) **Glutathione metabolism**: All four species have the same pathway for glutathione synthesis and utilization via the oxidation/reduction cycle. Interestingly, all three ciliates seem to be capable of synthesizing trypanothione and utilizing it in oxidation/reduction cycle.

**Metabolism of Cofactors and Vitamins**

[**00670**](maps/Folate-oneCarbonPool.png)**One carbon pool by folate**: No major difference between species.

[**00860**](maps/Tetrapyrrole.png)**Porphyrin and chlorophyll metabolism**: All four species use the animal/fungal type C4 pathway for heme biosynthesis.

**[00785](maps/Lipoate.png) Lipoic acid metabolism**: Ich and other ciliates have only the salvage pathway but not the biosynthetic pathway for lipoic acid.  *D. rerio* has both.

**Others**

[**00900**](maps/Terpenoid_biosynthesis.png) **Terpenoid backbone biosynthesis**: No difference.  All four species make the terpenoid backbone using the mevalonate pathway.

[**00563**](maps/GPI-anchor.png) **Glycosylphosphatidylinositol(GPI)-anchor biosynthesis**: All four species are capable of GPI anchor biosynthesis. NOTE: many enzymes of this pathway have only three digit EC numbers.  So for Ich mapping these were left out as only four digit EC numbers were used. However, ortholog mapping suggests that most enzymes present in *Tetrahymena* are also present in ich.
