# Supplementary material for: Comparative genomics of the pathogenic ciliate Ichthyophthirius multifiliis, its free-living relatives and a host species provide insights into adoption of a parasitic lifestyle and prospects for disease control
Source: Genome Biol. 2011 Oct 17;12(10):R100. doi: 10.1186/gb-2011-12-10-r100 (PMC3341644; doi:10.1186/gb-2011-12-10-r100)
Supplement: Additional file 16 — Figure S5 - comparison of Ich metabolic enzymes painted on KEGG pathways with those of T. thermophila, P. tetraurelia and D. rerio. For each pathway, hyperlinks are provided to view the relevant KEGG map painted in red foreground to indicate enzymes present in Ich and green background to indicate enzymes present in other organisms. [file gb-2011-12-10-r100-S16.ZIP › Fig-S5/index.htm]

**Figure
S5:** 
Comparison of metabolic pathways between *Danio rerio* (zebrafish ) and the three ciliates *Tetrahymena thermophila*, *Paramecium tetraurelia* and *Ichthyophthirius multifiliis* (Ich).

 

Mappings were done on KEGG metabolic
pathway maps using the color pathways tools (http://www.genome.jp/kegg/tool/color\_pathway.html). 
Four digit EC numbers from all four species were painted on the same map and color-coded
to distinguish the following categories:

 

Grey
= enzyme present in all four species

 

Grey + Blue foreground = enzyme present in *D. rerio* and at least one other ciliate

 

Cyan
= enzyme present in *D. rerio* but
absent in all three ciliates

 

Yellow
= enzyme present in all three ciliates but absent in *D. rerio*

 

Yellow + Green foreground = enzyme
absent in *D. rerio* and Ich but
present in one other ciliate

 

Yellow + Red foreground = enzyme absent in *D. rerio* but present in Ich and one
other ciliate

 

Clicking on the hyperlinked KEGG
pathway numbers below allows viewing of the color-coded enzyme mapping. 
The notes below are a brief summary of the status of each pathway in the four species.

 

***Carbohydrate Metabolism***

**00010** **Glycolysis/Gluconeogenesis**:
 Glycolysis the same in all four
species and all of them can synthesize all glycolytic metabolites by
gluconeogenesis (i.e. starting with oxaloacetic acid and ending with glucose-6-phosphate)

**00020** **Citric Acid Cycle cycle (TCA
cycle)**:  Same in all four
species; pyruvate dehydrogenase present in all.  Interestingly, all three ciliates (but not *D. rerio*) have isocitrate lyase and
malate synthase (glyoxoalate cycle – see below). These enzymes provide a
bypass in the TCA cycle allowing conversion of isocitrate directly to succinate
and malate.  This avoids the CO2-producing steps allowing more
efficient assimilation of carbon from fatty acids via acetyl-CoA. 

**00630** **Glyoxalate and dicarboxylate
metabolism**:  All three ciliates have the glyoxalate cycle which is absent
in *D. rerio*.

**00030** **Pentose phosphate pathway**:
 All three ciliates lack the first two
enzymes of this pathway that generates NADPH.  What other pathways exists in ciliates for maintaining their
cytosolic NADPH levels in the absence of these pentose phosphate pathway enzymes
is not clear.

**00500** **Starch and sucrose metabolism**:
 All three ciliates are capable of
synthesizing starch/amylopectin but not glycogen. *D. rerio* can make glycogen but not starch/amyopectin.

**00520****Amino sugar and nucleotide
sugar metabolism**:  All four species can synthesize UDP-glucose, UDP-galactose and
uridine
diphosphate N-acetylglucosamine.  *D. rerio* has a more comprehensive set of
enzymes and can make GDP-fucose & neuraminic acid.

**00620** **Pyruvate metabolism**:
 Same in all four species.

**00640****Propanoate metabolism**:
 Major differences between *D. rerio* and ciliates.  *D.
rerio* metabolizes propionyl-CoA via the methylmalony-CoA pathway. Ciliates
metabolize propionyl-CoA via the methyl-citric acid cycle.  Enzymes of
this pathway have been studied as therapeutic targets (e.g. in *Mycobacterium tuberculosis*).

**00562** **Inositol phosphate metabolism**:
Essentially the same in all four species with the exception that the Ich gene
for CDP-diacylglycerol-inositol 3-phosphatidyltransferase (2.7.8.11), could not
be found, possibly because it is located in a gap in the assembly or due to an
incorrect gene model.  This enzyme
is required for making phosphatidyl-inositol-monophosphate from which all other
phosphorylated versions are made (see below in glycerophospholipid metabolism
for this enzyme mapping). 

 

**Energy
Metabolism**

**00190** **Oxidative phosphorylation and F1F0-ATP
synthase**: Pathway present in all four species.  Ciliates have
an unusual F1F0-ATP synthase subunit composition (see
text).  Therefore this enzyme is a potential therapeutic target.

**00910** **Nitrogen metabolism**:
No major differences between species

**00920** **Sulfur metabolism**:
Only *D. rerio* and Ich are capable of
synthesizing phosphoadenylyl sulfate.  Only Ich has the cysteine synthase
enzyme which can use H2S as a sulfur donor for making cysteine.

 

**Lipid
Metabolism**

**00061****Fatty acid biosynthesis**:
Major differences between *D. rerio*
and ciliates. All three ciliates lack both the Type I and II pathways for fatty
acid biosynthesis.  *D. rerio* has
the multifunctional Type I polypeptide for fatty acid synthesis.  *Paramecium* has a probable polyketide
synthase that is a similar multifunctional enzyme to Type I FAS polypeptide (explaining
why the coloring is grey + blue foreground for this enzyme as KEGG does not
distinguish between the FAS I & PKS multifuncaitonal enzymes)

**00062** **Fatty acid elongation in
mitochondria**: All four species are capable of
elongating fatty acids.  **00071 
Fatty acid metabolism**: All four species have all the enzymes required for fatty
acid breakdown via beta-oxidation.

**00072****Synthesis and degradation
of ketone bodies**: Same in all four species.

**00100****Steroid biosynthesis**: Only *D. rerio* can convert the terpenoid isoprene into cholesterol and
other steroid derivatives.  Ciliates cannot synthesize cholesterol or
other steroids but can modify them.  For example ciliates can esterify
cholestrol to cholestyl-esters.

**00561****Glycerolipid metabolism**:
*D. rerio*, Ich and *Tetrahymena* have similar pathways for
mono-, di- and tri-acylglycerol metabolism.

**00564** **Glycerophospholipid metabolism**:
*D. rerio* can make all phospholipids;
Ich can make phosphatidyl-choline, phosphatidyl-ethanolamine and
phosphatidyl-serine, but seems to be missing the enzyme required to make phosphatidyl-inositol
(see above in inositol metabolism).

**00600****Sphingolipid metabolism**:
No major difference between the four species.

**01040****Biosynthesis of
unsaturated fatty acids**: All species are capable of fatty
acid desaturation.

 

**Nucleotide
Metabolism**

**00230** **Purine metabolism**:
Unlike *D. rerio*, ciliates are not
capable of synthesizing purines, depending instead on salvage reactions.  There are also interesting differences
among ciliates.  Cilliates cannot convert IMP to GMP and so have to
scavenge external guanine or guanosine.  Only Ich is incapable of making
AMP either from adenine (*Paramecium*
can do this) or adenosine (*Paramecium*
and *Tetrahymena* can do this).

**00240** **Pyrimidine metabolism**:
Unlike *D. rerio*, ciliates are incapable
of synthesizing pyrimidines and depend on pyrimidine salvage for survival. In
particular, uracil is made from cytosine in ciliates using cytosine deaminase, a
possible therapeutic target.

 

**Amino
Acid Metabolism**

**00250** **Alanine, aspartate and glutamate
metabolism**: No major difference between
species.     

**00260** **Glycine, serine and threonine
metabolism**: No major difference between species.

**00270** **Cysteine and methionine
metabolism**: No major difference between species.

**00280****Valine, leucine and
isoleucine degradation**: No major difference between
species.

**00290****Valine, leucine and
isoleucine biosynthesis**: No major difference between
species.

**00300****Lysine biosynthesis**:
None of the four species can synthesize lysine.

**00310****Lysine degradation**:
*D. rerio*, Ich and *Tetrahymena* have similar sets of 
enzymes for lysine degradation, which appear to be lacking in *Paramecium*.

**00330** **Arginine and proline metabolism**:
*D. rerio* carries out the urea cycle,
but this pathway is missing in all ciliates.  *D. rerio*, Ich and *Tetrahymena*,
but not *Paramecium*, are capable of
synthesizing proline from ornithine. 
Polyamine pathway: *D. rerio*,
Ich and *Tetrahymena* can convert
ornithine to putrescine, then to spermidine and then spermine.  *Paramecium* can make putrescine but not
spermidine and spermine.

**00340** **Histidine metabolism**:  When compared to *D. rerio*, ciliates have limited histidine metabolism but possess
the auromatic amino acid decarbosylase (AAD) enzyme that is capable of synthesizing
histamine, although the biological relevance of this is not clear.

**00350****Tyrosine metabolism**:
*D. rerio* and ciliates can break down tyrosine
to acetoacetate (and with help of the AAD enzyme can decarboxylate tyrosine to
tyramine & can potentially convert L-DOPA to dopamine, although they cannot
synthesize L-DOPA).

**00360****Phenylalanine metabolism**:
No major difference between species.

**00380****Tryptophan metabolism**:
No major difference between species. Interestingly ciliates are capable of
converting tryptophan to serotonine and tryptamine using the AAD enzyme (see
above in histidine and tyrosine metabolism).

**00400****Phenylalanine, tyrosine
and tryptophan biosynthesis**: All three ciliates have the
AROM pentafunctional polypeptide which is part of the shikimic acid pathway for
chorismate biosynthesis.  *D. rerio*
does not have this enzymes or the pathway, making this pathway a potential drug
target.

 

**Metabolism
of Other Amino Acids**

**00450****Selenocysteine metabolism**:
*D. rerio* and the three ciliates differ
in how they make Se-Cys.  *D. rerio*
can make Se-Cys from H2Se and acetyl-serine but ciliates can make
Se-Cys from acetyl-serine only.

**00480** **Glutathione metabolism**:
All four species have the same pathway for glutathione synthesis and
utilization via the oxidation/reduction cycle. Interestingly, all three
ciliates seem to be capable of synthesizing trypanothione and utilizing it in
oxidation/reduction cycle.

 

**Metabolism
of Cofactors and Vitamins**

**00670****One carbon pool by folate**:
No major difference between species.

**00860****Porphyrin and chlorophyll
metabolism**: All four species use the
animal/fungal type C4 pathway for heme biosynthesis.

**00785****Lipoic acid metabolism**: Ich and other ciliates have
only the salvage pathway but not the biosynthetic pathway for lipoic
acid.  *D. rerio* has both.

 

**Others**

**00900** **Terpenoid backbone biosynthesis**:
No difference.  All four species make the terpenoid backbone using the mevalonate
pathway.

**00563****Glycosylphosphatidylinositol(GPI)-anchor biosynthesis**:
All four species are capable of GPI anchor biosynthesis. NOTE: many enzymes of
this pathway have only three digit EC numbers.  So for Ich mapping these
were left out as only four digit EC numbers were used. However, ortholog
mapping suggests that most enzymes present in *Tetrahymena* are also present in ich.
